# Pan-Cancer APOBEC Mutagenesis, Immune Microenvironment, and Clinical Outcome: A Comprehensive Multi-Omics Analysis of All Ten Family Members Across 9653 TCGA Tumors

**DOI:** 10.3390/ijms27198864

**Published:** 2026-10-05

**Authors:** Sairos Ghniem, Ilias Georgakopoulos-Soares, Apostolos Zaravinos

**Affiliations:** 1 Department of Life Sciences, School of Life and Health Sciences, European University Cyprus, Nicosia 1516, Cyprus; s.ghniem1@gmail.com (S.G.); ilias@austin.utexas.edu (I.G.-S.); 2 Cancer Genetics, Genomics and Systems Biology Laboratory, Basic and Translational Cancer Research Center (BTCRC), Nicosia 1516, Cyprus; 3 Division of Pharmacology and Toxicology, Dell Paediatric Research Institute, College of Pharmacy, The University of Texas at Austin, Austin, TX 78712, USA

**Keywords:** *APOBEC*, *APOBEC3*, cytidine deaminase, mutational signatures, TCGA, pan-cancer, tumor microenvironment, immune checkpoints, cancer stemness, biomarkers

## Abstract

The apolipoprotein B mRNA-editing catalytic polypeptide-like (*APOBEC*) family comprises ten cytidine deaminases implicated in cancer mutagenesis, yet their broader biological significance remains incompletely understood. We performed an integrated pan-cancer analysis of all ten *APOBEC* family members across 9653 tumors from 33 cancer types in The Cancer Genome Atlas (TCGA), combining mutational signatures, sequence-context enrichment, transcriptomics, immune microenvironment profiling, stemness, clinical outcomes, and somatic mutation analyses. *APOBEC* enrichment was detected in 17.6% of tumors and exhibited marked cancer-type specificity, with the highest prevalence in bladder, cervical, and head and neck carcinomas. Associations between binary *APOBEC* enrichment and advanced stage or poorer survival were largely attenuated after adjustment for cancer type. In contrast, exploratory analysis of continuous *APOBEC* burden revealed a nonlinear association with mortality, although this relationship showed substantial cancer-specific heterogeneity and requires independent validation. Coding mutations in *APOBEC* genes were rare, showed no evidence of positive selection, and were not consistently associated with altered expression, indicating that dysregulated expression rather than genetic alteration is the predominant mode of APOBEC involvement in cancer. Most *APOBEC3* genes were upregulated in tumors, particularly *APOBEC3B*, whereas *APOBEC2* was broadly downregulated. *APOBEC3A*, *APOBEC3C*, *APOBEC3D*, *APOBEC3F*, *APOBEC3G*, and *APOBEC3H* were consistently associated with immune infiltration, immune-checkpoint expression, and inflammatory immune-cell populations across multiple analytical platforms. After adjustment for tumor purity and ploidy, most *APOBEC* family members showed negative associations with stemness, whereas *APOBEC3B* displayed predominantly positive associations. Collectively, these findings reveal substantial heterogeneity within the *APOBEC* family and support evaluating individual *APOBEC* members, particularly *APOBEC3A* and *APOBEC3B*, as distinct cancer biomarkers.

## 1. Introduction

### 1.1. The APOBEC Enzyme Family

The APOBEC (apolipoprotein B mRNA-editing enzyme catalytic polypeptide-like) protein family is a group of zinc-dependent cytidine deaminases that can convert cytidine to uridine in single-stranded DNA or RNA [1]. In humans, the family comprises ten members organized into four subfamilies: APOBEC1, APOBEC2, the APOBEC3 cluster (APOBEC3A, 3B, 3C, 3D, 3F, 3G and 3H) and APOBEC4 [2]. The related activation-induced deaminase (AID) is part of the broader AID/*APOBEC* family but functions primarily in B-cell somatic hypermutation and class-switch recombination [3]. Therefore, AID is not considered further in this study. All *APOBEC* family members share a conserved zinc-coordinating deaminase domain, yet display distinct substrate preferences, expression patterns, and biological roles [1,2,3].

APOBEC1 is the catalytic subunit of the apolipoprotein B mRNA-editing complex in intestinal epithelium [4,5]. APOBEC2 is enriched in muscle tissue, where it contributes to myogenic differentiation [6,7], while APOBEC4 is the least-studied member, with expression largely restricted to the testis [8]. The seven APOBEC3 enzymes evolved primarily to restrict endogenous retroelements and exogenous retroviruses [9]. Among them, APOBEC3A and APOBEC3B are potent single-stranded DNA mutators that can access the cellular genome under dysregulated conditions, rendering them particularly relevant to cancer [10,11].

### 1.2. APOBEC-Driven Mutagenesis in Cancer

*APOBEC3A* and *APOBEC3B* preferentially deaminate cytidines at 5′-TC-3′ motifs in single-stranded DNA, generating two biochemically distinct mutation classes cataloged in COSMIC as the single-base substitution (SBS) signatures SBS2 (C → T transitions) and SBS13 (C → G transversions arising from error-prone translesion synthesis) [11,12,13]. Together, SBS2 and SBS13 are among the most prevalent mutational processes in human cancer, occurring in over 70% of cancer types and approximately half of all cancer genomes [11,12]. High APOBEC mutational burden has been associated with poor prognosis [14], enhanced genomic instability and clonal diversity [12], and altered responses to systemic therapy [12,15].

### 1.3. Tumor Immune Microenvironment and APOBEC

The tumor immune microenvironment (TME) is a critical determinant of progression, therapeutic response and prognosis [16]. Computational algorithms including TIMER [17], CIBERSORT [18], and ESTIMATE [19] enable in silico quantification of immune and stromal infiltration from bulk RNA-sequencing data. The interaction between APOBEC-associated mutagenesis and the TME is complex and potentially bidirectional: it can increase the neoantigen repertoire and enhance tumor immunogenicity, but it may also promote clonal diversification and facilitate immune escape [20,21].

### 1.4. Gaps in Knowledge and Study Objectives

Despite extensive characterization of APOBEC3A/3B biochemistry, several clinically important questions remain unresolved. First, to the best of our knowledge, the relationship between APOBEC burden and pathological stage has not been systematically evaluated across all cancer types in The Cancer Genome Atlas (TCGA) using consistent exposure estimates. Second, the prognostic value of APOBEC burden has not typically been modeled continuously, with prior work relying instead on binary classifications [22,23,24]. Third, the transcriptional behavior of all ten family members across tumor and normal tissues has not been analyzed in a unified pan-cancer framework encompassing immune infiltration, checkpoint expression, and stemness.

This study addresses these gaps through three complementary objectives: (1) to characterize APOBEC-associated mutagenesis across 9653 TCGA tumors; (2) to assess its associations with pathological stage and overall survival; and (3) to examine how individual APOBEC members relate to expression remodeling, the tumor immune microenvironment, and tumor stemness.

## 2. Results

### 2.1. Cohort Assembly and APOBEC Signature Quantification

COSMIC v3.3 reference signatures were fitted using SigProfilerAssignment. The initial dataset comprised 9909 sequenced samples. During quality control, 237 tumors were identified as having been sequenced more than once, resulting in 256 redundant measurements. For these tumors, only one measurement was retained, leaving a final cohort of 9653 tumor samples spanning 33 cancer types.

Across the final cohort, 2,228,777 somatic substitutions were profiled, with a median of 61 per tumor (interquartile range, 28–144). Of these, 196,332 (8.8%) were estimated to be attributable to SBS2 or SBS13. Non-zero exposure to SBS2 and SBS13 was fitted in 2346 and 2077 tumors, respectively, with 2647 tumors (27.4%) showing non-zero exposure to at least one signature. Among these tumors, the median number of attributed mutations was 23, with some values reaching 4992.

SBS2 and SBS13 exposures were strongly correlated across tumors (Pearson r = 0.762; Spearman ρ = 0.802; both *p* < 0.001). Moreover, the relationship remained strong when the analysis was restricted to tumors with a higher APOBEC burden (for example, ≥100 attributed mutations; Pearson *r* = 0.639), indicating that the two signatures generally increase together rather than one replacing the other.

Consistent with this, in tumors with high APOBEC burden, SBS2 and SBS13 were present in relatively similar proportions, with neither signature predominating. However, their proportions showed higher variability in cancer types with low APOBEC burden, likely because fewer mutations were available for reliable estimation.

Because SBS2 and SBS13 both arise from APOBEC-associated mutagenesis, their fitted exposures were combined into a single measure, APOBEC_total = SBS2 + SBS13. This continuous measure, referred to as APOBEC burden, was used throughout the study.

### 2.2. APOBEC Enrichment Is Highly Cancer-Type-Specific

APOBEC enrichment was assessed using the sequence-context enrichment score of Roberts et al. [25], with tumors classified as enriched at a Benjamini–Hochberg-corrected q < 0.05. By this criterion, 1702 of 9653 tumors (17.6%) were found to be APOBEC-enriched. These tumors had a median enrichment score of 2.66 and accounted for 94.1% of all SBS2 and SBS13 mutations in the cohort.

**Figure 1:**
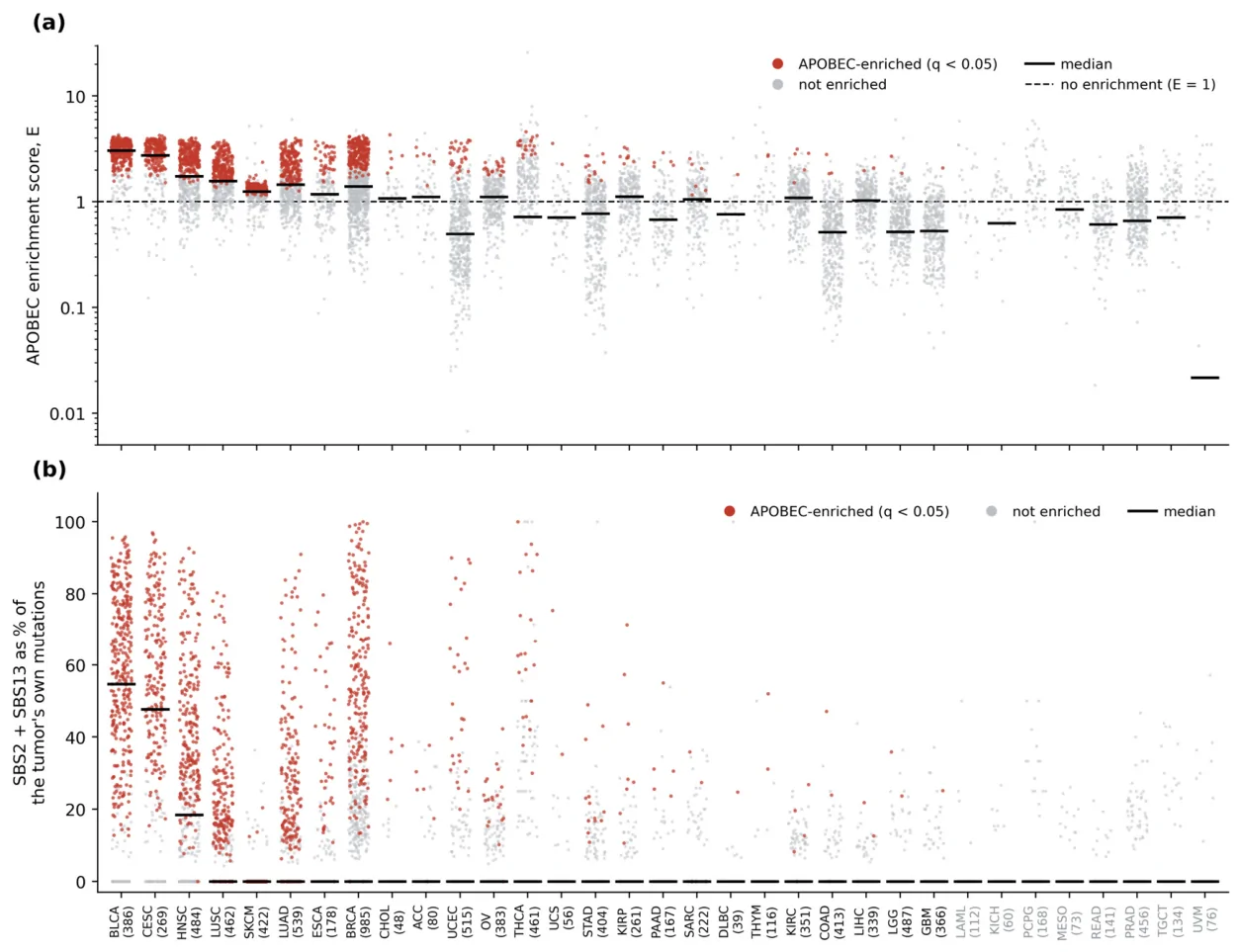
APOBEC enrichment and burden across TCGA cancer types. Each point is one tumor; red points are enriched (q < 0.05), black bars are medians. Cancer types are ordered by the percentage enriched. (**a**) tCw enrichment score E. Dashed line, E = 1. (**b**) SBS2 and SBS13 as a percentage of each tumor’s mutations.

The percentage of enriched tumors varied widely across cancer types, from 0% to 84.5% (Figure 1a; Table S1). Six cancer types exceeded 25% enrichment: bladder urothelial carcinoma (BLCA, 84.5%), cervical carcinoma (CESC, 74.0%), head and neck squamous carcinoma (HNSC, 46.9%), lung squamous carcinoma (LUSC, 44.8%), melanoma (SKCM, 37.7%), and lung adenocarcinoma (LUAD, 35.3%). Eight cancer types had no enriched tumors: acute myeloid leukemia (LAML), chromophobe renal cell carcinoma (KICH), mesothelioma (MESO), pheochromocytoma and paraganglioma (PCPG), prostate adenocarcinoma (PRAD), rectal adenocarcinoma (READ), testicular germ cell tumor (TGCT), and uveal melanoma (UVM). Cutaneous melanoma (SKCM) was a notable exception: although 37.7% of its tumors were classified as enriched, the median enrichment score among enriched melanomas was the lowest of any enriched cancer type (1.35), and APOBEC-attributed mutations accounted for a mean of only 0.7% of mutations.

### 2.3. APOBEC and Clinical Outcome

Of the 9653 tumors, 6062 had a recorded American Joint Committee on Cancer (AJCC) stage and 9244 patients had overall-survival data (2856 deaths); 5848 patients had both stage and survival data (1774 deaths).

The association between APOBEC enrichment and tumor stage was first examined. The proportion of APOBEC-enriched tumors increased from 16.9% at Stage I to 36.3% at Stage IV, with similar proportions at Stages II and III (22.0% and 22.3%).

Consistent with these findings, logistic regression showed that Stage IV tumors had 2.23-fold higher odds of APOBEC enrichment than Stages I–III (95% CI, 1.89–2.63; *p* < 0.001). However, this was attenuated after adjustment for cancer type (OR, 1.26; 95% CI, 0.98–1.63; *p* = 0.070) and further attenuated after adjustment for total mutation burden (OR, 1.10; 95% CI, 0.83–1.44; *p* = 0.51), indicating that the association was largely explained by differences in these factors (Table 1; Table S2).

Overall survival was then assessed by Cox proportional-hazards regression. In the unadjusted model, APOBEC-enriched tumors had higher mortality (HR, 1.270; 95% CI, 1.161–1.388; *p* = 1.7 × 10^−7^). This was no longer evident after adjustment for cancer type (HR, 1.035; 95% CI, 0.922–1.161; *p* = 0.558) and remained absent after further adjustment for age, sex, and total mutation burden (HR, 1.060; 95% CI, 0.940–1.194; *p* = 0.343), and after additional adjustment for stage in the subset with recorded stage (HR, 1.023; 95% CI, 0.894–1.169; *p* = 0.744) (Table 2; Table S3). The distribution of enrichment across stages and the corresponding Kaplan–Meier curves are shown in Figure S1.

Because the binary classification might not capture the magnitude of APOBEC-associated mutagenesis, APOBEC burden was next analyzed as a continuous exposure.

Most tumors had zero APOBEC-attributed mutations, and the remainder were strongly right-skewed, so several functional forms were compared by model fit. Neither a binary term nor a log-linear term improved fit over a model without an APOBEC component, whereas continuous burden did, with a three-knot restricted cubic spline providing the best fit (Table S4).

**Figure 2:**
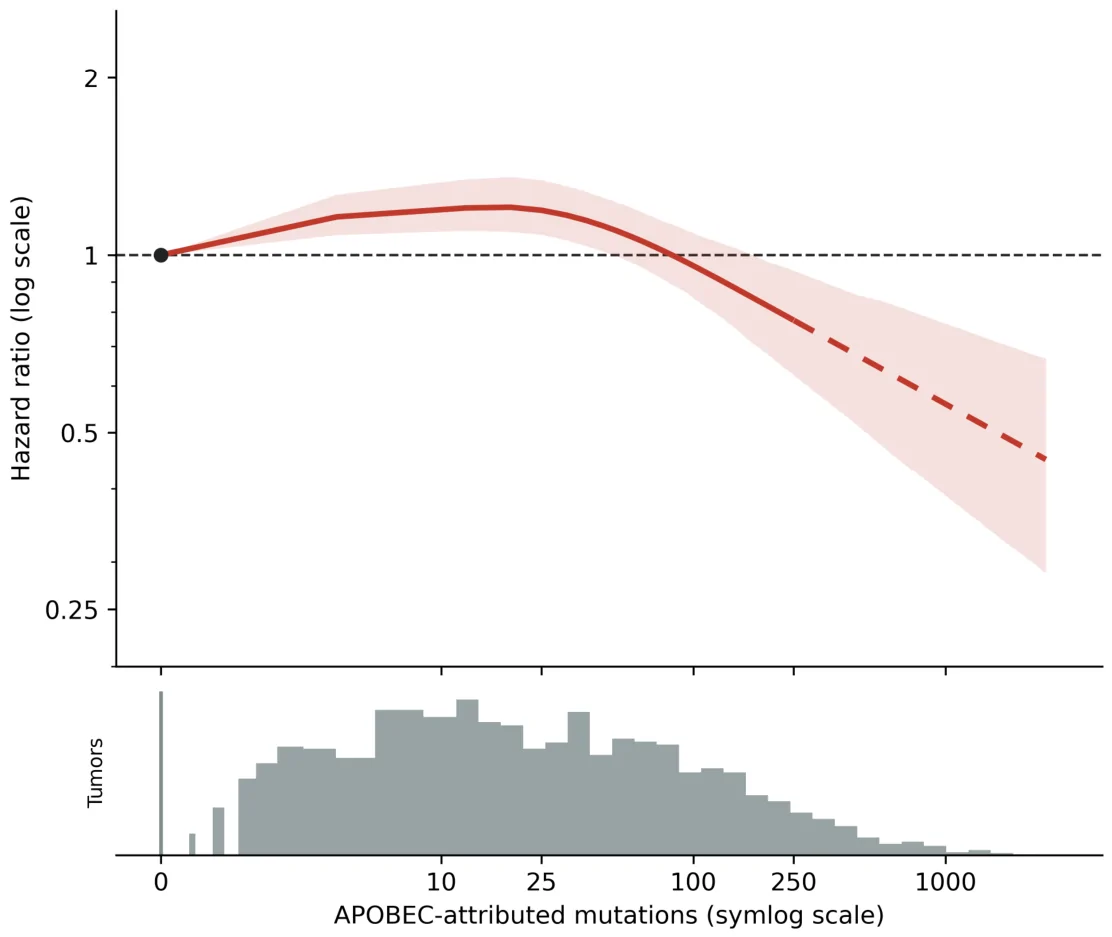
APOBEC burden and overall survival. Hazard ratios are relative to tumors with zero APOBEC-attributed mutations and were estimated using Cox models stratified by cancer type and adjusted for age and sex. The solid line shows a restricted cubic spline with knots at 5, 23, and 172 mutations (10th, 50th, and 90th percentiles), with 95% CIs. Patient numbers were: 0 (n = 6712; 1992 deaths), 1–9 (n = 679; 177 deaths), 10–49 (n = 1017; 389 deaths), 50–249 (n = 672; 247 deaths), and ≥250 (n = 164; 51 deaths). The dashed line marks the sparse range >250 mutations; the lower panel shows the burden distribution.

The spline showed a nonlinear relationship between APOBEC burden and mortality (Figure 2). In the exploratory model stratified by cancer type and adjusted for age and sex, the estimated hazard ratio, relative to tumors with zero fitted APOBEC burden, increased at low burden (1.19 at 10 mutations; 95% CI, 1.08–1.31), reached its highest level at intermediate burden, and decreased at higher burden (0.78 at 250 mutations; 95% CI, 0.63–0.95). A likelihood-ratio test supported the nonlinear model over a log-linear model (Table S13).

This nonlinear model also provided a better fit than models without an APOBEC term in both the age- and sex-adjusted analysis and the stage-adjusted subset (Table S4). The overall pattern remained after further adjustment for total mutation burden or stage, when the analysis was restricted to tumors with non-zero burden, and when APOBEC burden was expressed as the fraction of substitutions attributed to SBS2 and SBS13 rather than as an absolute count. However, the lower hazard at high burden lost statistical significance after adjustment for total mutation burden or stage and was not observed with the fraction-based measure (Tables S13 and S17). Estimates above 250 attributed mutations were less precise because relatively few patients fell within this range (n = 164); this region is therefore shown as a dashed line in Figure 2.

To assess whether the observed pattern depended on individual cancer types, we repeated the analysis after omitting each cancer type in turn. The pattern was maintained except when bladder urothelial carcinoma (BLCA) was excluded, after which the association at 250 mutations was attenuated and no longer statistically significant (HR, 0.92; 95% CI, 0.72–1.17). Consistent with this finding, the exposure–response relationship differed across cancer types (heterogeneity, *p* = 0.0003), with higher APOBEC burden associated with lower mortality in BLCA and higher mortality in lung adenocarcinoma (LUAD) (Figure S12). Therefore, the pooled spline should not be interpreted as a common exposure–response relationship. Cancer-specific spline curves are shown in Figure S13, and the full adjustment, positive-only, leave-one-cancer-out, exposure-distribution, and proportional-hazards analyses are provided in Table S13.

### 2.4. Somatic Mutation Landscape of the APOBEC Genes

Coding mutations in the ten *APOBEC* genes were rare. Across 9653 tumors spanning 33 cancer types, 464 coding mutations were identified in 321 tumors across 25 cancer types, of which 386 (83.2%) were missense mutations. Mutation prevalence ranged from 0.20% for *APOBEC3A* (19 tumors) to 0.67% for *APOBEC1* (65 tumors), and no *APOBEC* gene was mutated in more than 4.1% of tumors within any cancer type.

No coding variant was recurrently mutated. The most frequent variant, *APOBEC1* p.K69Nfs*12, was detected in five tumors, followed by *APOBEC2* p.E185K, *APOBEC3D* p.R375*, and *APOBEC3F* p.E38K and p.E215K, each detected in three tumors; all other variants occurred in two or fewer tumors.

Consistent with these low mutation frequencies, the *APOBEC* genes showed no evidence of positive selection (dN/dS, 1.04; 95% CI, 0.85–1.26; all FDR >10%; Table S5), whereas the genome-wide dN/dS in the same cohort was significantly elevated (1.073; 95% CI, 1.068–1.077), consistent with the previously reported pan-cancer estimate of 1.059 [26]. Although positive selection was detectable in the genome-wide analysis, the absence of a significant signal in individual *APOBEC* genes should be interpreted in light of their low mutation counts and the resulting limits on statistical power.

The distribution of mutations across cancer types varied between genes, with *APOBEC1* and *APOBEC3G* showing the broadest distribution (18 cancer types each), followed by *APOBEC3B* (17) and *APOBEC4* (16). In contrast, *APOBEC3A* showed the narrowest distribution, with mutations detected in seven cancer types. Other mutation classes were rare, including 39 nonsense and 22 frameshift mutations, while splice-site, in-frame deletion, translation-start-site, and nonstop mutations were observed only sporadically (Table 3; Figures S2 and S3).

### 2.5. Pan-Family Expression Remodeling in Tumor Versus Normal Tissue

Most *APOBEC3* genes were more highly expressed in tumors than in normal tissues, although the magnitude and direction of these changes varied across cancer types.

**Figure 3:**
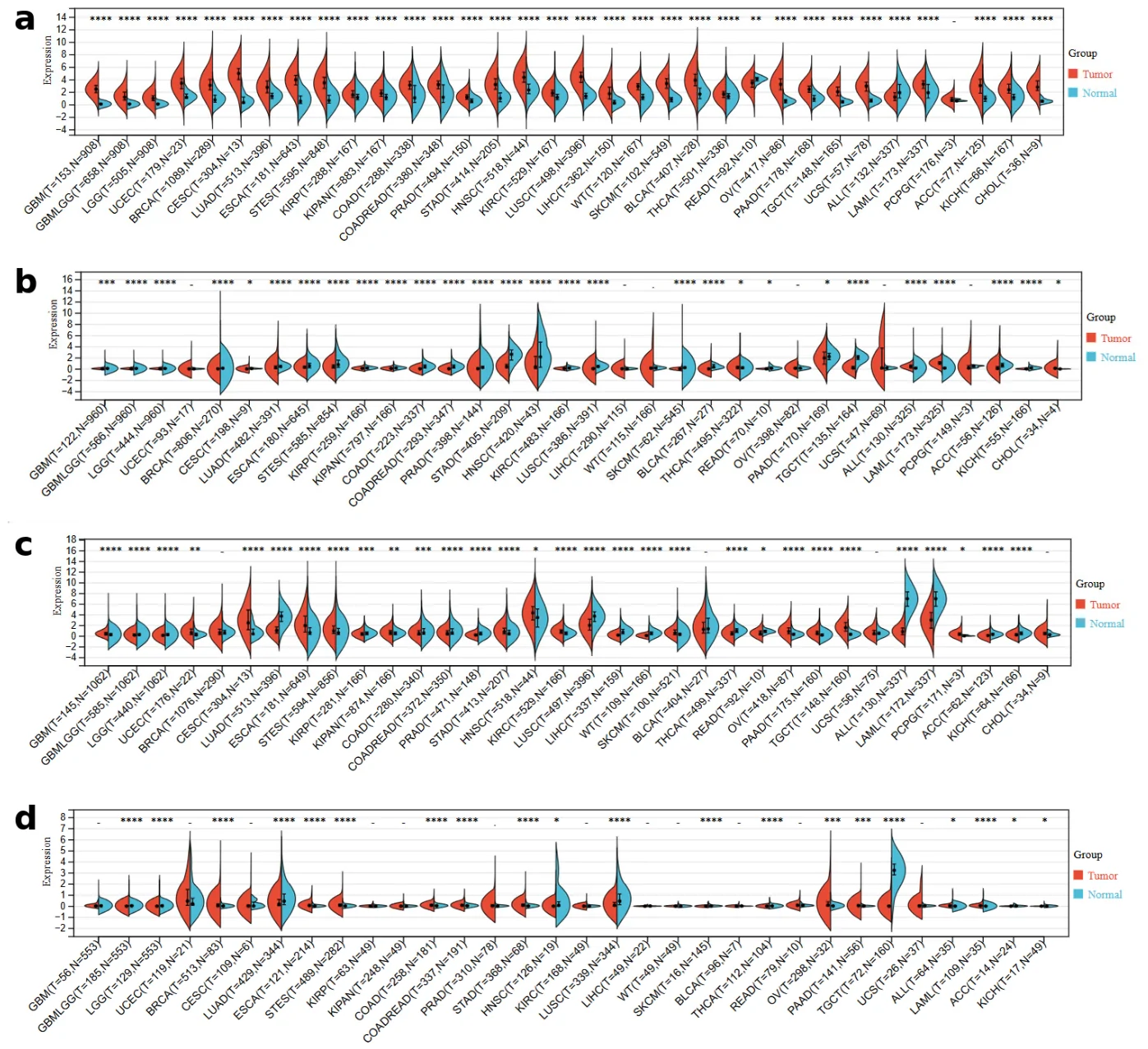
Pan-cancer tumor-versus-normal expression of representative *APOBEC* family members (split violins: tumor, red; normal, blue; nominal significance: * *p* < 0.05, ** *p* < 0.01, *** *p* < 0.001, **** *p* < 0.0001). (**a**) *APOBEC3B*, the broadest tumor upregulation in the family (31/34 cancer types). (**b**) *APOBEC2*, the broadest downregulation (24/34). (**c**) *APOBEC3A*, bidirectional regulation (14 up/16 down). (**d**) *APOBEC4*, low-level bidirectional expression with its largest single reduction in TGCT (10 up/10 down). Full tumor-versus-normal violin plots for all ten *APOBEC* family members are provided in Figure S4. Expression, log_2_(TPM + 1); sample sizes are given in Table S16.

*APOBEC3B* showed the most widespread increase, with higher expression in 31 of 34 cancer types and the largest increase overall in CESC (+3.96 log_2_; Figure 3a). *APOBEC3F*, *APOBEC3G*, *APOBEC3C*, *APOBEC3D*, and *APOBEC3H* were also frequently upregulated, occurring in 24, 23, 21, 19, and 19 cancer types, respectively. Notably, although *APOBEC3C* was upregulated in fewer cancer types than *APOBEC3B*, it had the highest tumor expression of any family member in 29 of 34 cancer types.

The expression of several *APOBEC3* genes also changed in a coordinated manner across cancers. *APOBEC3C*, *APOBEC3D*, *APOBEC3F*, *APOBEC3G*, and APOBEC3H were jointly downregulated in PRAD, KICH, and UCS but upregulated in gliomas (GBM and LGG), renal cancers (KIRC and KIRP), melanoma (SKCM), upper gastrointestinal and hepatobiliary tumors (STAD, ESCA, and CHOL), and LAML. CHOL was the only exception, with no significant upregulation of *APOBEC3H*. The strongest coordinated increase occurred in GBM, where all seven *APOBEC3* genes were significantly upregulated.

In contrast, *APOBEC3A* showed a more variable pattern, with upregulation in 14 cancer types and downregulation in 16 (Figure 3c). Its largest increase occurred in CESC (+2.14 log_2_), whereas its largest decrease occurred in ALL (−5.84 log_2_), the largest expression change observed.

*APOBEC4* showed a similarly variable pattern, with significant upregulation and downregulation in 10 cancer types each, although its expression remained low across tissues (Figure 3d). TGCT was a notable exception, showing the largest decrease (−2.88 log_2_).

The remaining genes showed more distinct patterns. *APOBEC2* was broadly downregulated, with significant decreases in 24 of 34 cancer types; LAML was the main exception (Figure 3b).

*APOBEC1* showed the least extensive remodeling, with significant upregulation mainly in gastrointestinal and pancreatic tumors (COAD, READ, STAD, and PAAD) and downregulation only in SKCM and THCA (Figure S4).

Across the family, these tumor-versus-normal differences were robust to multiple-testing correction (266 of 271 remained significant) and were largely reproduced when tumors were compared with TCGA tumor-adjacent normal tissue only (Table S16).

Finally, the relationship between *APOBEC3A* and *APOBEC3B* expression and *APOBEC* burden was examined separately across 26 cancer types. Higher *APOBEC3A* expression was significantly associated with greater *APOBEC* burden in 9 cancer types, while *APOBEC3B* showed a positive association in 11 cancer types (FDR < 0.05). No significant negative associations were observed for either gene, and correlations were generally modest (Table S18).

### 2.6. APOBEC Expression and the Immune Microenvironment

To examine the relationship between *APOBEC* expression and the tumor immune microenvironment, we correlated each *APOBEC* family member with immune features using four complementary methods: ESTIMATE, TIMER, CIBERSORT, and immune-checkpoint co-expression, across the TCGA–TARGET–GTEx cohort. All four methods showed a consistent overall pattern, with differences mainly in which immune-cell types showed the strongest associations.

#### 2.6.1. Stromal and Immune Scores (ESTIMATE)

ESTIMATE revealed a distinct immune-associated pattern among the APOBEC3 enzymes. Six of the seven APOBEC3 enzymes (APOBEC3A, APOBEC3C, APOBEC3D, APOBEC3F, APOBEC3G, and APOBEC3H) showed predominantly positive associations with the tumor microenvironment and were grouped as the “immune-associated APOBEC3 enzymes” for subsequent analyses (Table 4). Their coupling was broad, each reaching significance in 28 to 41 of the cancer types tested, with APOBEC3G and APOBEC3H the strongest (immune-score r = 0.56 and 0.57).

The remaining members were significant in far fewer cancer types and behaved differently: APOBEC3B was mixed (11 positive, 6 negative), APOBEC1 was consistently negative (0 positive, 7 negative), and APOBEC2 and APOBEC4 were weak and bidirectional, indicating limited coupling to the tumor microenvironment.

Within the immune-associated APOBEC3 group, the association was stronger for the immune compartment than for the stromal compartment. Mean correlations with immune scores were approximately r = 0.44, compared with r = 0.30 for stromal scores, and the immune-score correlation was stronger in the majority of cohorts, indicating that expression of these enzymes tracks immune infiltration more closely than general stromal content (Table S6).

The strongest single association was *APOBEC3G* in glioma (GBMLGG; r = 0.82), whereas APOBEC1 showed its strongest negative association in pancreatic adenocarcinoma (PAAD; r = −0.44) (Table 4; Figure S5).

Finally, these patterns were robust to the choice of correlation method: recomputing the correlations as Spearman rank correlations preserved the direction of every cohort-level association significant under both methods (100% directional agreement).

#### 2.6.2. Immune-Cell Lineages (TIMER)

TIMER was used to identify which immune-cell populations were associated with the immune signal observed with ESTIMATE. Of 1291 significant gene–cancer–lineage associations across 38 TIMER cohorts, 92.5% were positive (mean r = 0.32; range, −0.66 to 0.85), indicating that tumors with higher APOBEC expression generally showed greater immune infiltration.

Spearman correlations showed the same pattern (91.6% of significant associations were positive; mean ρ = 0.31; Table S7), and among associations significant by both methods, 99.8% shared the same direction.

**Figure 4:**
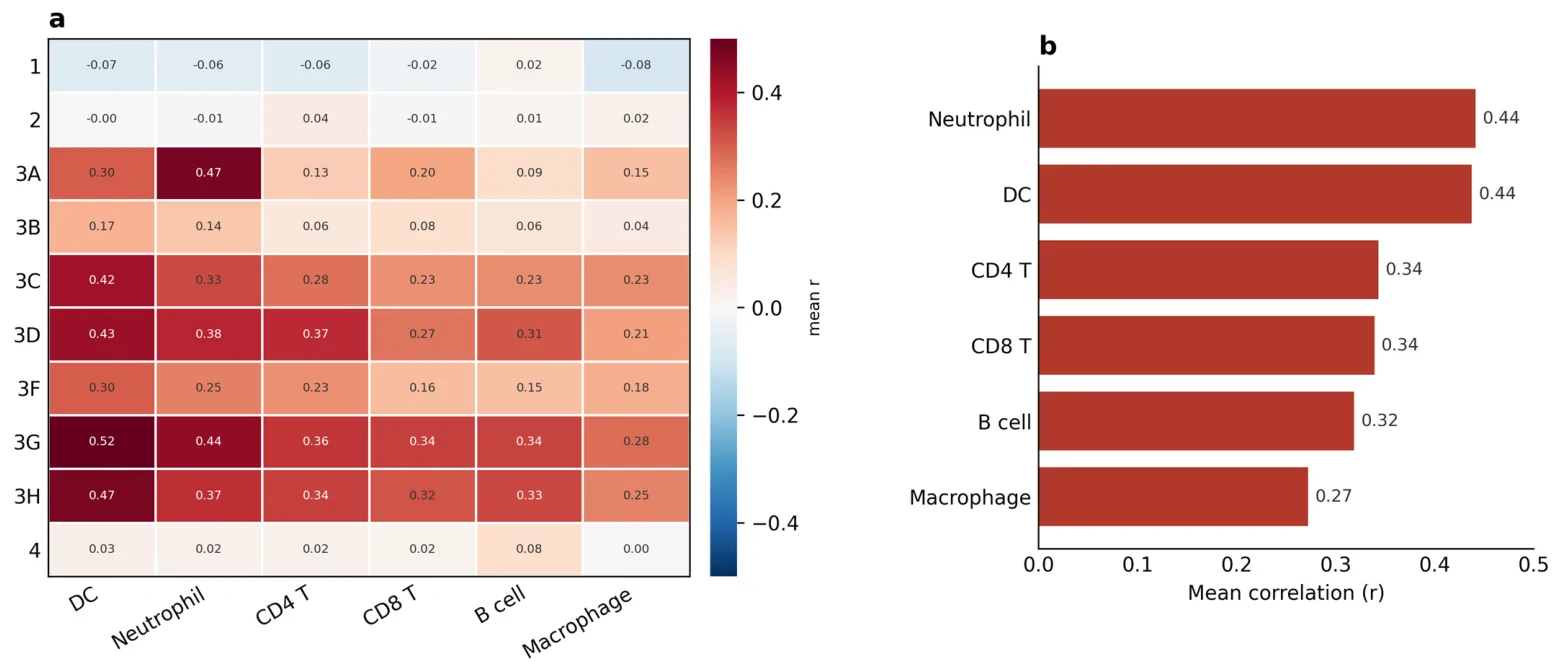
*APOBEC* expression and immune-cell infiltration (TIMER). (**a**) Mean Pearson correlation between each *APOBEC* family gene and TIMER-estimated infiltration for six immune-cell lineages, averaged across cancer types (red, positive; blue, negative). (**b**) Mean correlation by immune lineage for the six immune-associated APOBEC3 enzymes (APOBEC3A, 3C, 3D, 3F, 3G and 3H), ranked.

Among the six immune-associated APOBEC3 enzymes, dendritic cells and neutrophils showed the strongest associations (both mean *r* = 0.44; Figure 4b). Together, they were the leading lineage in 74% of significant gene–cancer comparisons (47% dendritic cells, 27% neutrophils). Associations with CD4^+^ and CD8^+^ T cells were intermediate (mean *r* ≈ 0.34), followed by B cells (*r* = 0.32), whereas macrophages showed the weakest associations (*r* = 0.27).

The neutrophil association was particularly consistent across cancers. Significant correlations were positive in every cancer type for APOBEC3A (35/35), APOBEC3C (30/30), APOBEC3F (27/27), and APOBEC3G (34/34), and in all but one for APOBEC3D (33/34) and APOBEC3H (32/33). The sole exceptions were uveal melanoma (UVM; *r* = −0.24 and −0.26, respectively; Figure S6).

APOBEC3C, APOBEC3D, APOBEC3F, APOBEC3G, and APOBEC3H behaved as a group: across cancer types, their correlation profiles with immune-cell lineages tended to increase and decrease together (mean pairwise r = 0.77). APOBEC3A followed this group’s pattern fairly well (r = 0.38–0.51), and APOBEC3B somewhat less closely (r = 0.38–0.57), whereas APOBEC1, APOBEC2, and APOBEC4 barely resembled the group at all (|r| ≤ 0.26).

In contrast to the predominantly positive associations observed for the APOBEC3 enzymes, APOBEC1 showed an inverse pattern (Table S8): its significant associations with neutrophils were consistently negative, and in pancreatic adenocarcinoma (PAAD) it was negatively associated with all six immune lineages (Figure S6).

Together, these findings suggest that the immune signal observed with ESTIMATE was most closely associated with dendritic-cell and neutrophil infiltration, particularly for the core APOBEC3 enzymes, and that APOBEC1 showed an inverse pattern.

#### 2.6.3. Immune-Cell Composition (CIBERSORT)

CIBERSORT allowed the identification of immune-cell composition at a finer resolution than TIMER by estimating 22 leukocyte subsets. Because CIBERSORT reports these subsets as relative fractions that sum to one [18], the results indicate which cell populations are relatively more or less represented rather than changes in overall immune-cell abundance (Figure S7).

**Figure 5:**
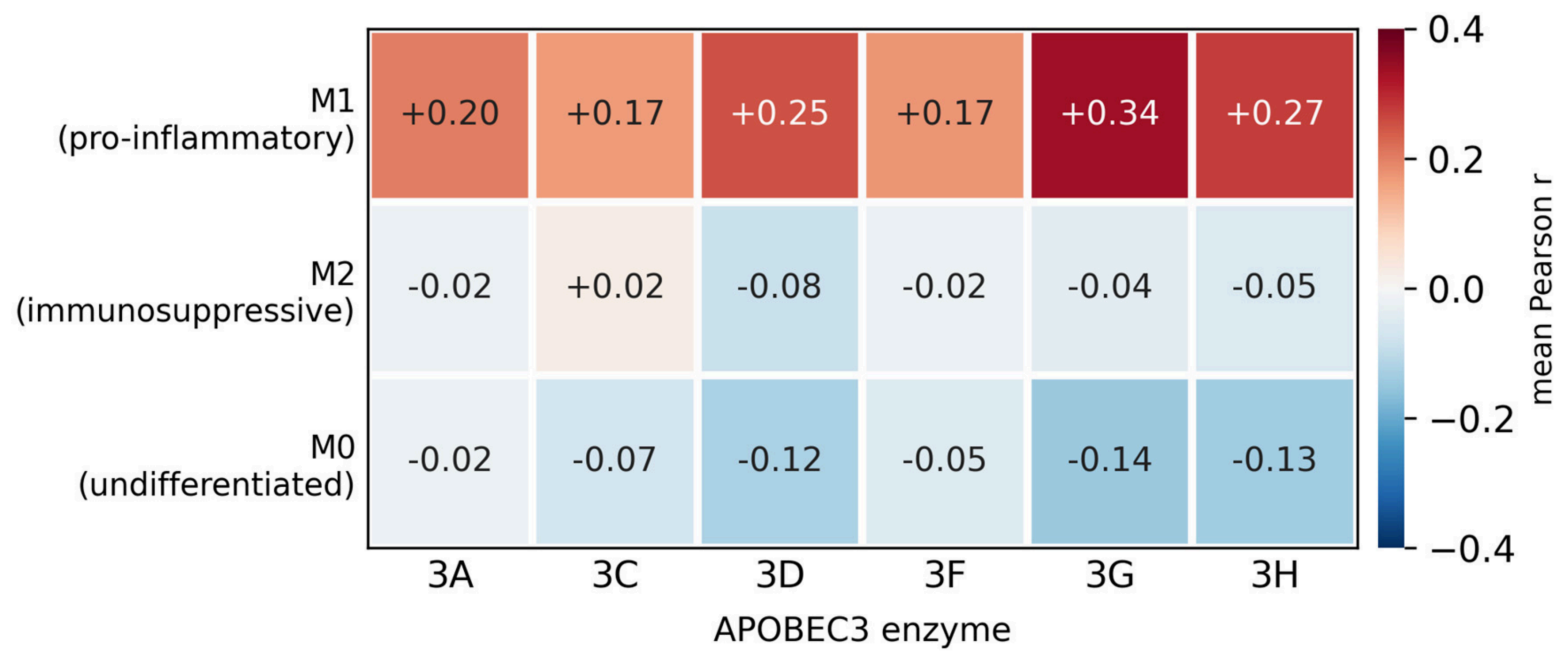
Macrophage-subtype (M1/M2/M0) associations with APOBEC3 expression (CIBERSORT).

The clearest finding involved macrophages. Unlike TIMER, which treats macrophages as a single population, CIBERSORT separates them into M1, M2, and M0 subtypes. Across the six immune-associated APOBEC3 enzymes, M1 macrophages showed consistently positive associations with APOBEC3 expression (mean *r* = +0.23; all 183 significant correlations positive), whereas M2 (mean *r* = −0.03) and M0 macrophages (mean *r* = −0.09) showed predominantly negative associations (Figure 5). Thus, the macrophage association was primarily linked to the M1 state, a distinction not captured by TIMER’s single macrophage estimate.

Beyond macrophages, the strongest positive associations were observed with cytotoxic and effector T-cell populations, particularly CD8^+^ T cells (mean *r* = +0.16) and activated CD4^+^ memory T cells (+0.14) (Figure S7; Table S9), suggesting that APOBEC3 expression was associated with an effector-rich immune composition. However, regulatory T cells were also positively associated (mean *r* = +0.06; positive in 82% of significant correlations), suggesting that this association also included a regulatory component.

APOBEC3B again differed from the other immune-associated APOBEC3 enzymes. It showed no positive association with CD8^+^ T cells (mean *r* = −0.018) and was the only gene positively associated with M0 macrophages (mean *r* = +0.08), consistent with its distinct immune-associated pattern observed elsewhere in the study.

Overall, CIBERSORT identified M1 macrophages and activated lymphoid subsets as the most prominent associations, whereas TIMER highlighted neutrophils and dendritic cells, which may be explained by differences in the cell types and reference signatures used by the two methods.

#### 2.6.4. Immune-Checkpoint Co-Expression

Immune-checkpoint co-expression reinforced the pattern observed in the other immune analyses (Figure S8). Of 12,742 significant gene–cancer–checkpoint correlations across the nine *APOBEC* genes analyzed (APOBEC4 excluded), 92.8% were positive, indicating a broad association between APOBEC expression and immune-checkpoint activity.

**Figure 6:**
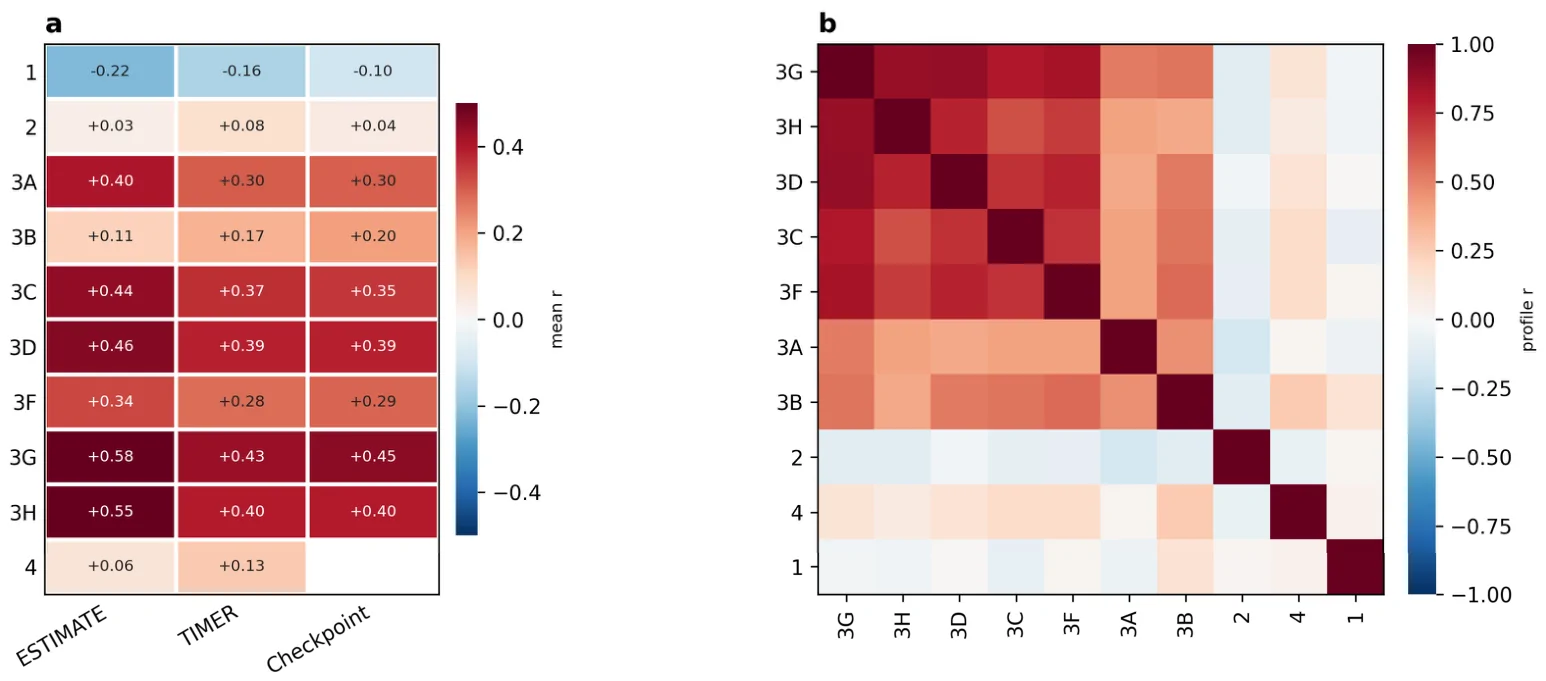
Convergent and coordinated immune associations of the immune-associated APOBEC3 enzymes. (**a**) Mean significant Pearson correlations of each *APOBEC* gene with ESTIMATE scores, TIMER immune-cell infiltration, and immune-checkpoint genes. (**b**) Pairwise concordance between *APOBEC* genes in their immune-cell lineage correlation profiles across cancer types.

The six immune-associated APOBEC3 enzymes showed the strongest and most consistent associations, with 96.7% of correlations positive and a mean *r* = 0.37. APOBEC3G showed the strongest associations, followed by APOBEC3H, APOBEC3D, APOBEC3C, APOBEC3A, and APOBEC3F. APOBEC3B was also predominantly positive but showed weaker associations (90.8% positive; mean *r* = 0.20), whereas APOBEC2 showed weak and inconsistent associations (63.5% positive; mean *r* = 0.04) and APOBEC1 showed a predominantly negative pattern (22.6% positive; mean *r* = −0.10) (Figure 6a).

The strength of co-expression also varied across checkpoint gene classes. Associations were strongest with cytotoxic and T-cell-inflamed genes, including cytotoxic effector genes (*PRF1*, *GZMA*), T-cell-recruiting chemokines (*CCL5*, *CXCL9*, *CXCL10*), and inhibitory receptors expressed by activated T cells (pooled mean *r* ≈ 0.46). In contrast, associations with angiogenic and immunosuppressive genes (*VEGFA*, *VEGFB*, *ARG1*, *VTCN1*) were weaker, although still positive (pooled mean *r* ≈ 0.11) (Figure S9; Table S10).

Importantly, clinically targeted checkpoints such as *PDCD1* (PD-1), *CD274* (PD-L1), *CTLA4*, *LAG3*, *HAVCR2* (TIM-3), *TIGIT*, and *IDO1* were positively co-expressed with the immune-associated APOBEC3 enzymes in nearly all cohorts, with mean *r* values ranging from 0.40 to 0.47.

Across the four immune analyses, the associations were unchanged after multiple-testing correction and were largely retained after adjustment for tumor purity (Tables S14 and S15).

### 2.7. APOBEC Expression and Tumor Stemness

APOBEC expression was associated with the mRNA stemness index (mRNAsi) across 8401 tumors from 33 cancer types. Because *APOBEC3* expression was also associated with tumor purity (up to *r* = −0.38 for *APOBEC3G*), correlations were adjusted for purity and ploidy (Table S11), removing approximately one-third of the unadjusted associations.

After adjustment, most *APOBEC* family members showed negative associations with stemness. *APOBEC3C*, *APOBEC3G*, *APOBEC3F*, *APOBEC4*, and *APOBEC2* showed predominantly or fully consistent negative associations across their robust cancer types (median partial *r* = −0.17 to −0.26), while *APOBEC3D* and *APOBEC3H* showed the same pattern less consistently. In contrast, *APOBEC3B* showed the opposite pattern, with positive associations in 16 of 18 robust cancer types (median partial *r* = +0.24).

*APOBEC3A* showed a marked change after adjustment, shifting from a negative association before adjustment (median *r* = −0.14) to a positive association afterward (median partial *r* = +0.19), with four associations becoming significant only after adjustment. Moreover, *APOBEC1* showed a positive association after adjustment (median partial *r* = +0.19), although this finding should be interpreted cautiously because 79.4% of its expression values were zero.

**Figure 7:**
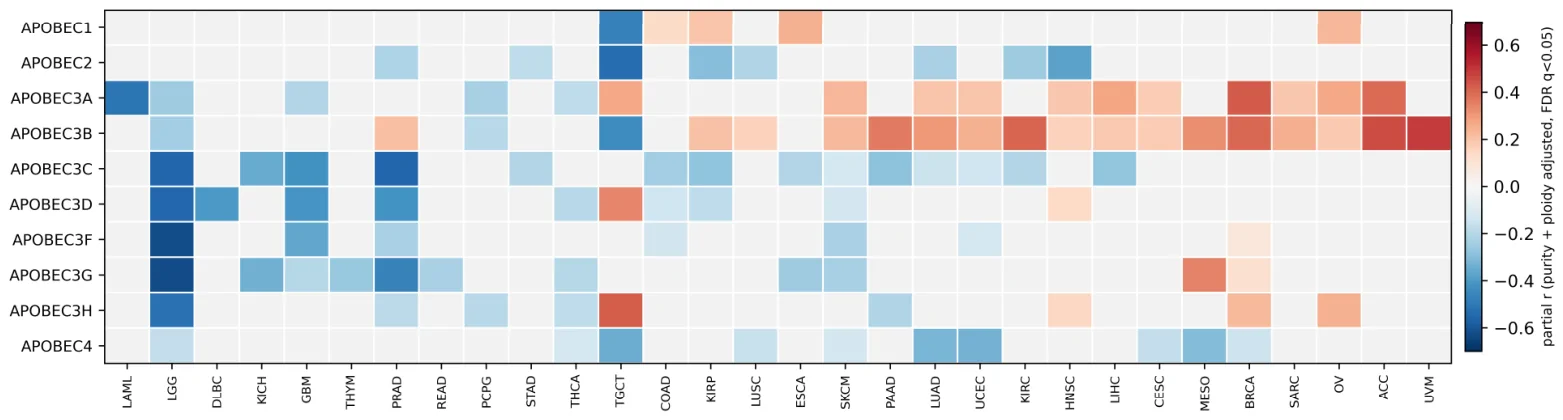
*APOBEC* expression versus tumor stemness (mRNAsi) across TCGA. Significant partial correlations (purity- and ploidy-adjusted; Benjamini–Hochberg q < 0.05) for each gene and cancer type; red, more stem-like; blue, less stem-like; non-significant pairs are blank; columns ordered by mean correlation.

The associations also varied by cancer type, with several tissues showing coordinated negative associations across multiple APOBEC3 enzymes—most clearly low-grade glioma (LGG), glioblastoma (GBM), prostate adenocarcinoma (PRAD), and thyroid carcinoma (THCA) (Figure 7). In low-grade glioma, even *APOBEC3B*, which was positively associated with stemness in most tissues, reversed to a negative association (r = −0.24), matching the strongly anti-stemness pattern of the other enzymes.

Overall, most *APOBEC* family members were negatively associated with tumor stemness, whereas *APOBEC3B* showed a distinct positive association and *APOBEC3A* changed from negative to positive after adjustment for purity.

### 2.8. Coupling Between Somatic Mutation Status and Expression

Across 45 gene–cancer-type comparisons of wild-type and non-synonymously mutated tumors, only seven reached significance (Wilcoxon *p* < 0.05; Table S12; Figure S10), all within gastrointestinal or lung cancers; none of these seven remained significant after correction for multiple testing across the 45 comparisons (Benjamini–Hochberg FDR, all q > 0.15; Bonferroni, all adjusted *p* > 0.45). However, this apparent clustering should be interpreted cautiously, as the majority of testable comparisons (29 of 45) already fell within gastrointestinal and lung cancers, reflecting the higher mutation frequency of these tumor types rather than a tissue-specific coupling effect.

In five cases, mutant tumors showed lower expression than wild-type tumors: APOBEC1 in colon and colorectal cancer (COAD and COADREAD, mutant 1.46 vs. wild-type 2.72), APOBEC3C in gastric cancer (STES, 2.96 vs. 4.51; STAD, 2.96 vs. 4.41), and APOBEC3H in lung adenocarcinoma (0.46 vs. 1.34). In the remaining two cases, mutant tumors showed higher expression than wild-type tumors: APOBEC3D in colorectal cancer (2.12 vs. 1.33) and APOBEC3G in rectal cancer (3.42 vs. 2.25).

Both directions occurred within the same tumor type; in COADREAD, APOBEC1-mutant tumors showed lower expression than wild-type tumors, whereas APOBEC3D-mutant tumors showed higher expression, indicating gene-specific rather than tissue-wide effects.

These signals were based on very small mutant groups (n = 3–8) and partially overlapping cohorts (COAD within COADREAD; STAD within STES), and should therefore be viewed as hypothesis-generating. In contrast, APOBEC3B, despite being the best-powered gene (nine cancer types; up to 974 wild-type tumors), showed no difference in expression between mutant and wild-type tumors in any cancer type, with the smallest *p*-value being 0.21, far above the significance threshold.

## 3. Discussion

### 3.1. A Unified, Pan-Family View of APOBEC Biology

This study provides a pan-cancer view of all ten *APOBEC* family members, combining mutational signature activity, sequence-context enrichment, tumor-versus-normal expression, immune microenvironment associations, tumor stemness, somatic mutation patterns, and clinical outcomes in 9653 TCGA tumors. Integrating these complementary dimensions identified relationships that are not evident from individual genes or single analytical dimensions. In particular, it revealed distinct molecular profiles of APOBEC3A and APOBEC3B and a coordinated pattern linking six APOBEC3 enzymes to the tumor immune microenvironment.

Previous pan-cancer studies have been more limited in scope. Some integrated APOBEC3 mutational signatures, expression, and immunogenicity but did not assess survival or stemness [27], while others focused only on APOBEC3B [28]. Others have examined APOBEC3 subfamily expression without linking it to survival, mutational signatures, or tumor stemness [29]. At a broader level, studies have linked APOBEC expression to immune infiltration and prognosis but did not integrate SBS2/SBS13 activity, gene-specific mutations, or stemness [30].

Three main findings emerge from this analysis. First, APOBEC enrichment was associated with more advanced stage and poorer survival, although largely explained by cancer type; when modeled continuously, APOBEC burden showed a nonlinear, cancer-type-dependent association with survival in exploratory analyses. Second, six APOBEC3 enzymes (APOBEC3A, APOBEC3C, APOBEC3D, APOBEC3F, APOBEC3G, and APOBEC3H) were consistently associated with an inflamed, checkpoint-active immune microenvironment. Third, the *APOBEC* family showed substantial biological heterogeneity, with APOBEC3A and APOBEC3B displaying distinct transcriptional, immune, and stemness-associated profiles. These findings support considering individual *APOBEC* family members separately rather than treating the family as a uniform biomarker.

### 3.2. SBS2 and SBS13 Capture Distinct Modes of the Same Mutagenic Process

SBS2 (C → T transitions) and SBS13 (C → G transversions) arise from the same initial event (cytidine deamination in single-stranded DNA) but differ in how the resulting uracil lesion is processed during replication: direct copying produces SBS2, whereas error-prone translesion synthesis produces SBS13 [11,12].

Total APOBEC burden was strongly right-skewed across the cohort, consistent with episodic bursts of APOBEC-associated mutagenesis that can generate localized clusters of mutations known as kataegis [10,11].

Within this burden, the two component signatures tracked one another closely: SBS2 and SBS13 were strongly correlated (Pearson r = 0.762; Spearman ρ = 0.802), a relationship that held across increasing burden thresholds. Both contributed similarly to the total (r = 0.943 and 0.934), and among the 498 tumors with at least 100 APOBEC-attributed mutations, 99% carried both. Together, these findings show that SBS2 and SBS13 usually occur together in APOBEC-mutated tumors rather than representing separate mutational patterns.

### 3.3. Enrichment Is Concentrated in a Handful of Lineages

Sequence-context enrichment identified 17.6% of tumors as APOBEC-enriched, with prevalence ranging from 0% to 84.5% across cancer types. Enrichment exceeded 25% in BLCA, CESC, HNSC, LUSC, SKCM, and LUAD, whereas no enriched tumors were identified in LAML, KICH, MESO, PCPG, PRAD, READ, TGCT, or UVM. Thus, APOBEC enrichment was concentrated in specific cancer types.

Importantly, APOBEC enrichment and APOBEC burden were not equivalent. Enrichment tests detect an excess of APOBEC-associated mutation contexts, and their sensitivity can increase with the overall number of mutations. This distinction was particularly evident in melanoma, where 37.7% of tumors were classified as APOBEC-enriched despite a median enrichment score of only 1.35, APOBEC-attributed mutations accounting for 0.7% of mutations on average, and only 1.9% of enriched tumors showing fitted SBS2 or SBS13 exposure. The high mutation burden of melanoma (median 399 vs. 59 substitutions in other tumors) may therefore allow relatively small excesses of APOBEC-context mutations to reach statistical significance without corresponding to a substantial APOBEC burden.

The incomplete overlap between enrichment and signature exposure further supports this interpretation. Of the 1702 APOBEC-enriched tumors, 224 (13.2%) had no fitted SBS2 or SBS13 exposure, whereas 1169 tumors with non-zero fitted SBS2 or SBS13 exposure were not classified as enriched (Figure S11). Thus, these measures capture related but distinct features of APOBEC-associated mutagenesis and should not be considered interchangeable.

### 3.4. Clinical Associations of APOBEC Enrichment and Burden

APOBEC-enriched tumors were more common at advanced stages and had shorter survival, but these associations weakened substantially after accounting for cancer type. The odds ratio for advanced stage decreased from 2.23 to 1.26 after adjustment for cancer type and to 1.10 after further adjustment for total mutation burden. Similarly, the survival hazard ratio decreased from 1.270 to 1.035 after adjustment for cancer type and remained close to the null after further adjustment. Thus, the associations between binary APOBEC enrichment, advanced stage, and poorer survival were largely explained by differences between cancer types.

When APOBEC burden was modeled continuously, exploratory analyses showed a nonlinear association with survival. Estimated mortality was higher at low-to-moderate burden (HR = 1.19 at 10 mutations) and lower at higher burden (HR = 0.78 at 250 mutations). The nonlinear shape was retained across adjustment models, although the lower hazard at high burden was attenuated and no longer significant after accounting for total mutation burden or stage. The lower hazard at high burden was largely attributable to bladder urothelial carcinoma and was not observed consistently across other major APOBEC-associated cancer types (Figures S12 and S13). Thus, the data support a nonlinear association between APOBEC burden and survival but not a consistent survival benefit at high burden; the latter should be considered hypothesis-generating.

The nonlinear pattern may provide one explanation for inconsistent survival associations reported in studies using binary APOBEC classifications [22,23,24]. One possibility is that moderate APOBEC-associated mutagenesis may promote tumor progression through increased genomic instability, whereas very high levels may increase neoantigen production and immune recognition [31,32]. However, our data cannot establish this mechanism, and the nonlinear association requires validation in independent cohorts.

### 3.5. The APOBEC Genes Are Dysregulated, Not Mutated

*APOBEC* genes appear to contribute to cancer mainly through altered expression and enzymatic activity rather than mutations in the genes themselves. Coding mutations were rare (0.20–0.67% of tumors per gene), were mostly missense, and no recurrent mutations were identified. None showed evidence of positive selection by dN/dS (all FDR > 10%), unlike the genome-wide signal in the same cohort (dN/dS = 1.073), consistent with *APOBEC* genes not being major recurrent cancer drivers [33].

Mutations also did not appear to substantially affect *APOBEC* expression. Only seven of 45 gene–cancer comparisons were nominally significant, none remained significant after correction, and all involved just 3–8 mutated tumors. *APOBEC3B*, the most consistently overexpressed family member, showed no significant expression difference between mutated and non-mutated tumors in any of the nine cancer types examined (smallest *p* = 0.21). Thus, the main change in *APOBEC* genes in cancer appears to be altered expression and activity, rather than frequent genetic mutation of the genes themselves.

### 3.6. Expression Remodeling in Tumor Versus Normal Tissue

Most *APOBEC3* genes showed higher expression in tumors than in normal tissue, but the extent of dysregulation varied across cancer types. *APOBEC3B* showed the broadest upregulation, occurring in 31 of 34 cancer types, whereas *APOBEC3C* had the highest tumor expression in 29 of 34 cancer types. Together, these findings indicate that *APOBEC3* upregulation is common in cancer, but the pattern varies across family members and cancer types.

This heterogeneity was accompanied by coordinated regulation among several *APOBEC3* genes. *APOBEC3C*, *APOBEC3D*, *APOBEC3F*, *APOBEC3G*, and *APOBEC3H* were jointly downregulated in prostate adenocarcinoma (PRAD), chromophobe renal carcinoma (KICH), and uterine carcinosarcoma (UCS), but jointly upregulated in gliomas, renal cancers, melanoma, upper gastrointestinal and hepatobiliary tumors, and acute myeloid leukemia; in glioblastoma (GBM), all seven *APOBEC3* genes were upregulated. This coordination suggests a shared regulatory input rather than gene-specific control. Interferon signaling is one plausible contributor, as it induces several *APOBEC3* genes [34,35] and, more broadly, shapes the composition and activity of the tumor immune microenvironment [36], potentially linking the coordinated expression changes to the immune associations observed in this study.

*APOBEC3A* showed a more variable pattern, with upregulation in 14 and downregulation in 16 cancer types. Its largest decrease occurred in acute lymphoblastic leukemia (−5.84 log_2_), which may partly reflect the high expression of *APOBEC3A* in normal myeloid cells and blood [34,35]. This highlights the importance of cellular and tissue context when interpreting tumor-versus-normal expression.

*APOBEC2* and *APOBEC1* showed distinct patterns. *APOBEC2* was broadly downregulated (24/34 cancer types), whereas *APOBEC1* showed more limited changes, mainly in gastrointestinal and pancreatic tumors, consistent with its tissue-specific role in mRNA editing [4].

Overall, these findings suggest that *APOBEC* genes are shaped by both shared regulatory signals and gene- and tissue-specific effects.

### 3.7. The Immune-Associated APOBEC3 Enzymes Mark an Inflamed, Immune-Activated Microenvironment

The four immune analyses consistently linked APOBEC3 expression to an immune-infiltrated tumor microenvironment, although the specific immune features varied between methods. ESTIMATE showed stronger associations with the immune than the stromal component, while TIMER and CIBERSORT both showed predominantly positive associations with immune-cell populations. TIMER highlighted dendritic cells and neutrophils, whereas CIBERSORT identified M1 macrophages, CD8^+^ T cells, and activated CD4^+^ memory T cells as the strongest associations. These differences likely reflect the different reference signatures and resolutions of the two methods [37]. Despite these differences, the overall pattern indicates greater immune involvement in tumors with higher APOBEC3 expression.

The immune pattern included both activation and regulation. CIBERSORT showed positive associations with CD8^+^ T cells, activated CD4^+^ memory T cells, M1 macrophages, and regulatory T cells, while M0 and M2 macrophages showed weak or negative associations. Checkpoint analysis showed a similar pattern, with 92.8% of significant APOBEC–checkpoint correlations being positive. The six immune-associated APOBEC3 enzymes were positively associated with immune-response markers such as *PRF1*, *GZMA*, *CCL5*, *CXCL9*, and *CXCL10*, as well as inhibitory checkpoint genes. Together, these findings are consistent with an immune-active, T-cell-inflamed microenvironment in which immune activation is accompanied by regulatory signals [38,39].

Several mechanisms could contribute to this relationship. APOBEC-associated mutagenesis may increase neoantigen generation and immune recognition [32], while APOBEC activity may also promote immune signaling through pathways such as cGAS–STING [40,41]. Conversely, interferon signaling can induce several *APOBEC3* genes [34,35], raising the possibility that immune signaling also drives APOBEC3 expression. Thus, APOBEC activity and immune signaling may influence each other in a bidirectional manner. Consistent with this possibility, several APOBEC3 enzymes showed similar immune profiles (Figure 6b), suggesting that they may be subject to shared regulatory mechanisms. However, the present data cannot determine the direction of this relationship or establish causality.

An important consideration when interpreting this concordance is that *APOBEC3* genes are themselves expressed by immune cells, so in bulk RNA-seq higher APOBEC3 expression may partly reflect greater immune-cell content rather than tumor-cell expression. This shared origin could inflate the observed correlations with immune metrics derived from the same data, and single-cell or purity-resolved expression would be needed to separate tumor-intrinsic from infiltrate-derived APOBEC3 expression.

### 3.8. Tumor Stemness, and Where the Family-Wide Pattern Breaks Down

Across 8401 tumors from 33 cancer types, adjusting for tumor purity and ploidy substantially altered the relationship between APOBEC expression and stemness, removing approximately one-third of the unadjusted associations and changing the direction of some associations. This highlights the influence of non-tumor cell content on bulk-tumor expression [42].

After adjustment, most *APOBEC* genes were negatively associated with stemness. APOBEC3C, APOBEC3G, APOBEC3F, APOBEC4, and APOBEC2 showed predominantly negative associations, whereas APOBEC3B was the clear exception, with positive associations in 16 of 18 robust cancer types (median partial *r* = +0.24). This may partly relate to its cellular distribution: APOBEC3B is predominantly nuclear [43] and showed the weakest correlation with tumor purity in our data (pan-cancer *r* = −0.12 vs. −0.21 to −0.38 for the other *APOBEC3* genes). Its expression may therefore be less influenced by non-tumor cell content and more reflective of tumor-cell biology, although the mechanism underlying its positive association with stemness remains unclear.

Moreover, APOBEC3A showed a marked change after adjustment, shifting from a negative to a positive association with stemness. Because APOBEC3A is strongly expressed in myeloid cells and induced by interferon signaling [34], its unadjusted association may partly reflect differences in immune-cell composition. This reversal illustrates how tumor composition can alter, and even invert, associations estimated from bulk expression data.

The relationship between APOBEC expression and stemness also varied across cancer types. Low-grade glioma (LGG), glioblastoma (GBM), prostate adenocarcinoma (PRAD), and thyroid carcinoma (THCA) showed coordinated negative associations across several *APOBEC3* genes. Notably, APOBEC3B was also negatively associated with stemness in these cancers despite its generally positive association elsewhere. This tissue-specific pattern warrants further study.

TGCT showed a distinct pattern, with both positive and negative APOBEC–stemness associations rather than a coordinated response. This finding should be interpreted cautiously because mRNAsi reflects similarity to embryonic stem-cell programs, while TGCTs arise from cells with pluripotent properties and their precursor lesions already show stem-cell-like expression patterns [44,45]. Thus, a high mRNAsi score in TGCTs may partly reflect developmental origin rather than acquired dedifferentiation.

### 3.9. APOBEC3A and APOBEC3B Display Distinct Transcriptional, Immune, and Stemness-Associated Profiles

Although APOBEC3A and APOBEC3B are the two catalytically active APOBEC3 enzymes capable of genome mutagenesis, they diverged sharply across our analyses.

APOBEC3B was the most consistently upregulated family member in tumor versus normal tissue, whereas APOBEC3A showed a much more variable expression pattern. Moreover, their immune associations differed: APOBEC3A closely tracked immune infiltration and checkpoint expression, whereas APOBEC3B showed weaker and less consistent associations with the tumor microenvironment.

The clearest difference was in their relationship with stemness. After adjustment for tumor purity and ploidy, APOBEC3B showed predominantly positive associations with mRNAsi among the associations classified as robust, while APOBEC3A also showed positive associations in several cancers, indicating that this pattern was not exclusive to APOBEC3B. Together, these findings indicate that APOBEC3A and APOBEC3B differ across transcriptional, immune, and stemness measures; whether this reflects mechanistic divergence would require experimental validation.

This distinction has practical implications. APOBEC3A and APOBEC3B are highly similar in sequence, particularly within their catalytic domains, which can make them difficult to distinguish with some antibody- and expression-based assays [13,34]. Combining them into a single APOBEC score could therefore obscure differences in their associations with immune features and stemness. These findings support measuring APOBEC3A and APOBEC3B separately in biomarker and prognostic studies.

### 3.10. Limitations

Several limitations should be considered. First, this observational study relies mainly on bulk transcriptomic and exome data, which limits causal inference and makes it difficult to identify the cell types expressing *APOBEC* genes. Immune-cell estimates also represent relative rather than absolute proportions. Single-cell and spatial studies will be needed to resolve these relationships. The high sequence similarity between APOBEC3A and APOBEC3B may also limit their separation by expression-based assays, requiring targeted validation. In addition, expression was analyzed at the gene level and therefore does not capture the isoform-level heterogeneity revealed by long-read sequencing [46,47].

Second, APOBEC enrichment reflects a significant excess of APOBEC-context mutations but does not indicate that APOBEC is the dominant mutational process. This is particularly important in highly mutated tumors, where modest enrichment may reach statistical significance.

Third, the large number of comparisons and some small cohorts require caution when interpreting individual associations. The strongest findings are those that were consistent across complementary analyses.

Finally, the study is based mainly on TCGA, TARGET, and GTEx data, and the tumor-versus-normal comparisons combined normal tissue from TCGA and GTEx, which differ in sample collection and processing. Independent cohorts and functional studies are needed to validate the findings, particularly the clinical associations.

## 4. Materials and Methods

### 4.1. Data Sources and Cohort Assembly

Somatic mutation and clinical data were obtained from The Cancer Genome Atlas (TCGA) via the Genomic Data Commons (GDC) and UCSC Xena. Mutational-signature, enrichment, stage and survival analyses used 9653 tumor samples spanning 33 cancer types. Where a tumor had been sequenced more than once, the original tumor DNA sample was retained and amplified-DNA replicates discarded, leaving one measurement per tumor. Clinical annotation was obtained from the GDC pan-cancer clinical dataset.

### 4.2. Mutational Signature Quantification

Mutational signatures were quantified by fitting COSMIC v3.3 reference signatures to individual tumors using SigProfilerAssignment. SBS96 mutation matrices were generated with SigProfilerMatrixGenerator against GRCh38 using the exome setting, which was also used for signature fitting. APOBEC-associated mutagenesis was quantified using two complementary measures. First, APOBEC burden was defined as the combined contribution of SBS2 and SBS13 (APOBEC_total = SBS2 + SBS13). Absolute counts were used to represent the cumulative number of mutations attributed to these signatures.

Second, tumors were classified as APOBEC-enriched using the sequence-context enrichment method described by Roberts et al. [25]. Enrichment was tested using a one-sided Fisher’s exact test with Benjamini–Hochberg correction, with *q* < 0.05 considered significant. APOBEC enrichment was defined as a significant excess of mutations in the APOBEC-preferred tCw context and was used as a binary classification, consistent with previous studies [22,24].

### 4.3. Survival and Stage Analysis

The relationship between APOBEC enrichment and tumor stage was examined in 6062 tumors with available AJCC stage information, categorized as Stages I–IV. Stage IV was compared with Stages I–III using logistic regression in three models: unadjusted, adjusted for cancer type, and adjusted for both cancer type and log-transformed total mutation burden. Models included only cancer types containing both APOBEC-enriched and non-enriched tumors.

Overall survival was defined as the time from diagnosis to death from any cause. After excluding patients with missing follow-up time or age, 9244 patients remained, including 2856 deaths. Survival differences between APOBEC-enriched and non-enriched tumors were assessed using Kaplan–Meier curves and the log-rank test. Cox models were fitted with sequential adjustment for cancer type, age, sex, and total mutation burden. A further model additionally adjusted for AJCC stage in the 5848 patients with available staging data. The proportional-hazards assumption was assessed using scaled Schoenfeld residuals.

To determine whether the relationship between APOBEC burden and survival was nonlinear, several models were tested, including binary, categorical, transformed, and restricted cubic spline models. Model fit was compared using the Akaike information criterion and likelihood-ratio tests, and nonlinearity was assessed by comparing the spline model with the corresponding log-linear model.

The spline model was then tested for robustness by adjusting for total mutation burden and AJCC stage, restricting the analysis to tumors with non-zero APOBEC burden, and repeating the analysis after removing each cancer type separately. Knots were placed at the 10th, 50th, and 90th percentiles of non-zero APOBEC burden (5, 23, and 172 mutations), with confidence intervals obtained by bootstrap resampling. All burden models were stratified by cancer type, allowing the baseline hazard to vary across cancer types. Between-cancer heterogeneity in the exposure–response relationship was assessed using a likelihood-ratio test comparing models with and without a burden-by-cancer-type interaction. Cancer-specific splines were additionally fitted for the major APOBEC-associated cancer types (BLCA, CESC, HNSC, LUAD, and LUSC). As an alternative exposure definition, the analysis was repeated using the fraction of single-base substitutions attributed to SBS2 and SBS13, (SBS2 + SBS13)/total substitutions, which normalizes for differences in total mutation burden (Table S17).

### 4.4. Somatic Mutation Landscape of the APOBEC Genes

Non-synonymous mutations in *APOBEC* family genes were analyzed to assess their frequency and recurrence across cancer types. Mutation frequency was calculated as the percentage of tumors carrying at least one mutation, and recurrent protein variants were identified.

Selection was assessed using dNdScv [26] by estimating the ratio of non-synonymous to synonymous substitutions (dN/dS). Genes with a false discovery rate <10% were considered to show evidence of positive selection.

### 4.5. Differential Expression and Immune Microenvironment Analysis

Expression data for the *APOBEC* family genes were obtained from the UCSC Xena TCGA–TARGET–GTEx dataset [48], in which all samples were uniformly re-processed (RSEM, transcripts per million, TPM), and were analyzed as log_2_(TPM + 1) across 34 cancer types. Normal samples were tumor-adjacent normal tissue from TCGA and/or normal tissue from GTEx (tissue-matched rather than patient-matched; Table S16). Tumor-versus-normal expression was compared within each cancer type using the unpaired Wilcoxon rank-sum test and, as a sensitivity analysis, against TCGA tumor-adjacent normal tissue only, in cancer types with at least 10 such samples. Associations with the immune microenvironment were assessed using ESTIMATE, TIMER, and CIBERSORT. ESTIMATE provided immune and stromal scores, TIMER estimated six major immune-cell populations, and CIBERSORT estimated the relative fractions of 22 immune-cell subsets using the LM22 signature matrix.

Pearson correlations between APOBEC expression and immune features were calculated separately for each cancer type. Spearman correlations were also calculated for ESTIMATE, TIMER, and CIBERSORT as a robustness check. Associations with *p* < 0.05 were considered significant. To assess agreement across methods, the mean correlation of each gene under ESTIMATE, TIMER, and checkpoint co-expression was compared, and between-gene concordance was quantified by correlating each gene’s profile of immune-lineage associations across cancer types with that of the other family members (mean pairwise Pearson r).

Immune-checkpoint co-expression was assessed using 60 checkpoint genes from Thorsson et al. [49]. APOBEC4 was excluded because checkpoint data were not available for this gene. For each remaining *APOBEC* gene, correlations with individual checkpoint genes were calculated across cancer types, and checkpoint genes were ranked according to their mean significant correlation with the six immune-associated APOBEC3 enzymes (APOBEC3A, 3C, 3D, 3F, 3G, and 3H).

To assess the influence of non-tumor cell content, the immune correlations were recomputed in TCGA tumors as partial correlations adjusted for ABSOLUTE tumor purity, using per-sample ESTIMATE scores, TIMER2.0 estimates [50], CIBERSORT fractions [49], and TCGA PanCanAtlas expression data (Table S15).

To link expression with the mutational phenotype, APOBEC3A and APOBEC3B expression was correlated with APOBEC burden within each cancer type (Spearman correlation; Table S18); cancer types with fewer than 30 tumors or fewer than 10 tumors with non-zero burden were excluded.

### 4.6. Tumor Stemness Analysis

The mRNA-based stemness index (mRNAsi) from Malta et al. [51] was used as a measure of tumor stemness, with higher values indicating greater similarity to an embryonic stem-cell expression program. Within each cancer type, Pearson correlations between APOBEC expression and mRNAsi were calculated before and after adjustment for tumor purity and ploidy using partial correlation. Purity was included as non-tumor cells contribute to bulk expression, and ploidy as copy-number state can alter bulk expression independently of cell composition.

*p* values were corrected across gene–cancer comparisons using the Benjamini–Hochberg procedure. An association was considered robust to adjustment if it remained significant after correction (q < 0.05).

### 4.7. Mutation-Status and Expression Coupling

To determine whether non-synonymous mutations in *APOBEC* genes were associated with altered expression of the corresponding gene, expression was compared between tumors with and without a mutation in each *APOBEC* gene. Within each cancer type, the two groups were compared using the two-sided Wilcoxon rank-sum test. Cancer types with fewer than three samples were excluded.

Significance was assessed at *p* < 0.05, with *p* values adjusted for multiple comparisons using the Benjamini–Hochberg and Bonferroni methods.

### 4.8. Statistical Analysis, Software, and Code Availability

Analyses were performed in Python (v3.10), R (v3.6.3), and SPSS (v27), using the relevant packages for mutational signatures, survival analysis, mutation analysis, immune deconvolution, and correlation testing. All analysis code and the derived datasets are available at Zenodo (DOI: 10.5281/zenodo.22676594). Multiple testing was corrected with the Benjamini–Hochberg method within each analysis (APOBEC enrichment, tumor-versus-normal expression, immune associations, stemness, and APOBEC3A and APOBEC3B expression versus APOBEC burden); mutation-status comparisons were additionally corrected with the Bonferroni method. Survival and stage analyses are reported with 95% confidence intervals and nominal *p* values, and figures of tumor-versus-normal expression and immune associations show nominal significance.

During manuscript preparation, Claude (Anthropic) was used to assist with numerical verification, copy-editing, and formatting. All outputs were reviewed and edited by the authors, who take full responsibility for the final content.

## 5. Conclusions

Across 9653 TCGA tumors, APOBEC-associated mutagenesis was highly cancer-type-specific, with enrichment concentrated in bladder, cervical, and head and neck carcinomas. Coding mutations in *APOBEC* genes were rare and showed no evidence of positive selection, indicating that their involvement in cancer is primarily associated with altered expression and enzymatic activity rather than genetic alteration of the APOBEC loci.

Binary APOBEC enrichment was associated with stage and survival before adjustment, but these associations were largely explained by cancer type. In contrast, continuous APOBEC burden showed a nonlinear association with survival; however, the lower hazard at high burden was not robust to further adjustment, differed across cancer types, and requires validation in independent cohorts. Thus, how APOBEC-associated mutagenesis is measured substantially affects its clinical associations and may help explain conflicting findings from previous studies.

The *APOBEC* family also showed distinct relationships with the tumor microenvironment and stemness. APOBEC3A, 3C, 3D, 3F, 3G, and 3H were consistently associated with an immune-infiltrated, checkpoint-engaged microenvironment, whereas most family members were negatively associated with stemness after adjustment for tumor purity and ploidy. APOBEC3B was the clear exception, showing a robust positive association with stemness and weaker immune associations.

Overall, APOBEC3A and APOBEC3B showed distinct expression, immune, and stemness patterns, supporting their treatment as separate biomarkers rather than interchangeable components of a single APOBEC signal. Future studies using single-cell and spatial approaches, together with independent clinical cohorts, are needed to define the cellular basis of these associations and assess their prognostic relevance.

## Figures and Tables

**Table 1:** APOBEC enrichment and tumor stage. Logistic regression comparing Stage IV with Stages I–III.

Model	n	OR	95% CI	*p*-Value
Unadjusted	5657	2.23	1.89–2.63	2.8 × 10^−21^
+ cancer type	5657	1.26	0.98–1.63	0.070
+ cancer type + log mutation burden	5657	1.10	0.83–1.44	0.51

**Table 2:** APOBEC enrichment and overall survival. Cox proportional-hazards models. The stage-adjusted model is restricted to the 5848 patients with a recorded AJCC stage.

Model	n	HR	95% CI	*p*-Value
Unadjusted	9244	1.270	1.161–1.388	1.7 × 10^−7^
+ cancer type	9244	1.035	0.922–1.161	0.558
+ cancer type + age + sex + log mutation burden	9244	1.060	0.940–1.194	0.343
+ cancer type + stage + age + sex + log mutation burden	5848	1.023	0.894–1.169	0.744

**Table 3:** Somatic coding mutations across the ten *APOBEC* genes in pan-cancer TCGA. Recurrent variants are shown with the number of tumors carrying each variant.

Gene	Tumors Mutated	% of Cohort	Cancer Types	Most Recurrent Variant
*APOBEC1*	65	0.67	18	p.K69Nfs*12 (5)
*APOBEC2*	39	0.40	12	p.E185K (3)
*APOBEC3A*	19	0.20	7	none (max 1 tumor)
*APOBEC3B*	56	0.58	17	6 variants (2 tumors each)
*APOBEC3C*	26	0.27	12	p.D149E (2)
*APOBEC3D*	44	0.46	13	p.R375* (3)
*APOBEC3F*	52	0.54	13	p.E38K (3), p.E215K (3)
*APOBEC3G*	50	0.52	18	5 variants (2 tumors each)
*APOBEC3H*	30	0.31	10	p.G67E (2), p.P154L (2)
*APOBEC4*	54	0.56	16	8 variants (2 tumors each)

**Table 4:** ESTIMATE tumor-microenvironment correlations for the ten *APOBEC* family members, showing significant associations, their direction, mean Immune and Stromal correlations, and the strongest individual association. The six immune-associated APOBEC3 enzymes showed stronger Immune than Stromal correlations, whereas APOBEC1 was consistently negative. Overall, 44 cohorts were analyzed, including 33 TCGA types, 6 pooled/split TCGA cohorts (COADREAD, GBMLGG, KIPAN, STES, SKCM-P, SKCM-M), and 5 TARGET pediatric cohorts (ALL, ALL-R, LAML, NB, WT).

Gene	Sig. (/44)	Pos/Neg	Immune r	Stromal r	Strongest Association
*APOBEC1*	7	0/7	−0.05	−0.07	TCGA-PAAD (−0.44)
*APOBEC2*	13	7/6	−0.03	0.03	TARGET-WT (0.32)
*APOBEC3A*	39	39/0	0.38	0.27	TCGA-LAML (0.69)
*APOBEC3B*	17	11/6	0.06	0.05	TCGA-DLBC (0.48)
*APOBEC3C*	39	39/0	0.39	0.32	TCGA-GBMLGG (0.80)
*APOBEC3D*	40	40/0	0.46	0.28	TCGA-THCA (0.67)
*APOBEC3F*	28	27/1	0.25	0.19	TCGA-GBMLGG (0.70)
*APOBEC3G*	40	40/0	0.56	0.39	TCGA-GBMLGG (0.82)
*APOBEC3H*	41	41/0	0.57	0.35	TCGA-CHOL (0.75)
*APOBEC4*	8	5/3	0.03	0.02	TARGET-NB (0.34)

## Data Availability

The data presented in this study were derived from public-domain resources: The Cancer Genome Atlas (TCGA), TARGET and GTEx, accessed via the NCI Genomic Data Commons (https://portal.gdc.cancer.gov) and UCSC Xena (https://xena.ucsc.edu). COSMIC v3.3 reference signatures are available at https://cancer.sanger.ac.uk/cosmic, and the tumor purity and ploidy estimates were obtained from the TCGA PanCanAtlas ABSOLUTE calls. Per-sample CIBERSORT fractions were obtained from the TCGA PanCanAtlas and TIMER2.0 estimates were obtained from http://timer.cistrome.org. The analysis code and the derived data tables that support the findings of this study are deposited at Zenodo (DOI: 10.5281/zenodo.22676594). All web accessed on 10 September 2026.

## Supplementary Materials

The following supporting information can be downloaded at: https://www.mdpi.com/article/10.3390/ijms27198864/s1.

## Abbreviations

The following abbreviations are used in this manuscript:

Term	Definition
AID	Activation-induced deaminase
AJCC	American Joint Committee on Cancer
APOBEC	Apolipoprotein B mRNA-editing enzyme catalytic polypeptide-like
CI	Confidence interval
CIBERSORT	Cell-type Identification By Estimating Relative Subsets Of RNA Transcripts
COSMIC	Catalogue of Somatic Mutations in Cancer
ESTIMATE	Estimation of Stromal and Immune cells in Malignant Tumors
FDR	False discovery rate
GDC	Genomic Data Commons
GTEx	Genotype-Tissue Expression project
HR	Hazard ratio
mRNAsi	mRNA-based stemness index
PD-L1	Programmed death-ligand 1 (CD274)
SBS	Single-base substitution (mutational signature)
TCGA	The Cancer Genome Atlas
TIMER	Tumor Immune Estimation Resource
TME	Tumor microenvironment
