# Supplementary material for: Pan-Cancer APOBEC Mutagenesis, Immune Microenvironment, and Clinical Outcome: A Comprehensive Multi-Omics Analysis of All Ten Family Members Across 9653 TCGA Tumors
Source: Int J Mol Sci. 2026 Oct 5;27(19):8864. doi: 10.3390/ijms27198864 (PMC13664898; doi:10.3390/ijms27198864)
Supplement: Supplementary file 1 [file ijms-27-08864-s001.zip › ijms-4587797-supplementary.pdf]

Supplementary Materials

This file contains Supplementary Figures S1–S13 and Supplementary Tables S1–S18 supporting the main text.

APOBEC enrichment across cancer types

Table S1. APOBEC enrichment and fitted SBS2/SBS13 exposure across the 33 cancer types.

Cancer type	N	% enriched	Median E	Mean % of mutations	% of enriched with fitted signature
BLCA	386	84.5	3.05	50.9	100.0
CESC	269	74.0	2.75	45.7	100.0
HNSC	484	46.9	1.74	23.8	99.6
LUSC	462	44.8	1.57	12.4	87.4
SKCM	422	37.7	1.25	0.7	1.9
LUAD	539	35.3	1.45	10.9	84.7
ESCA	178	23.0	1.18	10.5	97.6
BRCA	985	22.4	1.4	17.5	98.6
CHOL	48	12.5	1.07	6.8	100.0
ACC	80	7.5	1.11	5.7	83.3
UCEC	515	5.6	0.49	4.5	100.0
OV	383	5.2	1.11	4.0	95.0
THCA	461	4.6	0.72	8.1	100.0
UCS	56	3.6	0.71	5.3	100.0
STAD	404	3.2	0.77	3.2	100.0
KIRP	261	3.1	1.12	2.6	100.0
PAAD	167	3.0	0.68	3.1	100.0
SARC	222	2.7	1.06	3.2	33.3
DLBC	39	2.6	0.76	4.2	100.0
THYM	116	1.7	0.0	1.7	100.0
KIRC	351	1.4	1.09	2.1	80.0
COAD	413	0.7	0.51	1.2	66.7
LIHC	339	0.6	1.03	1.0	100.0
LGG	487	0.4	0.52	1.0	100.0

GBM	366	0.3	0.53	1.3	100.0
KICH	60	0.0	0.62	2.1	-
LAML	112	0.0	0.0	1.0	-
MESO	73	0.0	0.85	3.0	-
PCPG	168	0.0	0.0	3.5	-
PRAD	456	0.0	0.66	2.1	-
READ	141	0.0	0.61	1.0	-
TGCT	134	0.0	0.71	3.5	-
UVM	76	0.0	0.02	3.4	-

APOBEC and clinical outcome

Figure S1. APOBEC enrichment and clinical outcome (unadjusted). (a) Enrichment by AJCC stage. (b) Kaplan–Meier overall survival, enriched versus non-enriched.

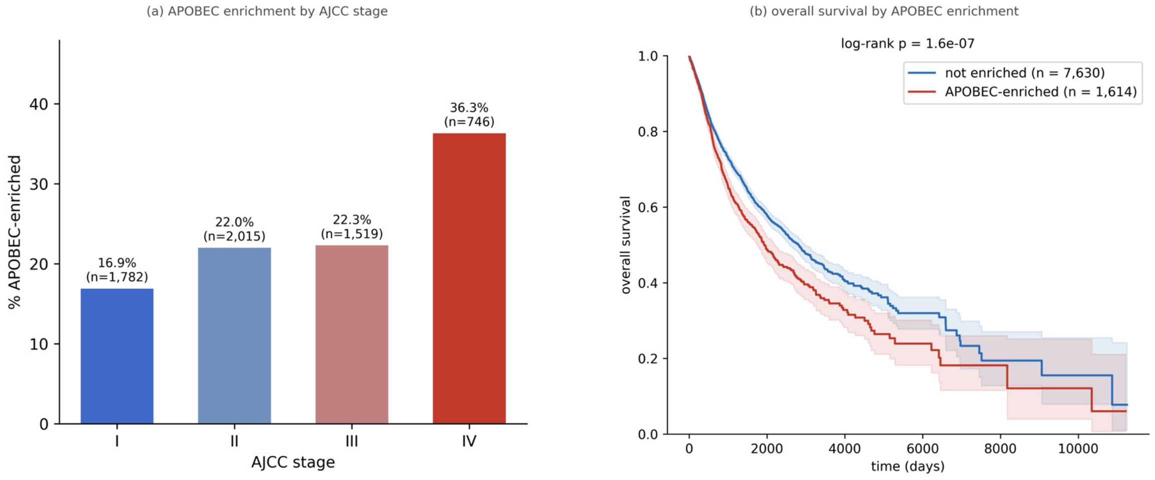

Table S2. Cancer-type composition of each AJCC stage.

**cancer**	**I**	**II**	**III**	**IV**
ACC	0.4	1.9	1.3	2.0
BLCA	0.2	6.2	8.6	16.9
BRCA	9.1	28.1	14.1	2.5
CHOL	1.0	0.7	0.3	1.2

COAD	3.9	8.2	7.0	7.9
ESCA	0.9	3.7	3.6	1.2
HNSC	1.2	3.3	4.9	33.9
KICH	1.1	1.2	0.8	0.5
KIRC	10.6	2.0	5.0	5.9
KIRP	8.6	1.0	3.0	2.0
LIHC	8.9	3.9	5.0	0.7
LUAD	15.2	5.6	5.0	3.4
LUSC	12.3	7.5	5.1	0.9
MESO	0.5	0.7	2.5	1.6
PAAD	1.0	6.8	0.2	0.8
READ	1.6	2.0	2.6	2.9
SKCM	3.6	6.4	10.3	2.7
STAD	3.0	6.1	10.7	5.4
TGCT	2.4	0.5	0.9	0.0
THCA	14.4	2.4	6.7	7.0
UVM	0.0	1.8	2.3	0.5

Table S3. Cox models of APOBEC enrichment against overall survival, with stage added.

**model**	**n**	**deaths**	**HR**	**CI_low**	**CI_high**	**p**	**concordance**
1. unadjusted	5848	1774	1.414	1.275	1.567	4.3e-11	0.529
2. + cancer type	5848	1774	1.022	0.9	1.161	0.739	0.498
3. + cancer type + stage	5848	1774	0.996	0.877	1.132	0.954	0.631
4. + cancer type + stage + age + sex	5848	1774	0.965	0.849	1.097	0.588	0.661
5. + cancer type + stage + age + sex + log burden	5848	1774	1.023	0.894	1.169	0.744	0.663

Table S4. Functional forms for APOBEC burden in overall survival, by adjustment block.

**Block**	**Model**	**df**	**AIC improvement**	**LRT p**	**C-index**
A. unadjusted	binary: Roberts enrichment (q<0.05)	1	+24.12	3.2e-07	0.517
A. unadjusted	binary: any APOBEC mutation (>0)	1	+19.95	2.8e-06	0.519
A. unadjusted	binary: median split	1	+19.95	2.8e-06	0.519
A. unadjusted	binary: top quartile	1	+42.39	2.69e-11	0.527
A. unadjusted	categorical: quartiles	3	+131.15	1.56e-29	0.555
A. unadjusted	continuous: raw burden (per SD)	1	-1.99	0.929	0.523
A. unadjusted	continuous: sqrt burden (per SD)	1	+10.04	0.000521	0.523
A. unadjusted	continuous: log1p burden (per SD)	1	+29.00	2.58e-08	0.523
A. unadjusted	spline 3 knots (log)	2	+37.81	8.34e-10	0.525
A. unadjusted	spline 4 knots (log)	3	+52.15	1.46e-12	0.525
A. unadjusted	spline 5 knots (log)	4	+54.25	9.75e-13	0.539
B. + cancer type	binary: Roberts enrichment (q<0.05)	1	-1.66	0.559	0.500
B. + cancer type	binary: any APOBEC mutation (>0)	1	+2.74	0.0294	0.508
B. + cancer type	binary: median split	1	+2.74	0.0294	0.508
B. + cancer type	binary: top quartile	1	+7.32	0.00226	0.511
B. + cancer type	categorical: quartiles	3	+10.59	0.000857	0.519
B. + cancer type	continuous: raw burden (per SD)	1	+9.22	0.000808	0.497
B. + cancer type	continuous: sqrt burden (per SD)	1	-0.61	0.239	0.497
B. + cancer type	continuous: log1p burden (per SD)	1	-0.45	0.214	0.503
B. + cancer type	spline 3 knots (log)	2	+17.73	1.92e-05	0.519
B. + cancer type	spline 4 knots (log)	3	+18.23	2.24e-05	0.518
B. + cancer type	spline 5 knots (log)	4	+16.94	5.17e-05	0.519
C. + age, sex	binary: Roberts enrichment (q<0.05)	1	-1.99	0.92	0.593
C. + age, sex	binary: any APOBEC mutation (>0)	1	+0.84	0.0917	0.594
C. + age, sex	binary: median split	1	+0.84	0.0917	0.594
C. + age, sex	binary: top quartile	1	+3.45	0.0195	0.595
C. + age, sex	categorical: quartiles	3	+6.97	0.00469	0.596
C. + age, sex	continuous: raw burden (per SD)	1	+13.77	7.15e-05	0.596

C. + age, sex	continuous: sqrt burden (per SD)	1	+2.30	0.038	0.594
C. + age, sex	continuous: log1p burden (per SD)	1	-1.89	0.74	0.593
C. + age, sex	spline 3 knots (log)	2	+18.41	1.36e-05	0.599
C. + age, sex	spline 4 knots (log)	3	+17.69	2.9e-05	0.599
C. + age, sex	spline 5 knots (log)	4	+15.87	8.47e-05	0.600
D. + stage (n = 5,848)	binary: Roberts enrichment (q<0.05)	1	-1.71	0.588	0.661
D. + stage (n = 5,848)	binary: any APOBEC mutation (>0)	1	+2.67	0.0307	0.661
D. + stage (n = 5,848)	binary: median split	1	+2.67	0.0307	0.661
D. + stage (n = 5,848)	binary: top quartile	1	+4.75	0.00939	0.661
D. + stage (n = 5,848)	categorical: quartiles	3	+6.58	0.00564	0.662
D. + stage (n = 5,848)	continuous: raw burden (per SD)	1	+8.95	0.000936	0.663
D. + stage (n = 5,848)	continuous: sqrt burden (per SD)	1	+0.33	0.127	0.661
D. + stage (n = 5,848)	continuous: log1p burden (per SD)	1	-1.57	0.513	0.660
D. + stage (n = 5,848)	spline 3 knots (log)	2	+16.19	4.12e-05	0.664
D. + stage (n = 5,848)	spline 4 knots (log)	3	+14.28	0.000149	0.664
D. + stage (n = 5,848)	spline 5 knots (log)	4	+12.29	0.000438	0.664

Somatic mutation landscape of the APOBEC genes

Figure S2. Somatic coding mutations in the ten APOBEC family genes (lollipop plots).

APOBEC1

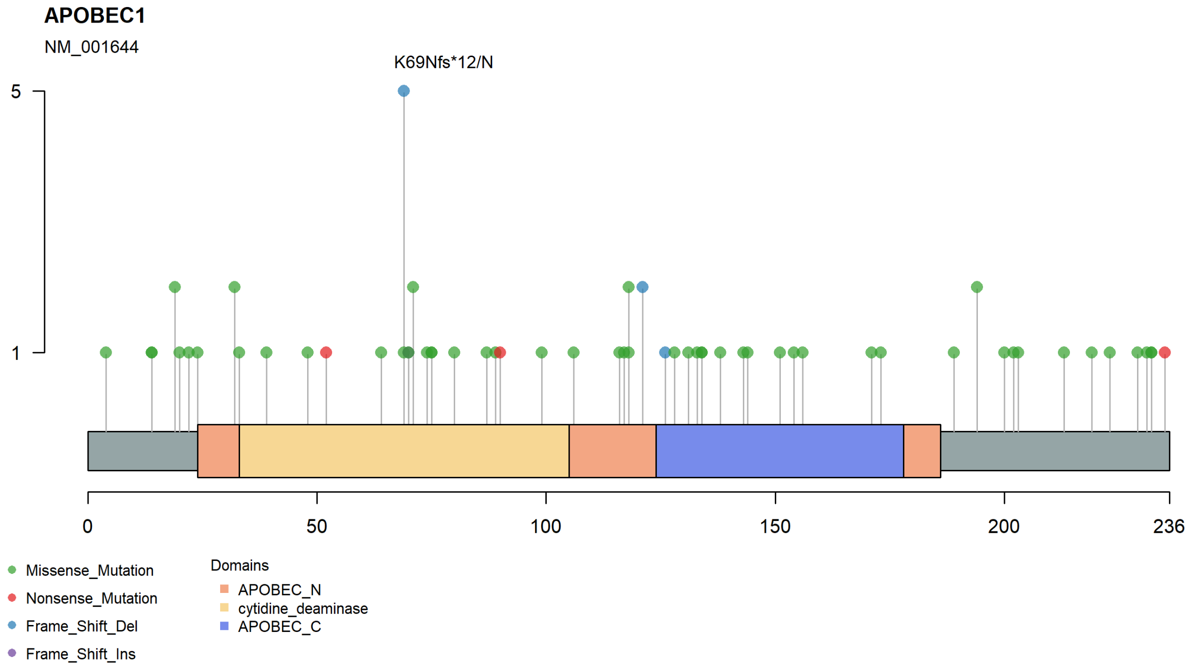

APOBEC2

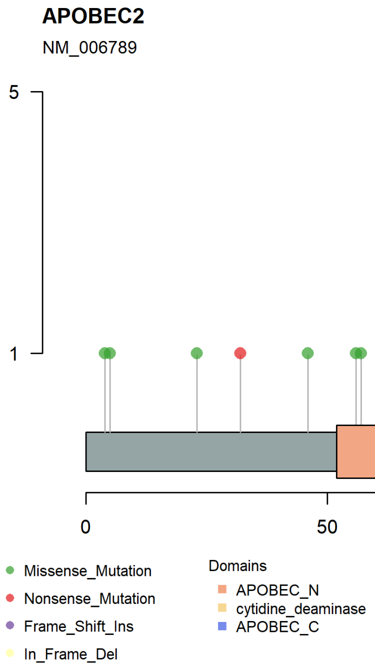

APOBEC3A

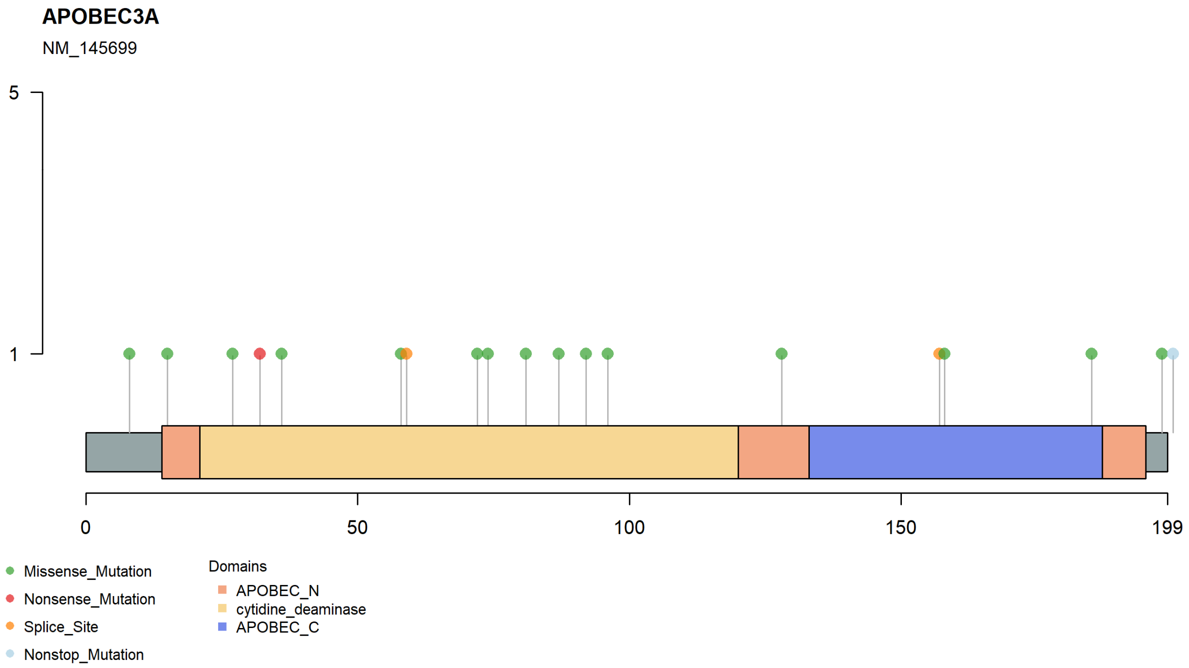

APOBEC3B

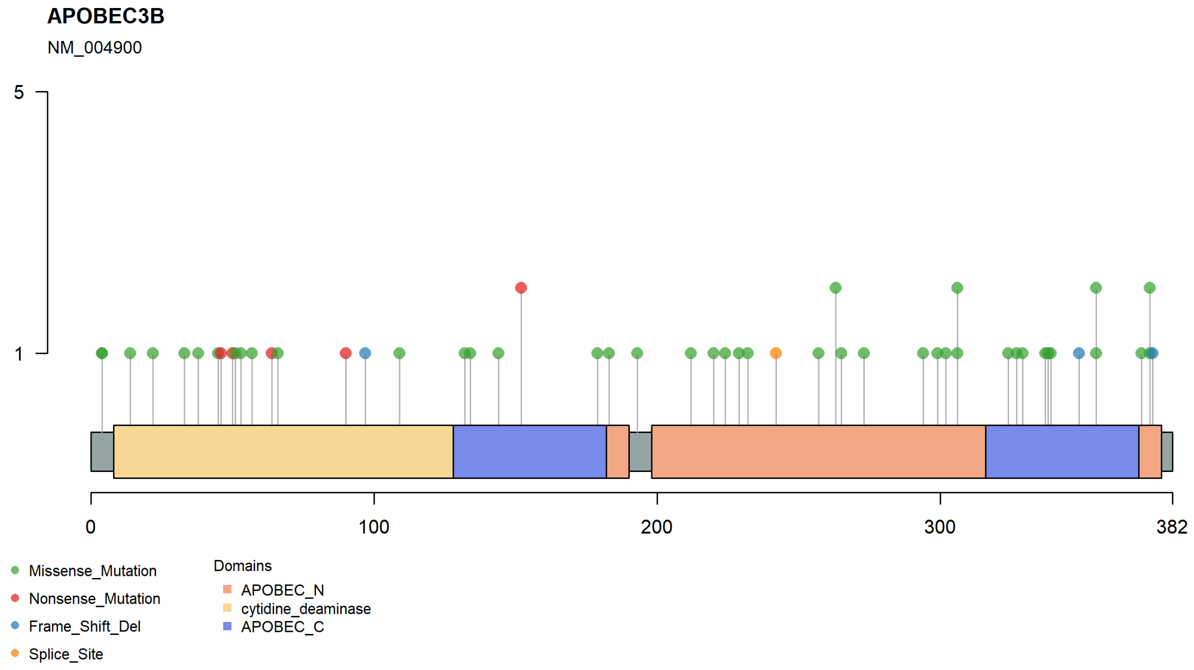

APOBEC3C

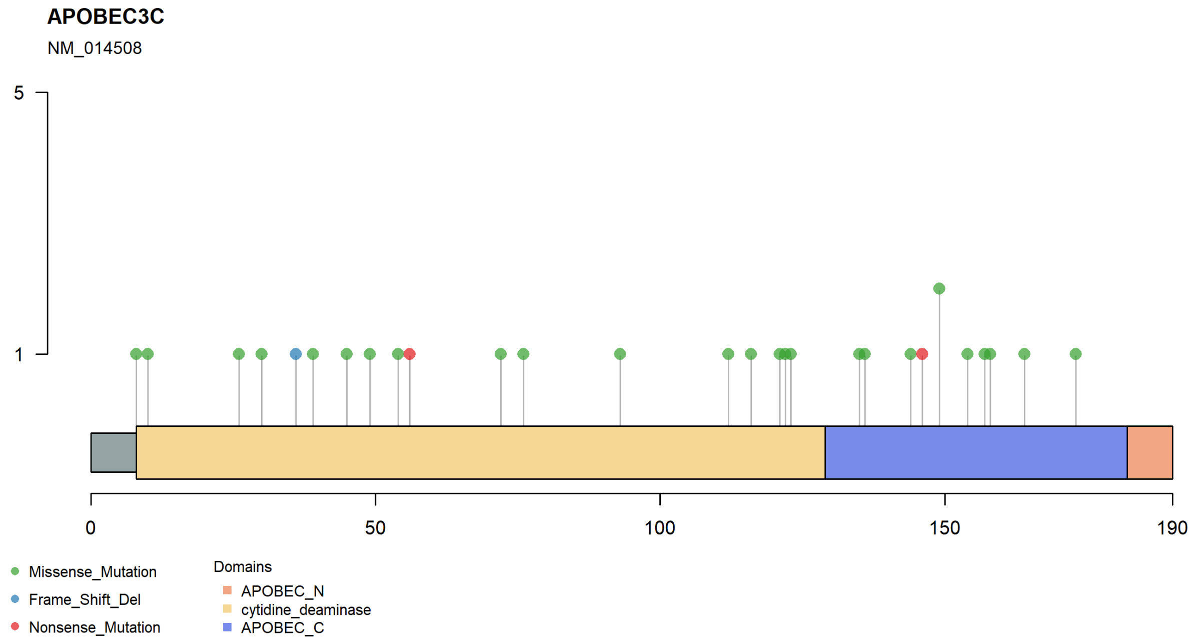

APOBEC3D

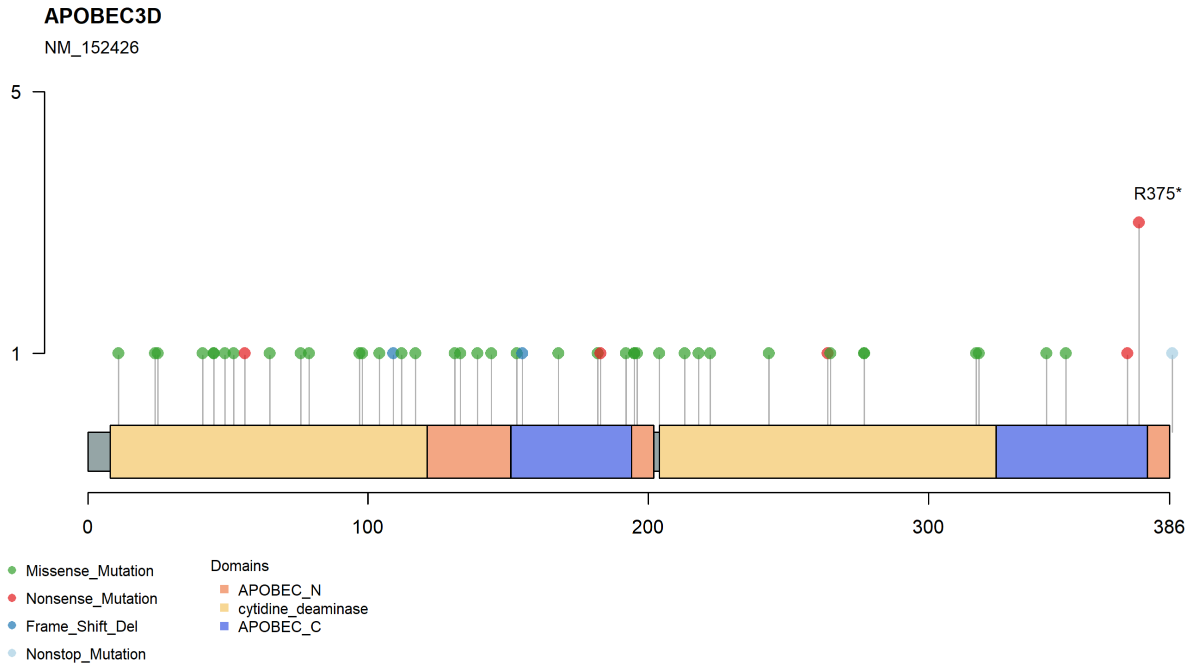

APOBEC3F

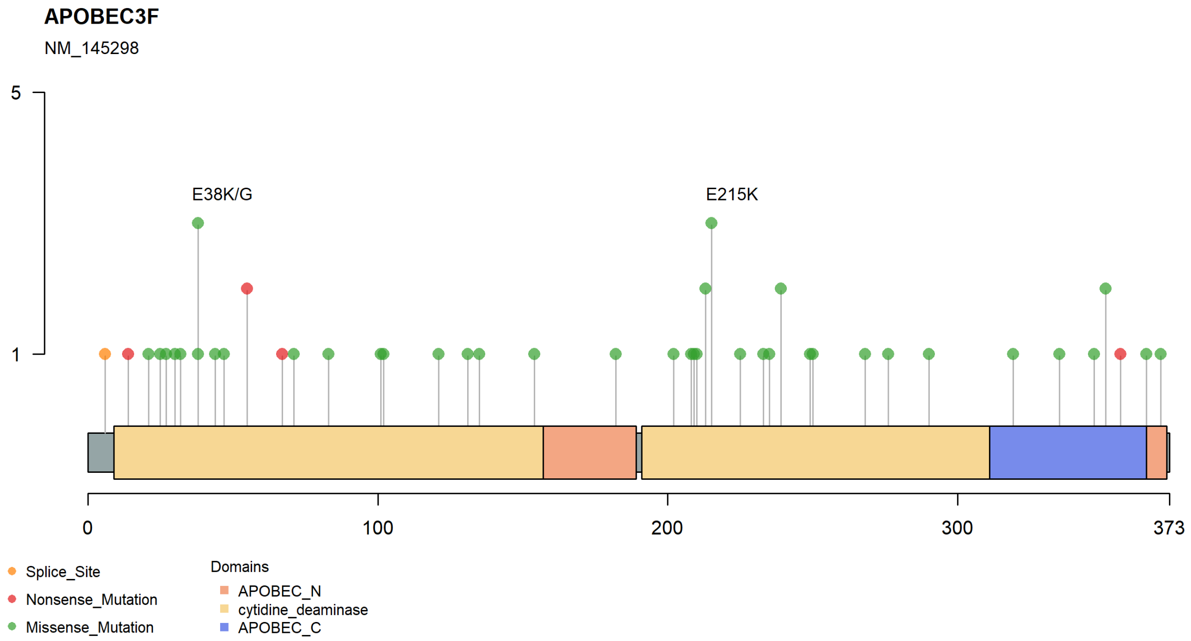

APOBEC3G

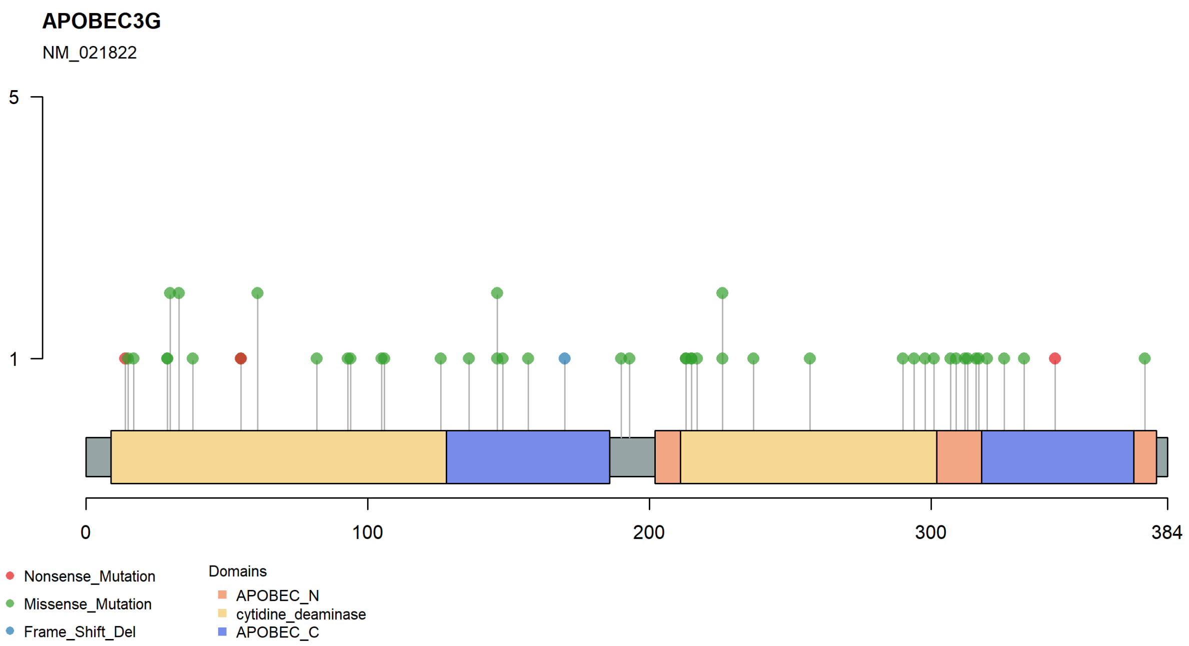

APOBEC3H

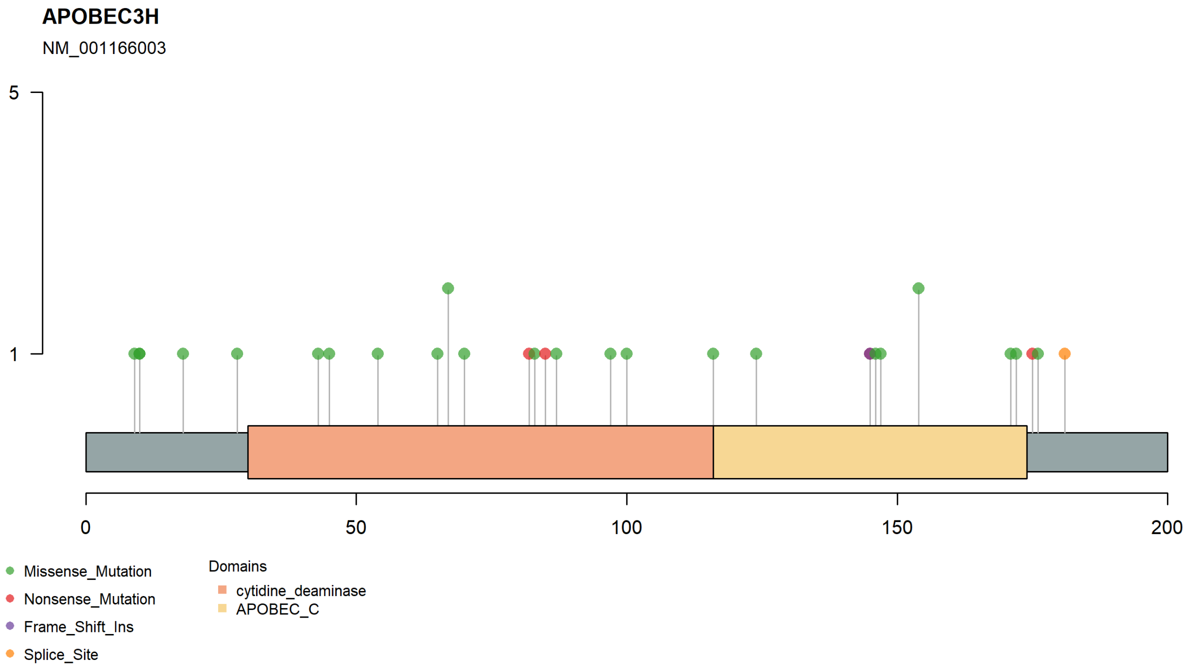

APOBEC4

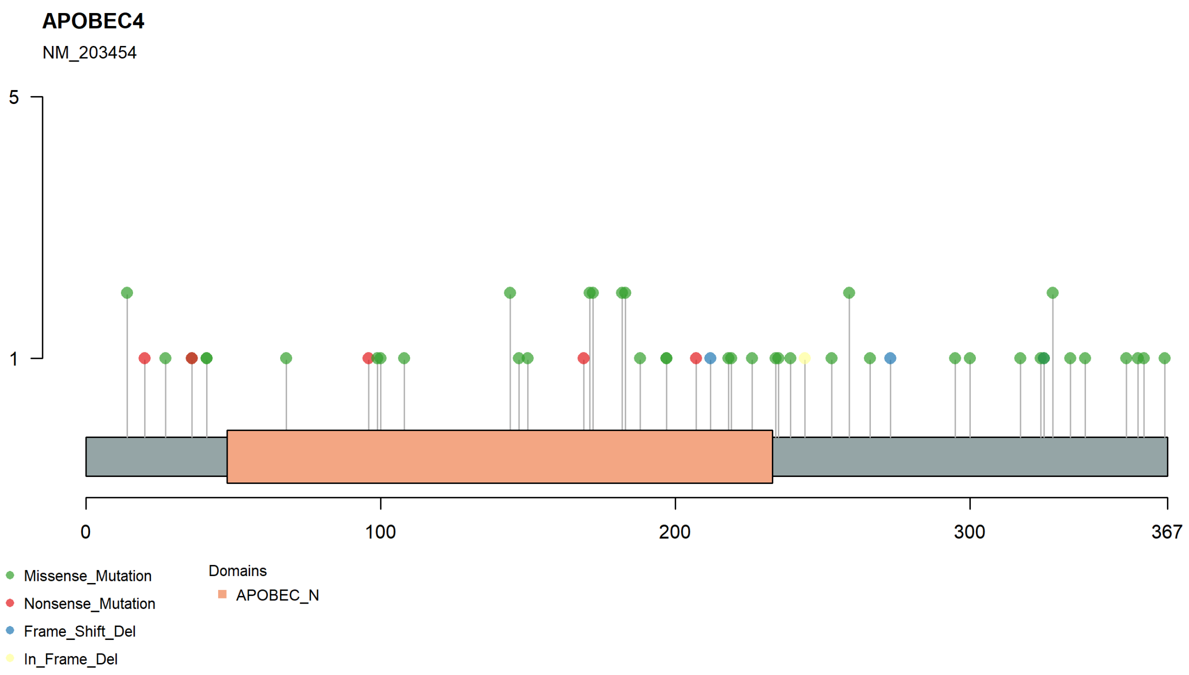
APOBEC4
NM_203454
5
1
0
100
200
300
367
Missense_Mutation
Nonsense_Mutation
Frame_Shift_Del
In_Frame_Del
Domains
APOBEC_N

Figure S3. Per-cancer distribution of somatic coding mutations in the ten APOBEC family genes.

APOBEC1

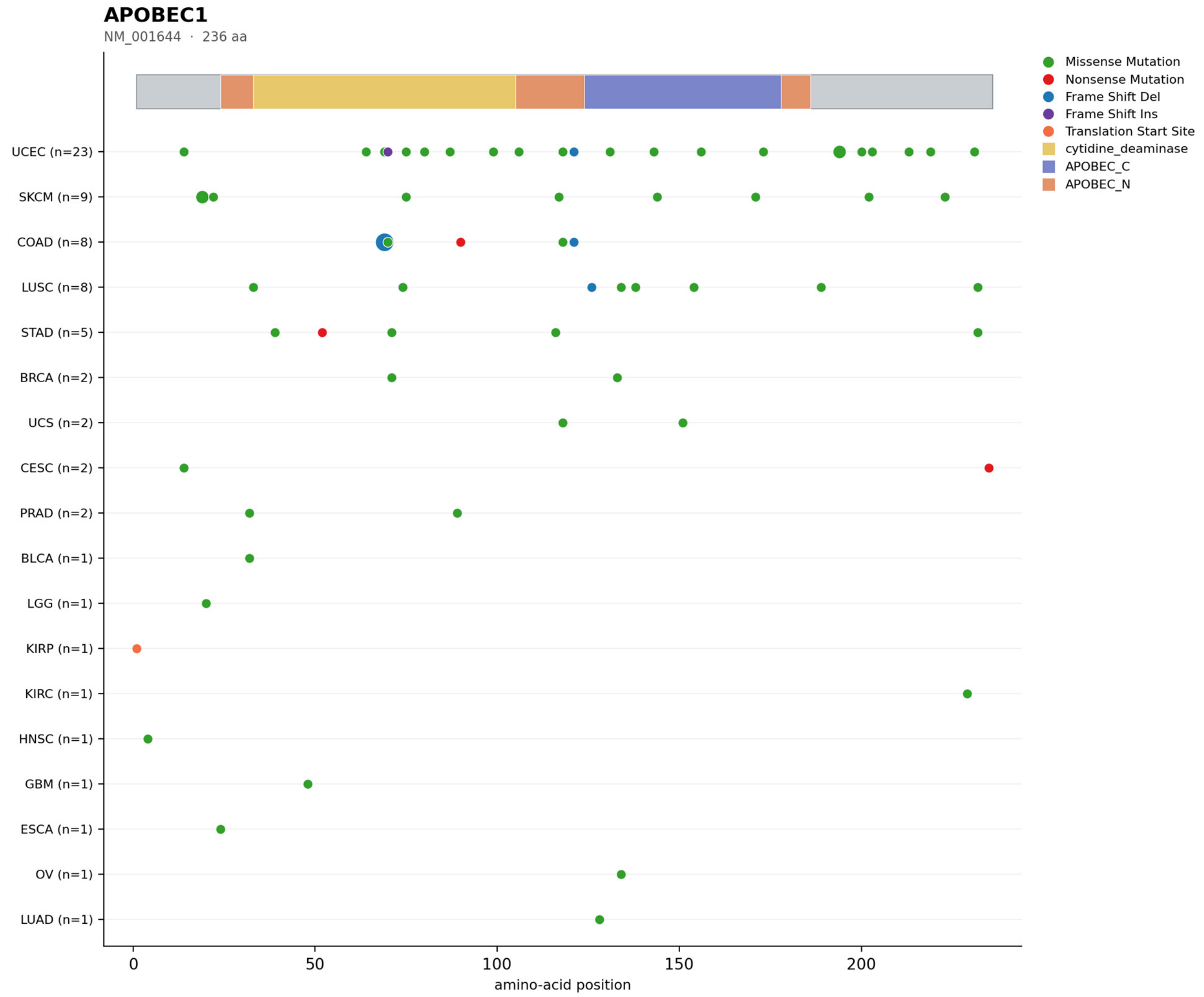

APOBEC2

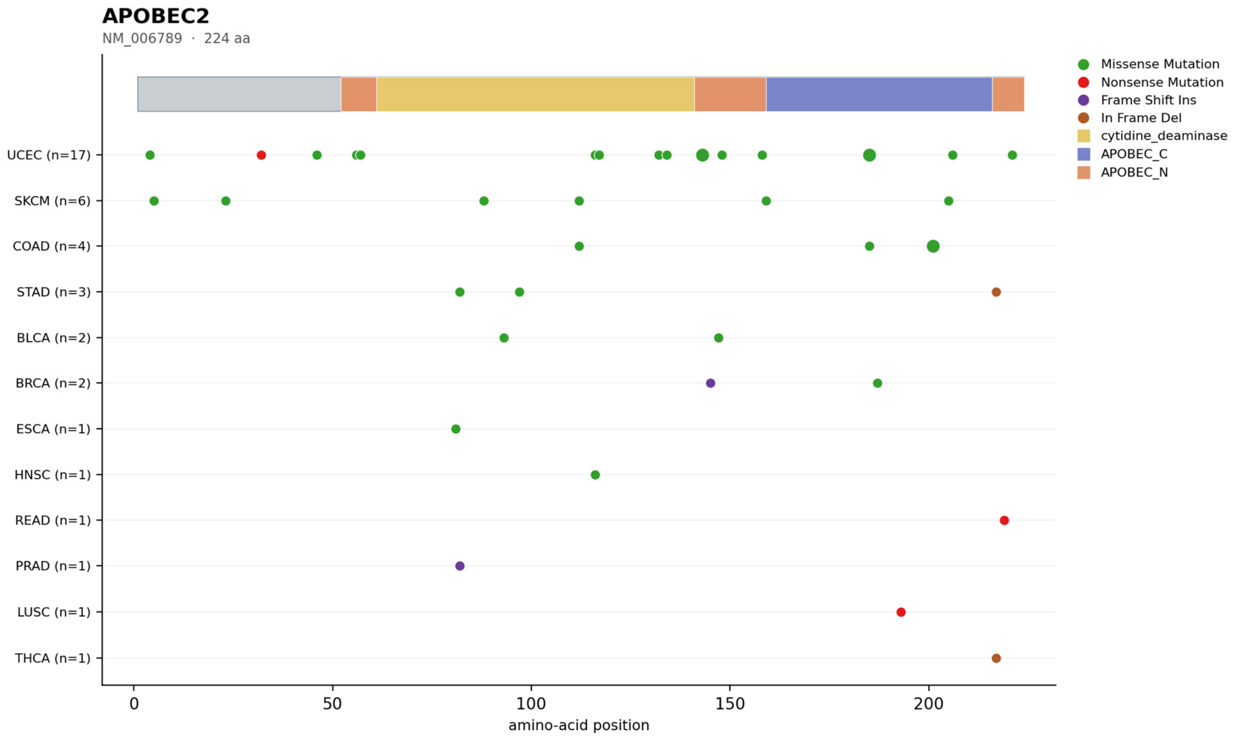

APOBEC3A

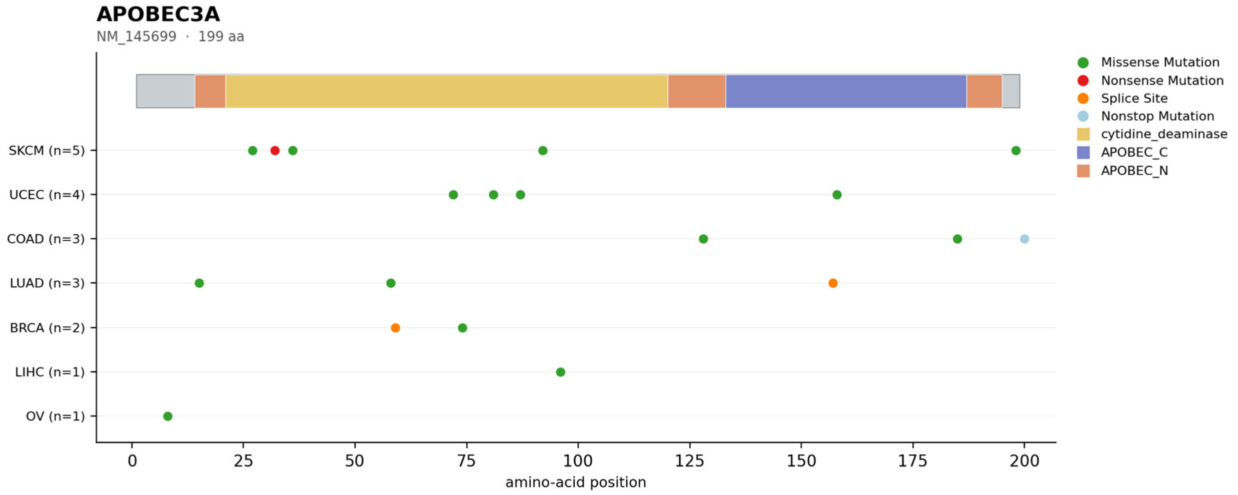

APOBEC3B

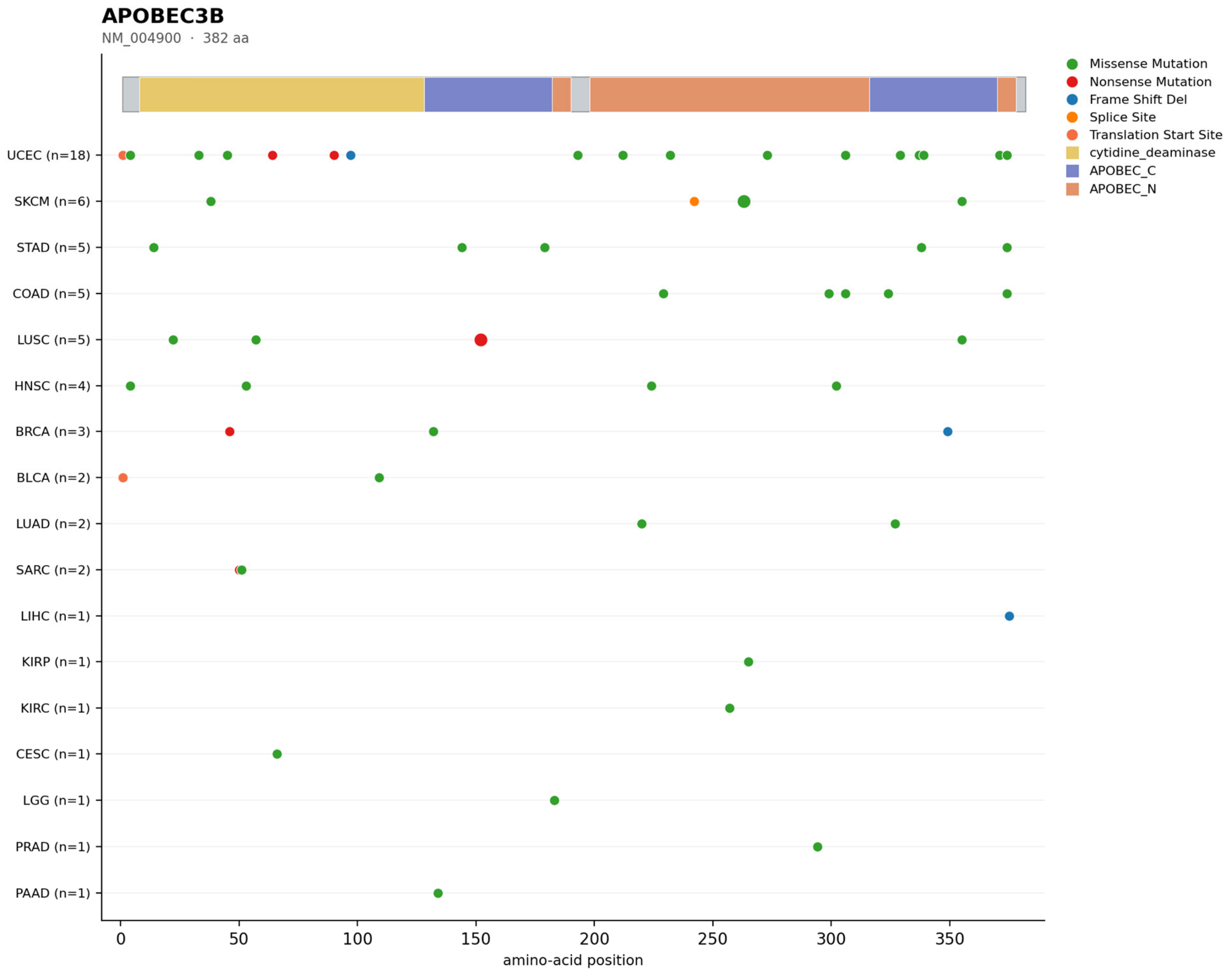

APOBEC3C

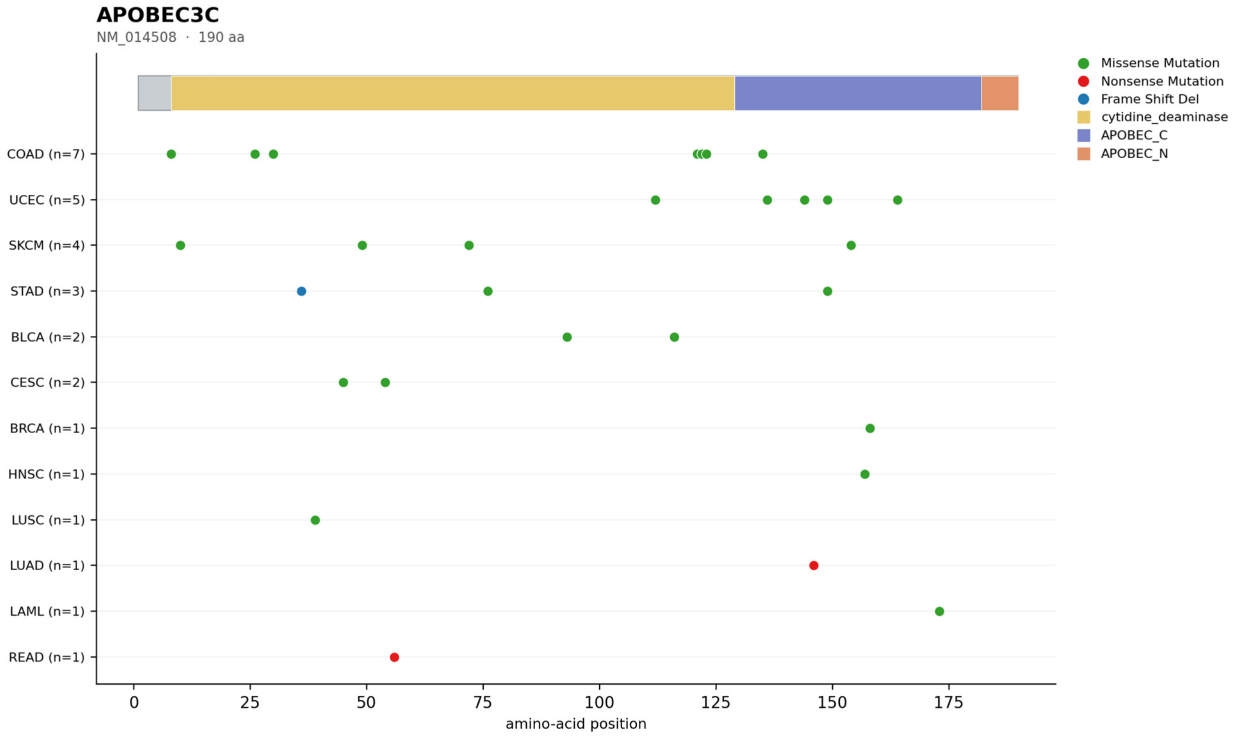

APOBEC3D

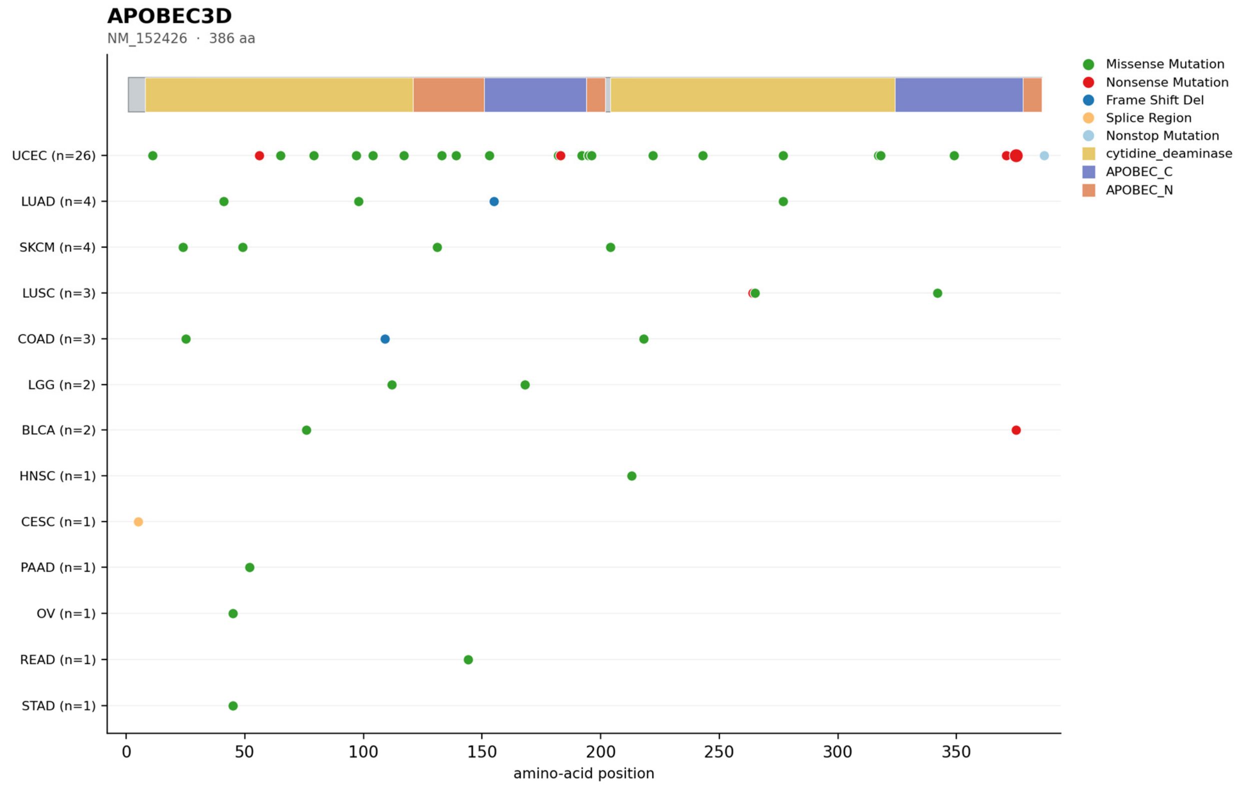

APOBEC3F

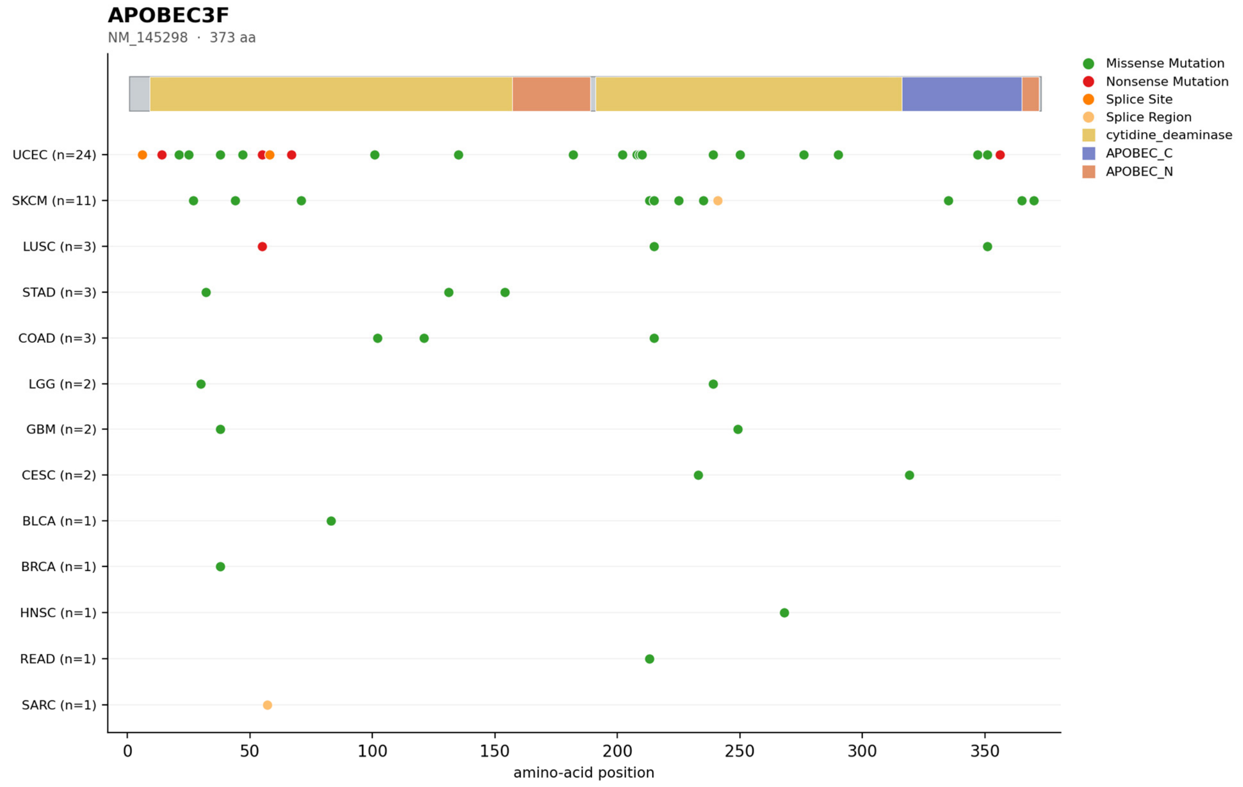

APOBEC3G

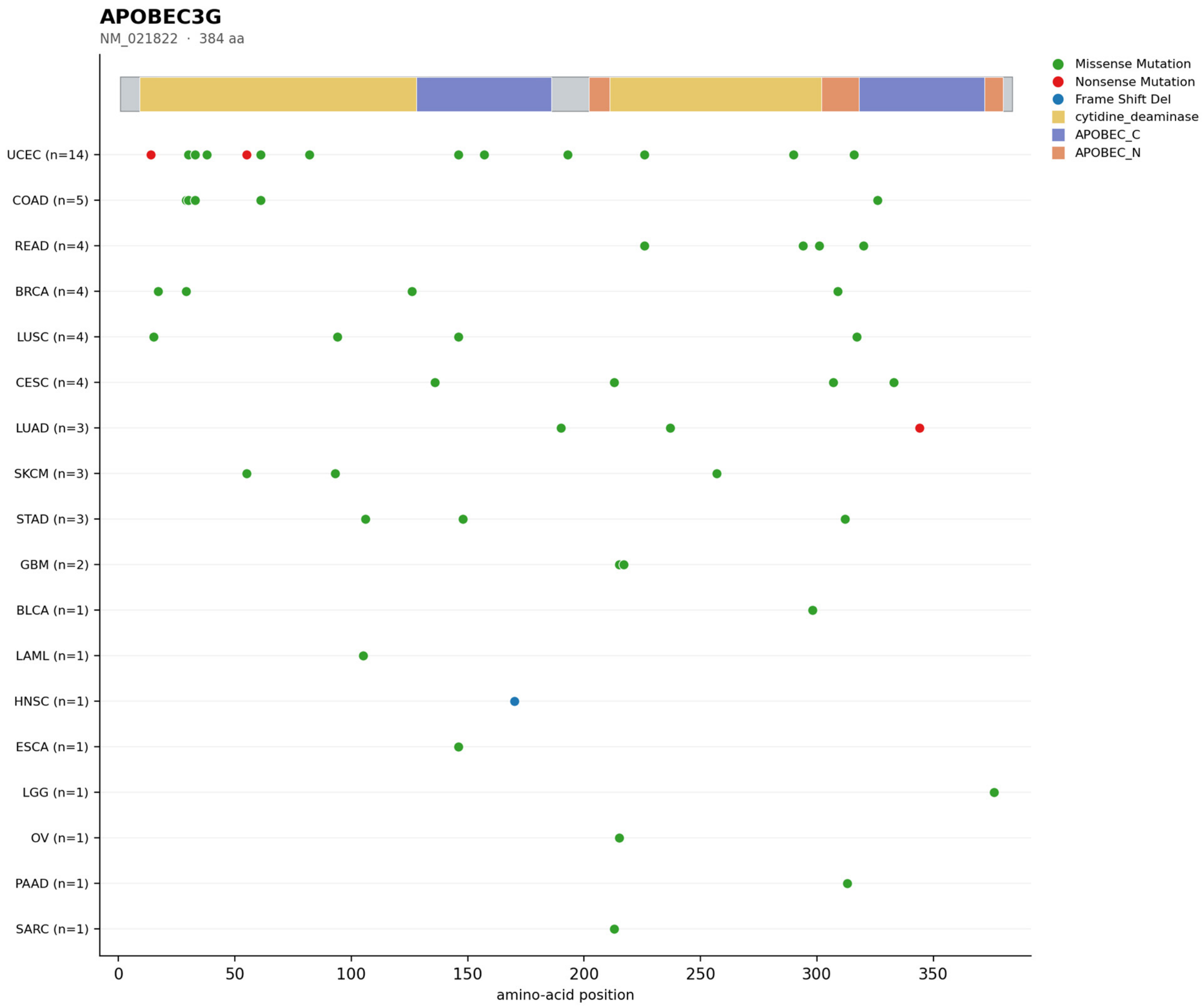

APOBEC3H

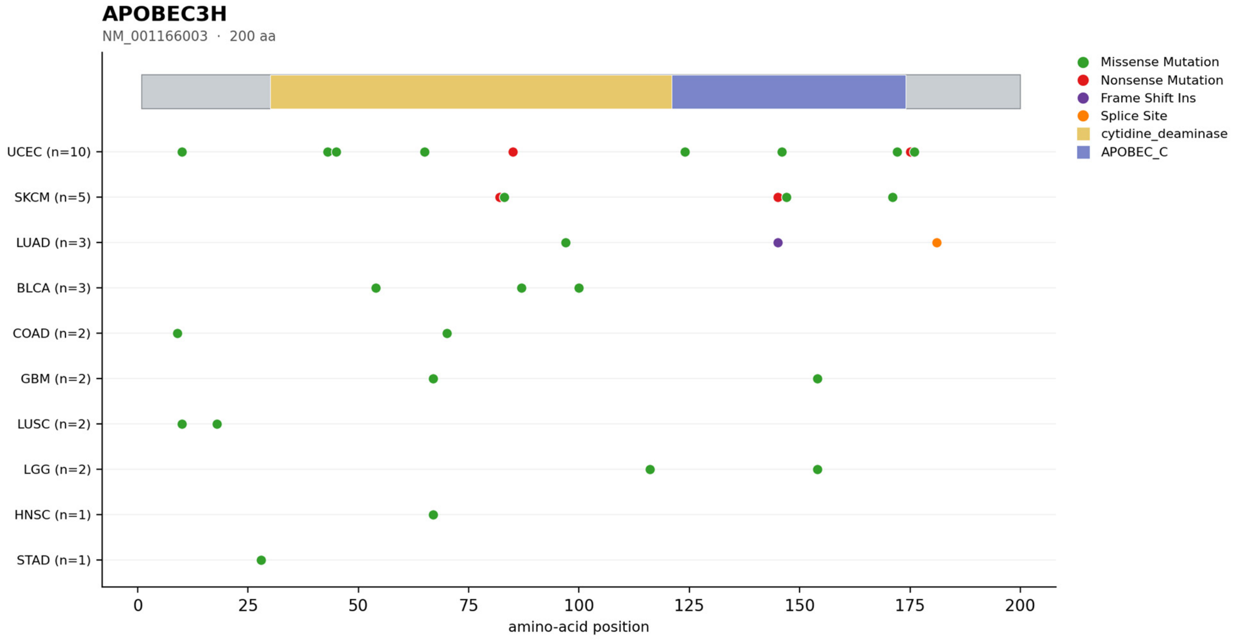

APOBEC4

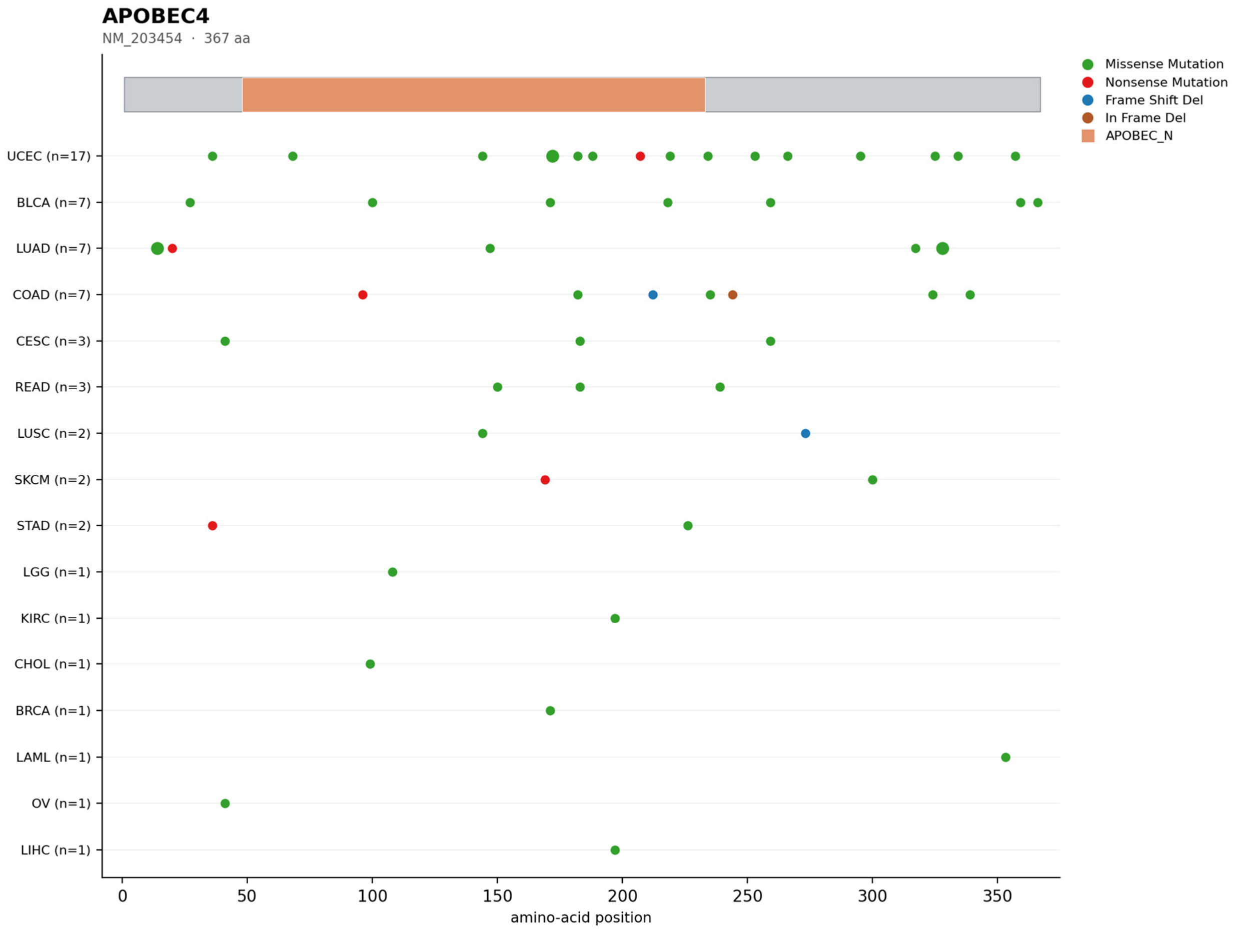

Table S5. dN/dS analysis of the ten APOBEC genes.

Gene	Synonymous	Missense	Nonsense	dN/dS (missense)	q-value
APOBEC1	13	33	1	1.14	0.81
APOBEC2	10	16	2	0.87	0.96
APOBEC3A	11	9	1	0.43	0.95
APOBEC3B	9	32	5	1.62	0.96
APOBEC3C	8	16	2	1.02	1.00
APOBEC3D	19	20	3	0.51	0.93
APOBEC3F	9	21	2	1.05	1.00
APOBEC3G	10	31	1	1.31	0.96
APOBEC3H	5	16	1	1.62	0.97
APOBEC4	5	32	2	2.33	0.89

Tumor-versus-normal expression

Figure S4. Tumor-versus-normal expression of the ten APOBEC family genes (split violins).
TCGA–GTEx. Tumor (red) and normal (blue) distributions are shown per cancer type (*p<0.05, **p<0.01, ***p<0.001, ****p<0.0001). Expression, $\log_2$(TPM + 1); nominal p values; sample numbers in Table S16.

APOBEC1

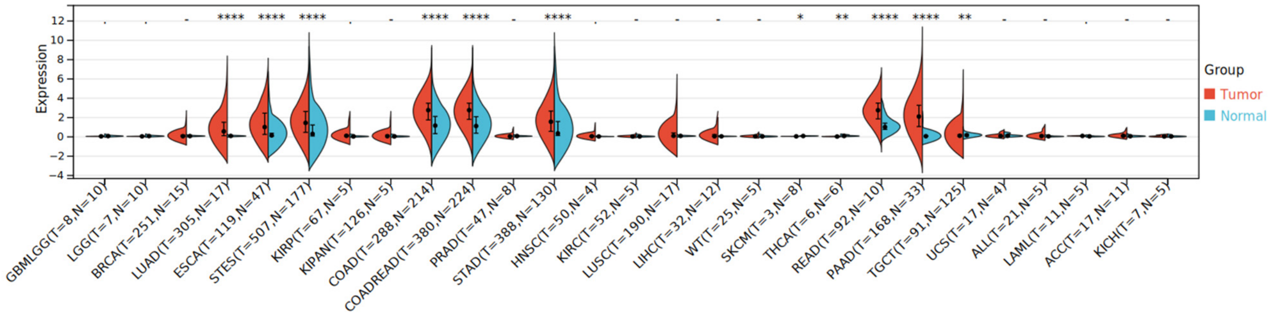

APOBEC2

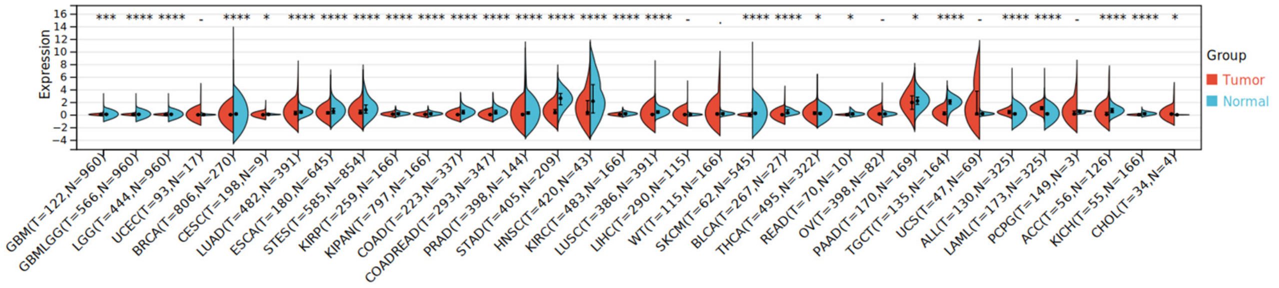

APOBEC3A

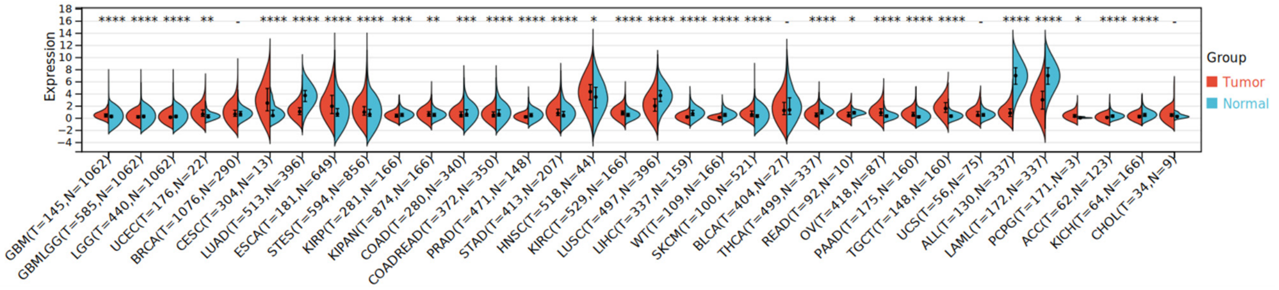

APOBEC3B

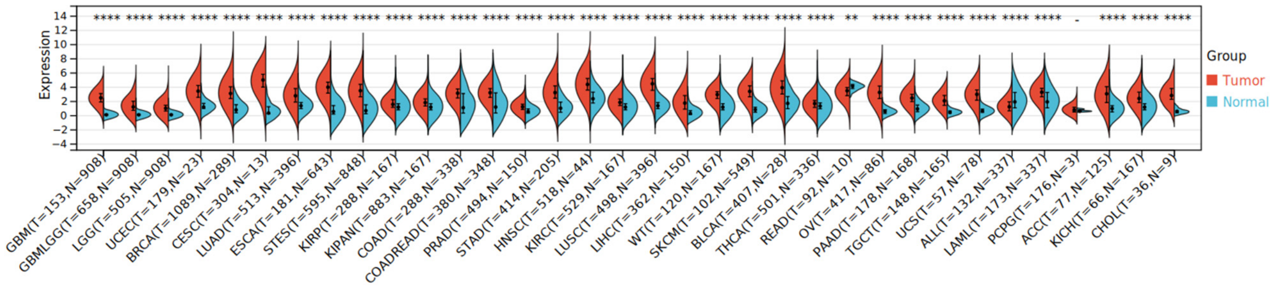

APOBEC3C

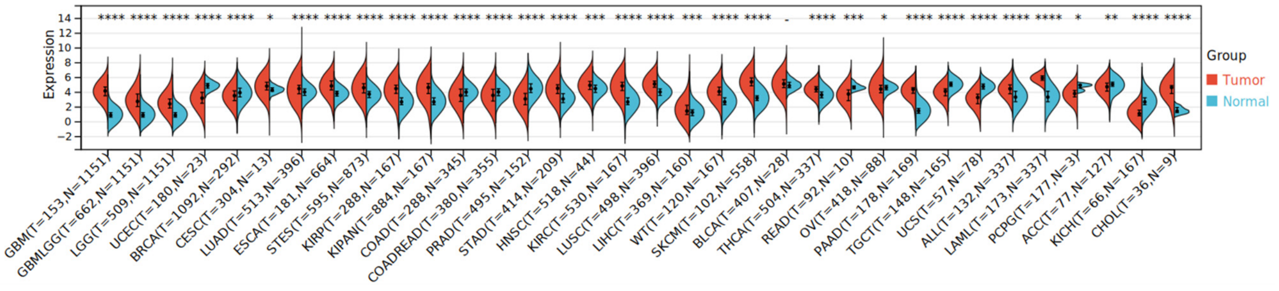

APOBEC3D

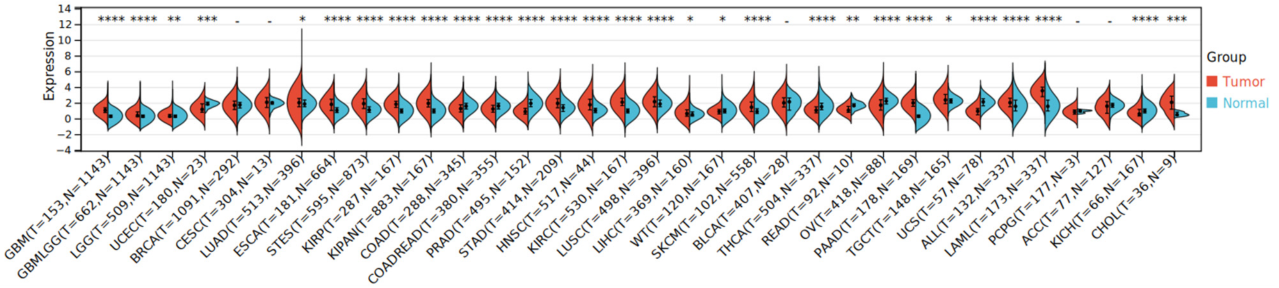

APOBEC3F

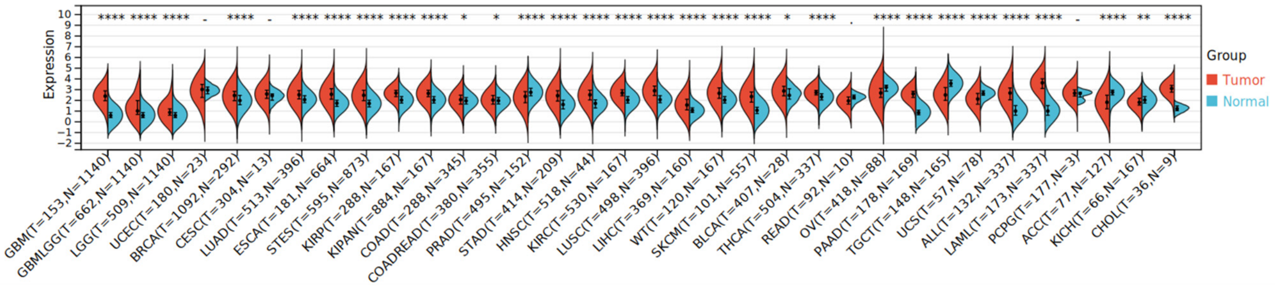

APOBEC3G

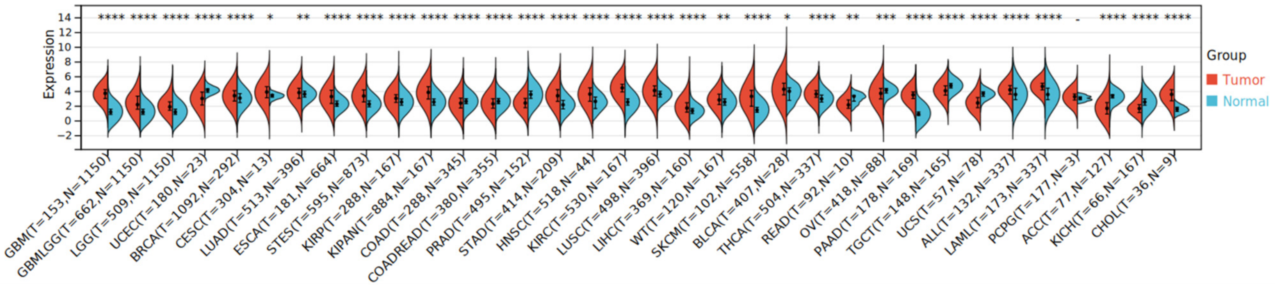

APOBEC3H

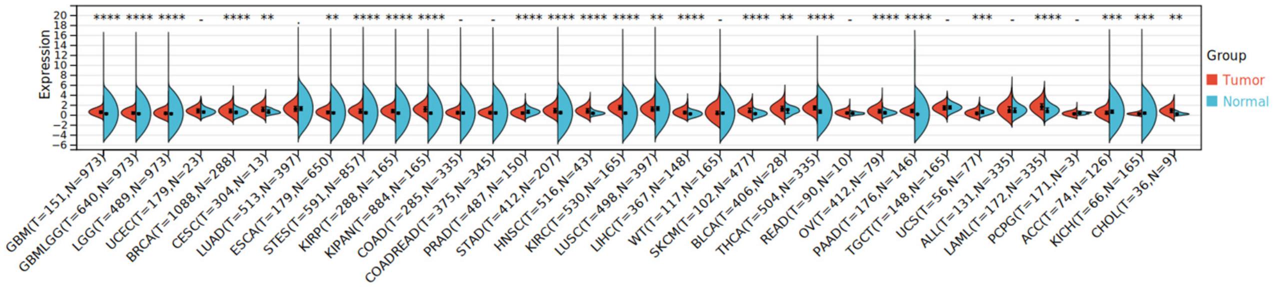

APOBEC4

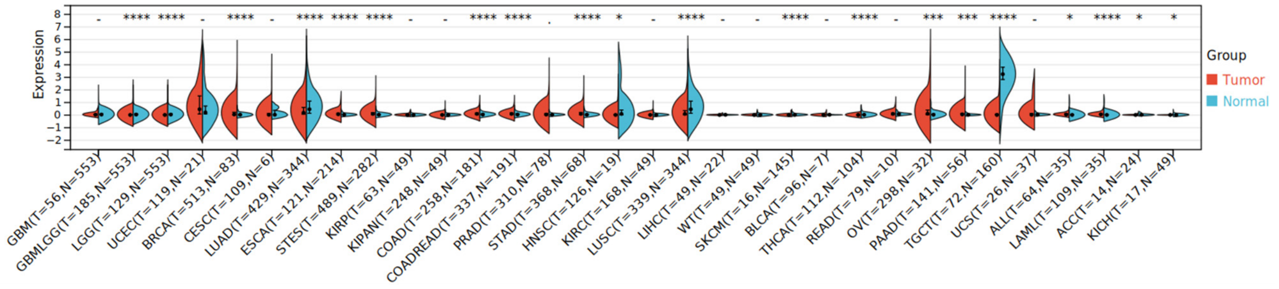

APOBEC expression and the immune microenvironment

Figure S5. Correlation of APOBEC gene expression with ESTIMATE scores, per gene. Significance, nominal $p < 0.05$ (see Table S14).

APOBEC1

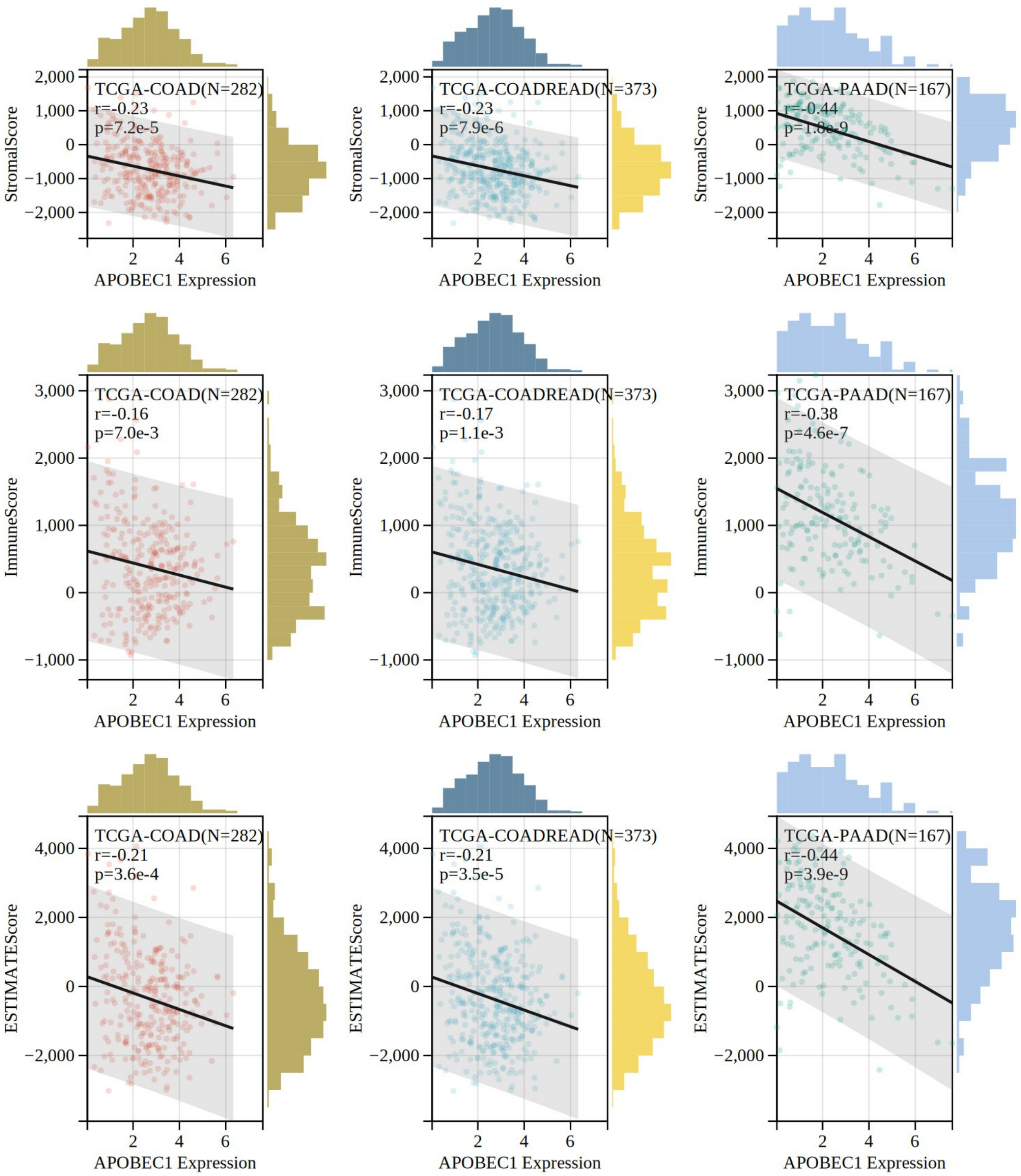
TCGA-COAD(N=282)
r=-0.23
p=7.2e-5
StromalScore
APOBEC1 Expression
TCGA-COADREAD(N=373)
r=-0.23
p=7.9e-6
StromalScore
APOBEC1 Expression
TCGA-PAAD(N=167)
r=-0.44
p=1.8e-9
StromalScore
APOBEC1 Expression
TCGA-COAD(N=282)
r=-0.16
p=7.0e-3
ImmuneScore
APOBEC1 Expression
TCGA-COADREAD(N=373)
r=-0.17
p=1.1e-3
ImmuneScore
APOBEC1 Expression
TCGA-PAAD(N=167)
r=-0.38
p=4.6e-7
ImmuneScore
APOBEC1 Expression
TCGA-COAD(N=282)
r=-0.21
p=3.6e-4
ESTIMATEScore
APOBEC1 Expression
TCGA-COADREAD(N=373)
r=-0.21
p=3.5e-5
ESTIMATEScore
APOBEC1 Expression
TCGA-PAAD(N=167)
r=-0.44
p=3.9e-9
ESTIMATEScore
APOBEC1 Expression

APOBEC2

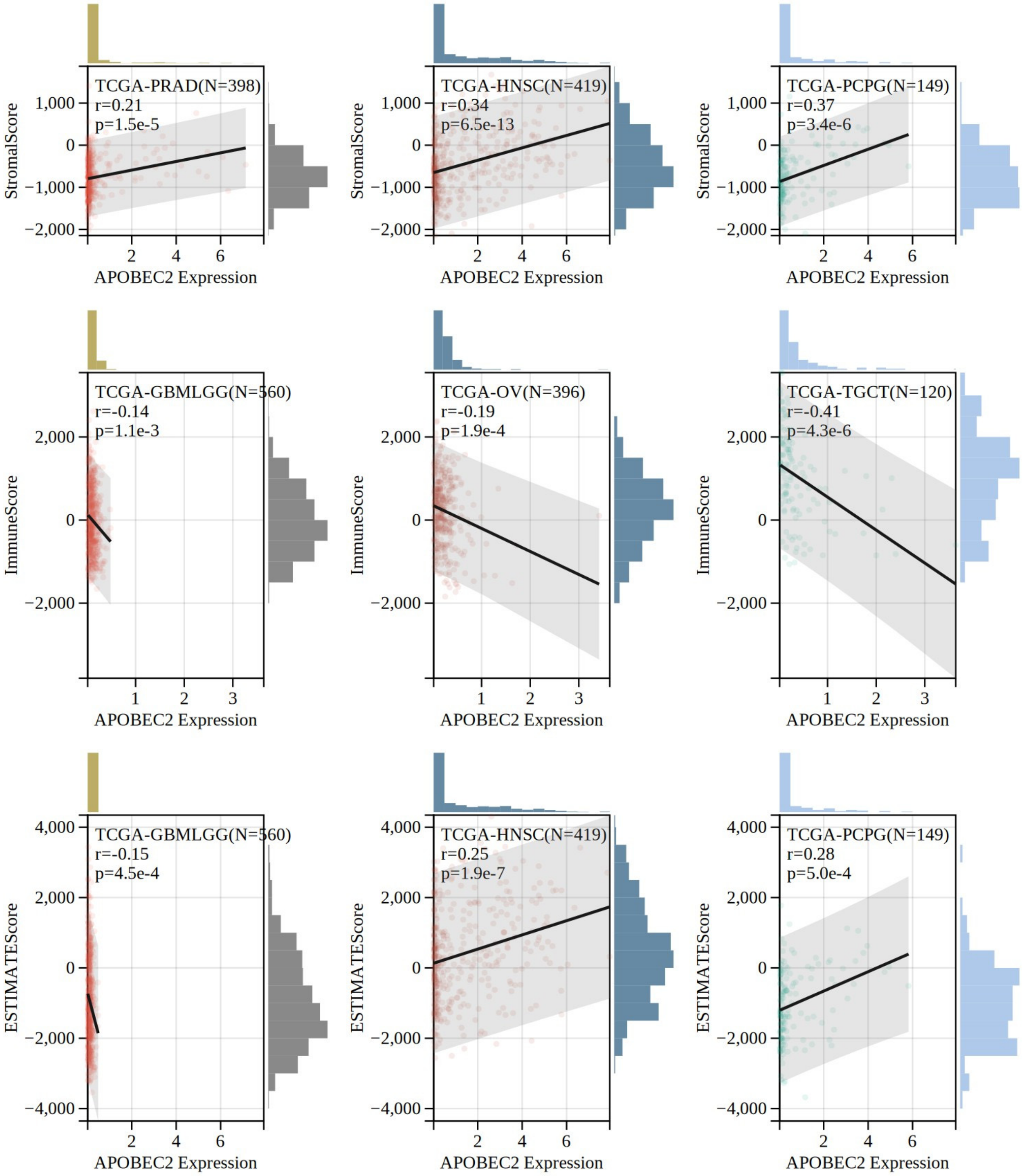
TCGA-PRAD(N=398)
r=0.21
p=1.5e-5
TCGA-HNSC(N=419)
r=0.34
p=6.5e-13
TCGA-PCPG(N=149)
r=0.37
p=3.4e-6
StromalScore
TCGA-GBMLGG(N=560)
r=-0.14
p=1.1e-3
TCGA-OV(N=396)
r=-0.19
p=1.9e-4
TCGA-TGCT(N=120)
r=-0.41
p=4.3e-6
ImmuneScore
TCGA-GBMLGG(N=560)
r=-0.15
p=4.5e-4
TCGA-HNSC(N=419)
r=0.25
p=1.9e-7
TCGA-PCPG(N=149)
r=0.28
p=5.0e-4
ESTIMATEScore
APOBEC2 Expression

APOBEC3A

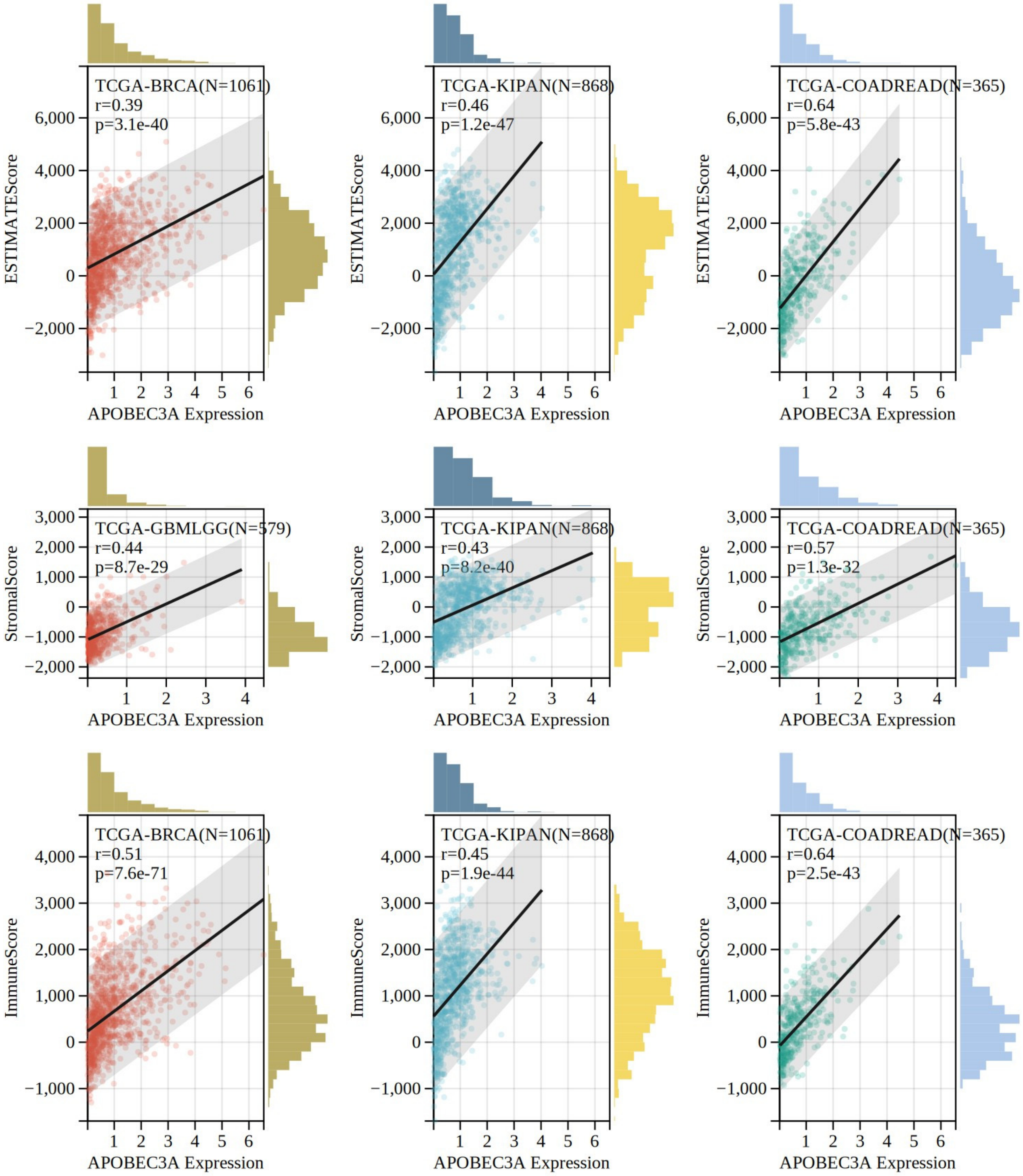
TCGA-BRCA(N=1061)
r=0.39
p=3.1e-40
ESTIMATEScore
APOBEC3A Expression
TCGA-KIPAN(N=868)
r=0.46
p=1.2e-47
TCGA-COADREAD(N=365)
r=0.64
p=5.8e-43
TCGA-GBMLGG(N=579)
r=0.44
p=8.7e-29
StromalScore
TCGA-KIPAN(N=868)
r=0.43
p=8.2e-40
TCGA-COADREAD(N=365)
r=0.57
p=1.3e-32
TCGA-BRCA(N=1061)
r=0.51
p=7.6e-71
ImmuneScore
TCGA-KIPAN(N=868)
r=0.45
p=1.9e-44
TCGA-COADREAD(N=365)
r=0.64
p=2.5e-43

APOBEC3B

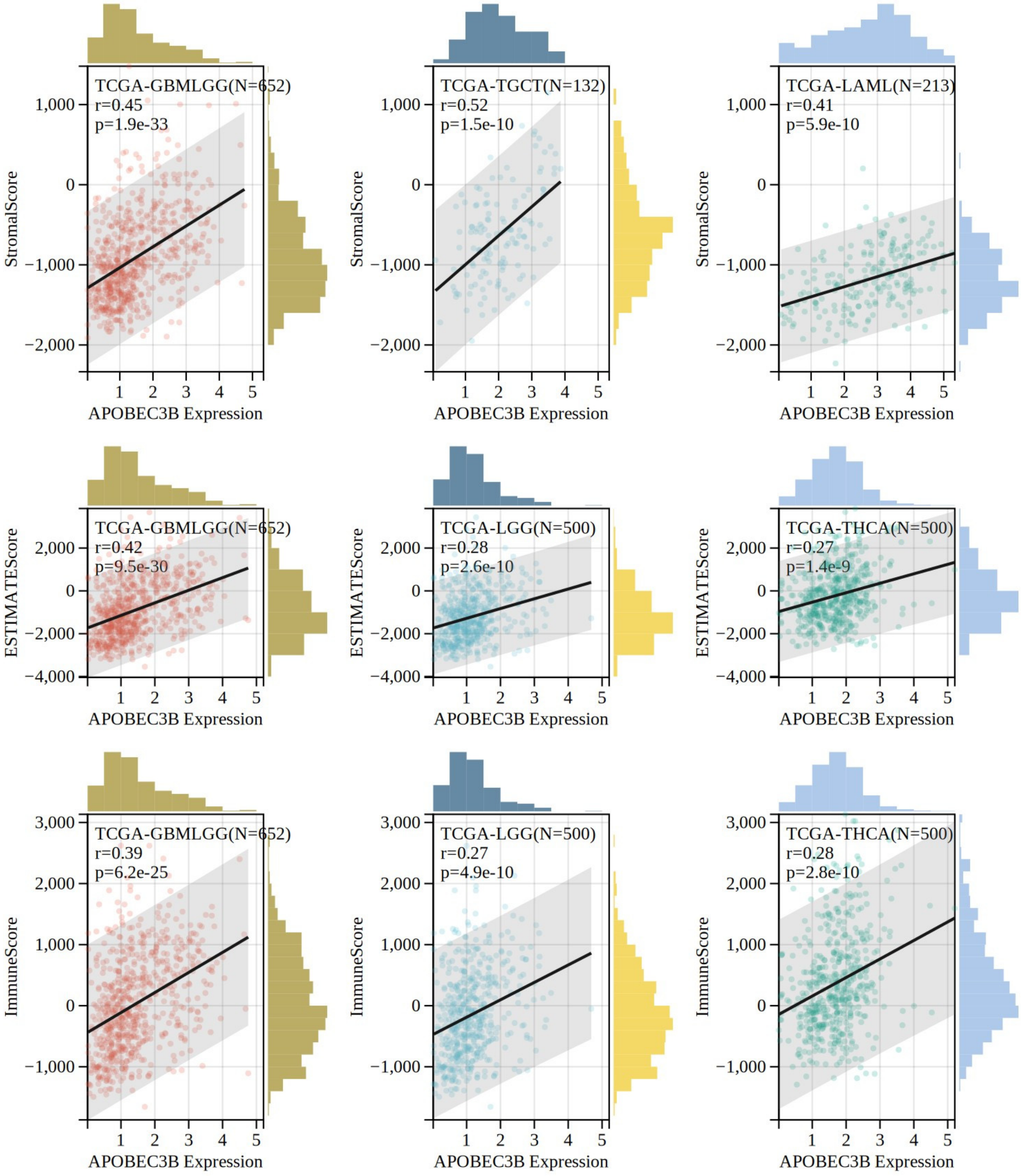

TCGA-GBMLGG(N=652)
r=0.45
p=1.9e-33
StromalScore
APOBEC3B Expression
TCGA-TGCT(N=132)
r=0.52
p=1.5e-10
StromalScore
APOBEC3B Expression
TCGA-LAML(N=213)
r=0.41
p=5.9e-10
StromalScore
APOBEC3B Expression
TCGA-GBMLGG(N=652)
r=0.42
p=9.5e-30
ESTIMATEScore
APOBEC3B Expression
TCGA-LGG(N=500)
r=0.28
p=2.6e-10
ESTIMATEScore
APOBEC3B Expression
TCGA-THCA(N=500)
r=0.27
p=1.4e-9
ESTIMATEScore
APOBEC3B Expression
TCGA-GBMLGG(N=652)
r=0.39
p=6.2e-25
ImmuneScore
APOBEC3B Expression
TCGA-LGG(N=500)
r=0.27
p=4.9e-10
ImmuneScore
APOBEC3B Expression
TCGA-THCA(N=500)
r=0.28
p=2.8e-10
ImmuneScore
APOBEC3B Expression

APOBEC3C

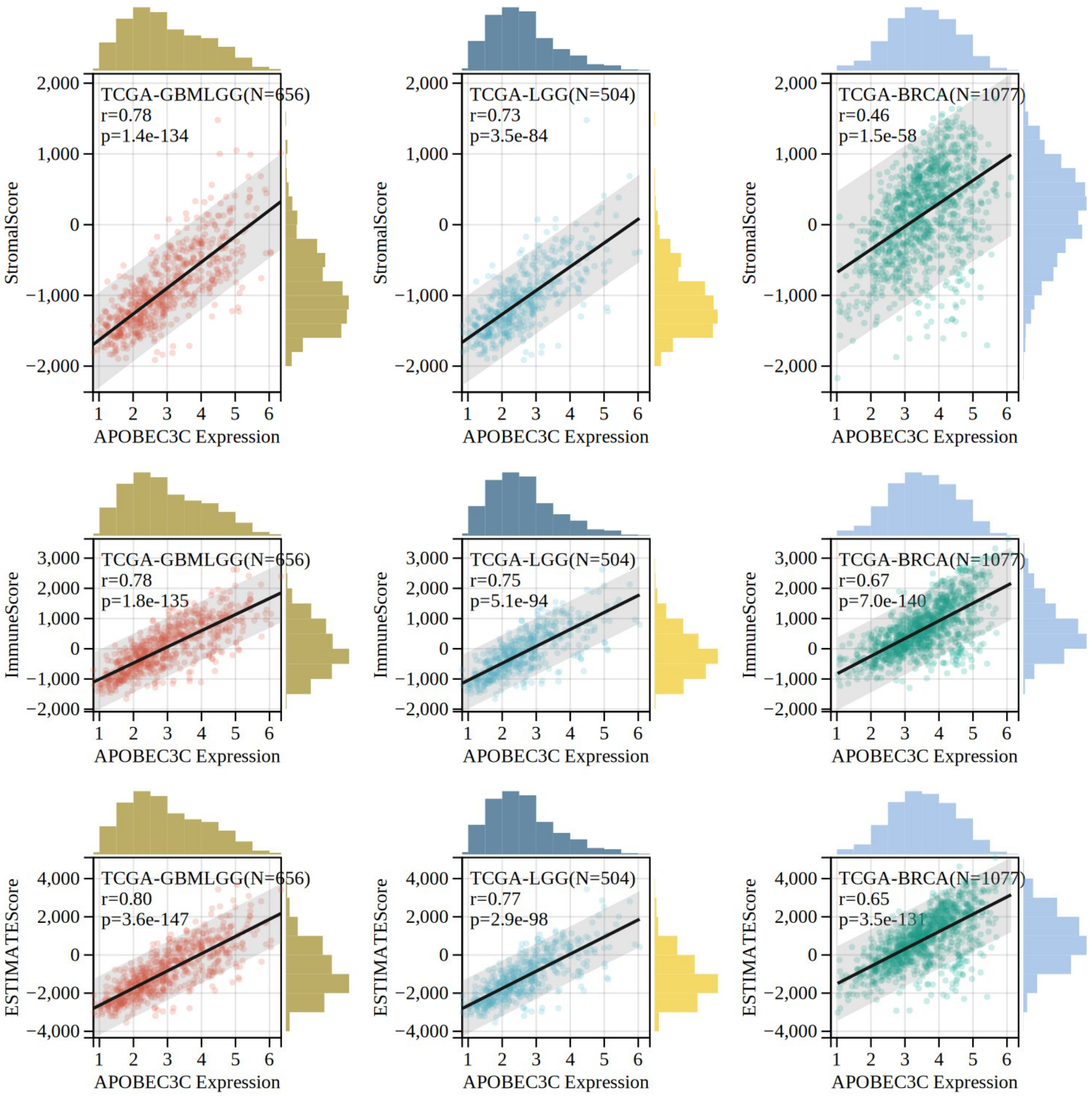

TCGA-GBMLGG(N=656)
r=0.78
p=1.4e-134
StromalScore
APOBEC3C Expression
TCGA-LGG(N=504)
r=0.73
p=3.5e-84
StromalScore
APOBEC3C Expression
TCGA-BRCA(N=1077)
r=0.46
p=1.5e-58
StromalScore
APOBEC3C Expression
TCGA-GBMLGG(N=656)
r=0.78
p=1.8e-135
ImmuneScore
APOBEC3C Expression
TCGA-LGG(N=504)
r=0.75
p=5.1e-94
ImmuneScore
APOBEC3C Expression
TCGA-BRCA(N=1077)
r=0.67
p=7.0e-140
ImmuneScore
APOBEC3C Expression
TCGA-GBMLGG(N=656)
r=0.80
p=3.6e-147
ESTIMATEScore
APOBEC3C Expression
TCGA-LGG(N=504)
r=0.77
p=2.9e-98
ESTIMATEScore
APOBEC3C Expression
TCGA-BRCA(N=1077)
r=0.65
p=3.5e-131
ESTIMATEScore
APOBEC3C Expression

APOBEC3D

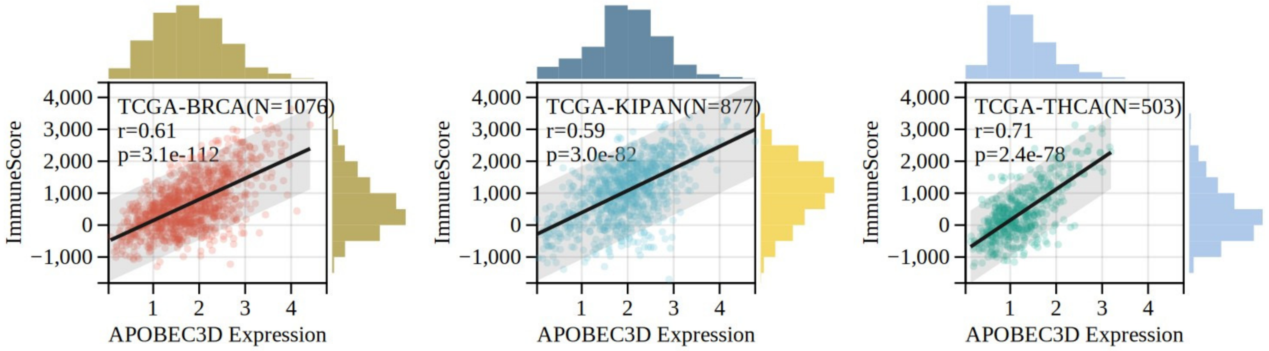

TCGA-BRCA(N=1076)
r=0.61
p=3.1e-112
ImmuneScore
APOBEC3D Expression
TCGA-KIPAN(N=877)
r=0.59
p=3.0e-82
ImmuneScore
APOBEC3D Expression
TCGA-THCA(N=503)
r=0.71
p=2.4e-78
ImmuneScore
APOBEC3D Expression

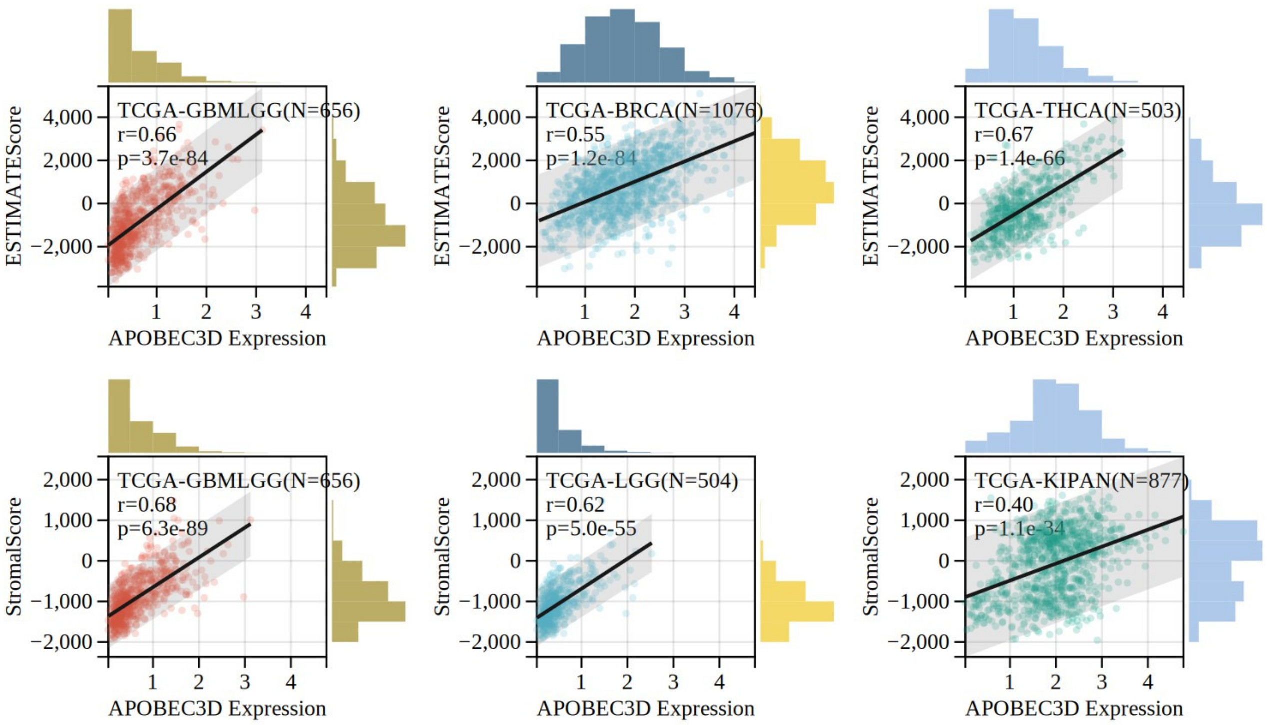
TCGA-GBMLGG(N=656)
r=0.66
p=3.7e-84
ESTIMATEScore
APOBEC3D Expression
TCGA-BRCA(N=1076)
r=0.55
p=1.2e-84
ESTIMATEScore
APOBEC3D Expression
TCGA-THCA(N=503)
r=0.67
p=1.4e-66
ESTIMATEScore
APOBEC3D Expression
TCGA-GBMLGG(N=656)
r=0.68
p=6.3e-89
StromalScore
APOBEC3D Expression
TCGA-LGG(N=504)
r=0.62
p=5.0e-55
StromalScore
APOBEC3D Expression
TCGA-KIPAN(N=877)
r=0.40
p=1.1e-34
StromalScore
APOBEC3D Expression

APOBEC3F

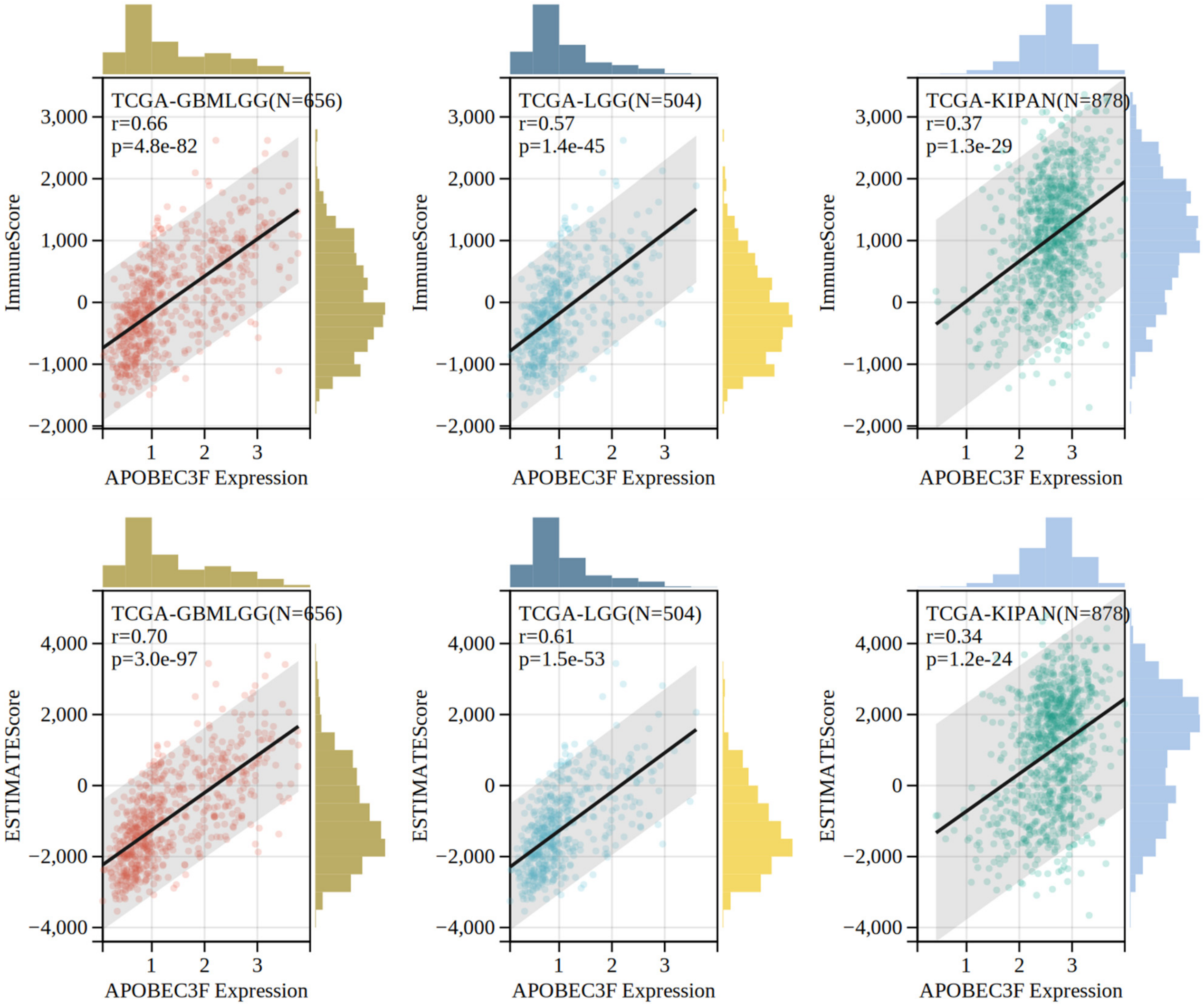
TCGA-GBMLGG(N=656)
r=0.66
p=4.8e-82
ImmuneScore
APOBEC3F Expression
TCGA-LGG(N=504)
r=0.57
p=1.4e-45
ImmuneScore
APOBEC3F Expression
TCGA-KIPAN(N=878)
r=0.37
p=1.3e-29
ImmuneScore
APOBEC3F Expression
TCGA-GBMLGG(N=656)
r=0.70
p=3.0e-97
ESTIMATEScore
APOBEC3F Expression
TCGA-LGG(N=504)
r=0.61
p=1.5e-53
ESTIMATEScore
APOBEC3F Expression
TCGA-KIPAN(N=878)
r=0.34
p=1.2e-24
ESTIMATEScore
APOBEC3F Expression

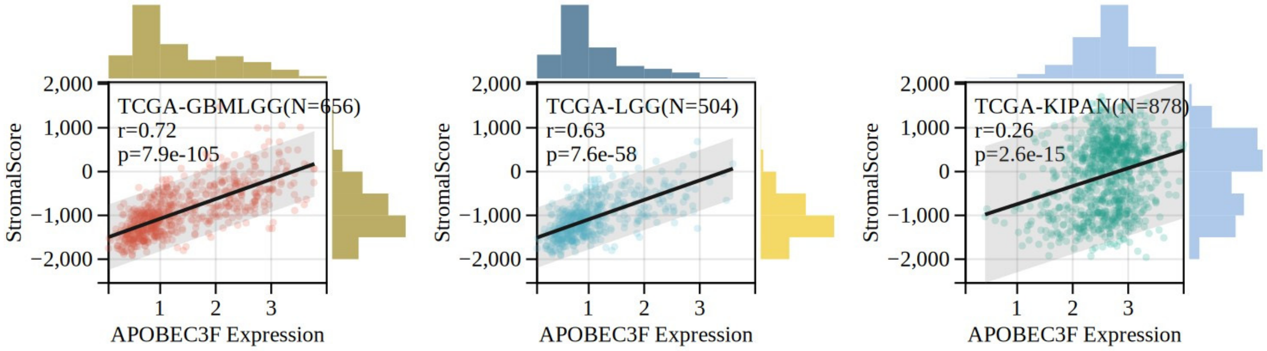
TCGA-GBMLGG(N=656)
r=0.72
p=7.9e-105
TCGA-LGG(N=504)
r=0.63
p=7.6e-58
TCGA-KIPAN(N=878)
r=0.26
p=2.6e-15
StromalScore
APOBEC3F Expression
2,000
1,000
0
−1,000
−2,000
1
2
3

APOBEC3G

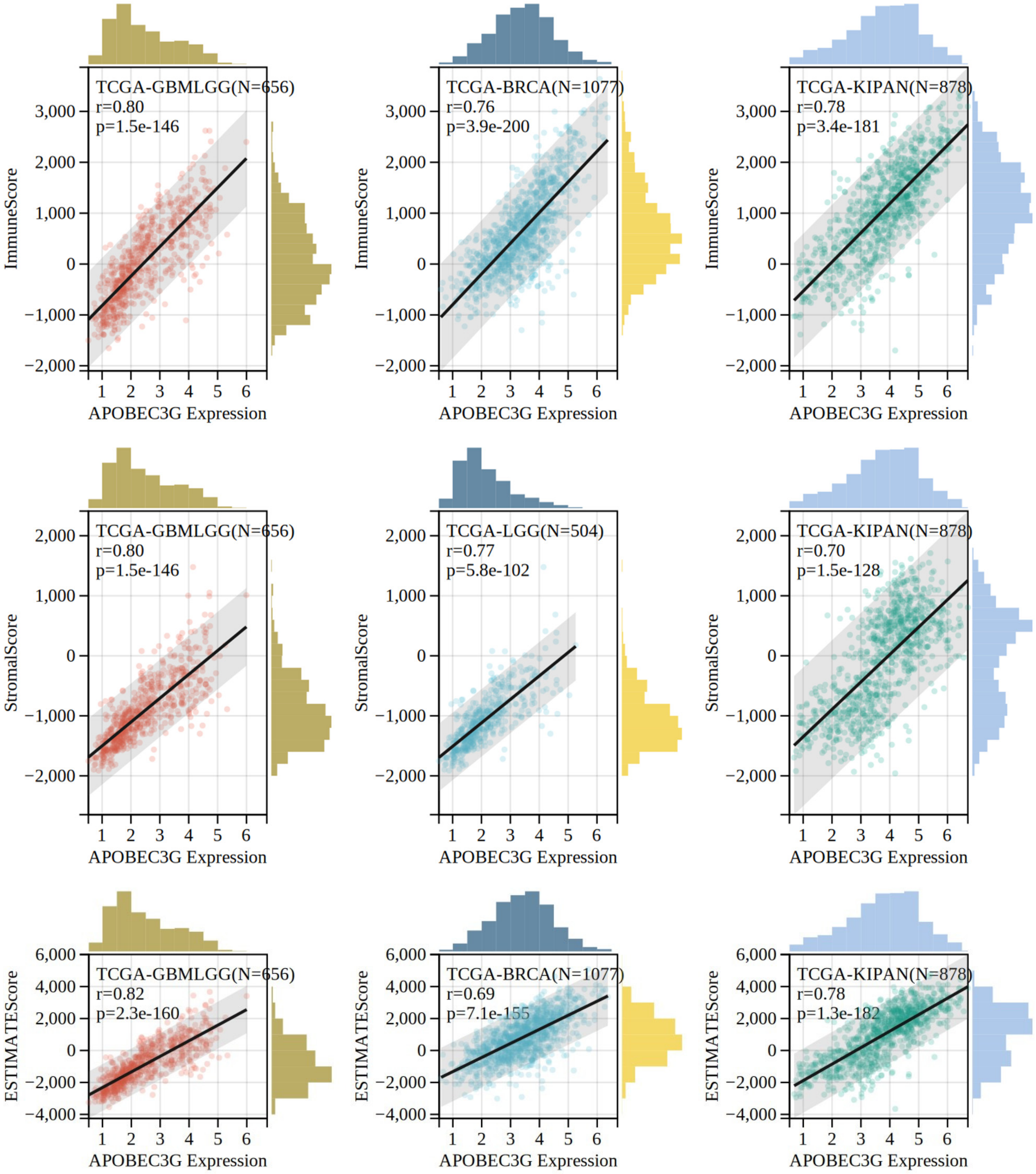
TCGA-GBMLGG(N=656)
r=0.80
p=1.5e-146
TCGA-BRCA(N=1077)
r=0.76
p=3.9e-200
TCGA-KIPAN(N=878)
r=0.78
p=3.4e-181
ImmuneScore
TCGA-GBMLGG(N=656)
r=0.80
p=1.5e-146
TCGA-LGG(N=504)
r=0.77
p=5.8e-102
TCGA-KIPAN(N=878)
r=0.70
p=1.5e-128
StromalScore
TCGA-GBMLGG(N=656)
r=0.82
p=2.3e-160
TCGA-BRCA(N=1077)
r=0.69
p=7.1e-155
TCGA-KIPAN(N=878)
r=0.78
p=1.3e-182
ESTIMATEScore
APOBEC3G Expression
3,000
2,000
1,000
0
−1,000
−2,000
6,000
4,000
−4,000
1
2
3
4
5
6

APOBEC3H

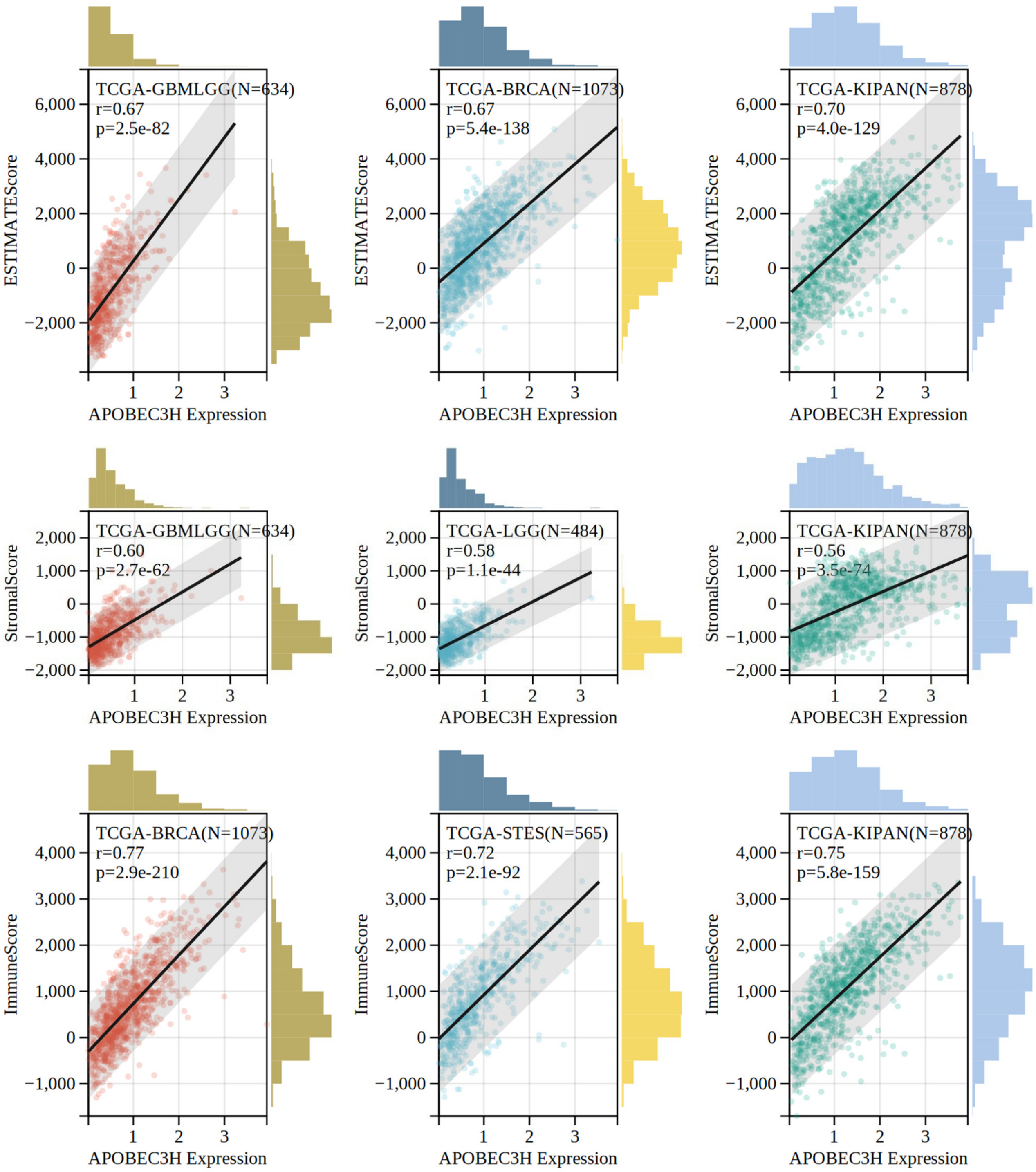

APOBEC4

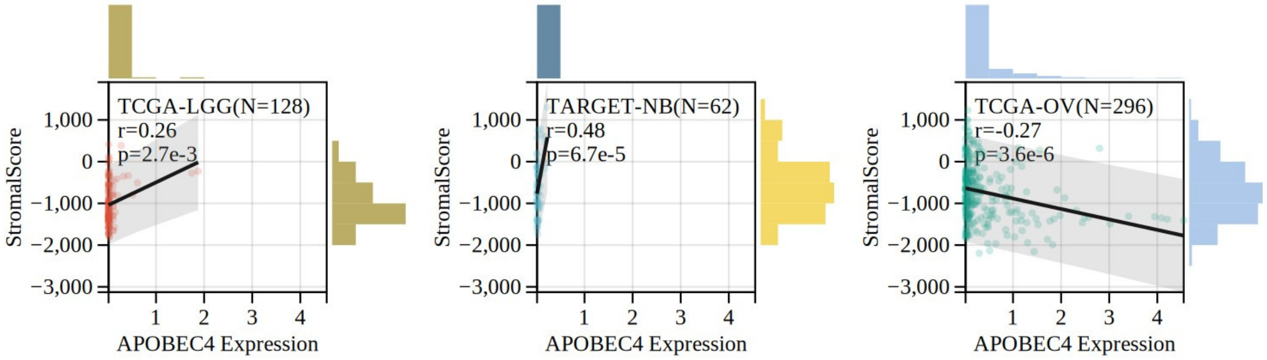
TCGA-LGG(N=128)
r=0.26
p=2.7e-3
StromalScore
APOBEC4 Expression
TARGET-NB(N=62)
r=0.48
p=6.7e-5
TCGA-OV(N=296)
r=-0.27
p=3.6e-6

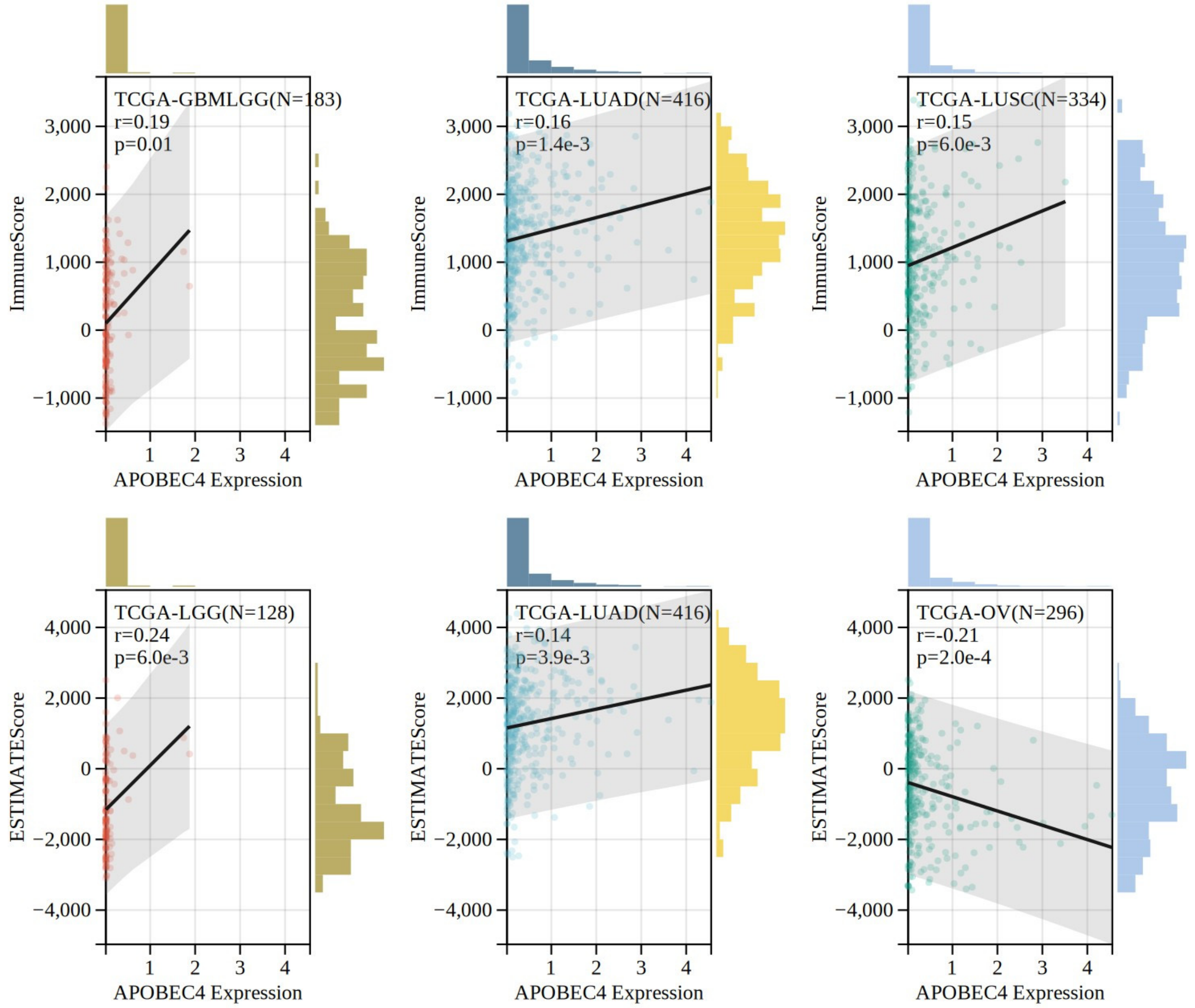

TCGA-GBMLGG(N=183)
r=0.19
p=0.01
TCGA-LUAD(N=416)
r=0.16
p=1.4e-3
TCGA-LUSC(N=334)
r=0.15
p=6.0e-3
ImmuneScore
TCGA-LGG(N=128)
r=0.24
p=6.0e-3
TCGA-LUAD(N=416)
r=0.14
p=3.9e-3
TCGA-OV(N=296)
r=-0.21
p=2.0e-4
ESTIMATEScore
APOBEC4 Expression

Table S6. APOBEC–ESTIMATE correlations per gene (Immune, Stromal, overall); Immune mean r as in Table 4.

Gene	Immune nSig	Immune +/−	Stromal nSig	Stromal +/−	ESTIMATE nSig	ESTIMATE +/−	Immune mean r	P–S agree %
**APOBEC1**	5	0/5	11	2/9	7	0/7	−0.05	100
**APOBEC2**	11	6/5	15	10/5	13	7/6	−0.03	100
**APOBEC3A**	40	40/0	33	33/0	39	39/0	0.38	100
**APOBEC3B**	18	13/5	16	9/7	17	11/6	0.06	100
**APOBEC3C**	38	38/0	34	34/0	39	39/0	0.39	100
**APOBEC3D**	40	39/1	34	34/0	40	40/0	0.46	100
**APOBEC3F**	32	31/1	27	26/1	28	27/1	0.25	100
**APOBEC3G**	40	40/0	39	38/1	40	40/0	0.56	100
**APOBEC3H**	40	40/0	38	38/0	41	41/0	0.57	100
**APOBEC4**	8	4/4	8	6/2	8	5/3	0.03	100

Figure S6. TIMER immune-infiltration correlations (six lineages) for the ten APOBEC genes. Significance, nominal $p < 0.05$ (see Table S14).

APOBEC1

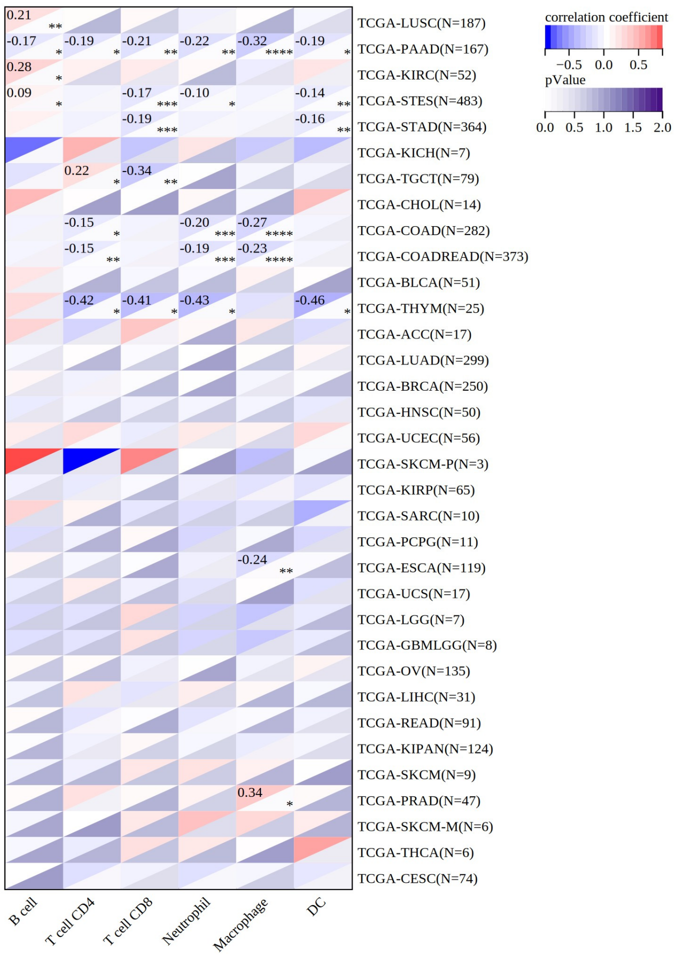

APOBEC2

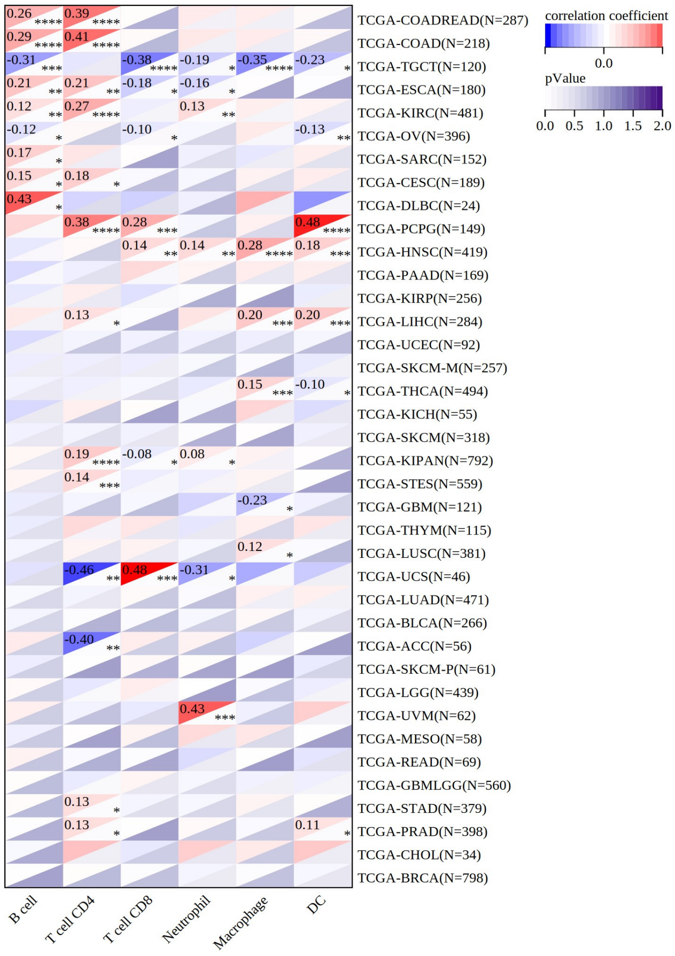
correlation coefficient
0.0
pValue
0.0 0.5 1.0 1.5 2.0
TCGA-COADREAD(N=287)
TCGA-COAD(N=218)
TCGA-TGCT(N=120)
TCGA-ESCA(N=180)
TCGA-KIRC(N=481)
TCGA-OV(N=396)
TCGA-SARC(N=152)
TCGA-CESC(N=189)
TCGA-DLBC(N=24)
TCGA-PCPG(N=149)
TCGA-HNSC(N=419)
TCGA-PAAD(N=169)
TCGA-KIRP(N=256)
TCGA-LIHC(N=284)
TCGA-UCEC(N=92)
TCGA-SKCM-M(N=257)
TCGA-THCA(N=494)
TCGA-KICH(N=55)
TCGA-SKCM(N=318)
TCGA-KIPAN(N=792)
TCGA-STES(N=559)
TCGA-GBM(N=121)
TCGA-THYM(N=115)
TCGA-LUSC(N=381)
TCGA-UCS(N=46)
TCGA-LUAD(N=471)
TCGA-BLCA(N=266)
TCGA-ACC(N=56)
TCGA-SKCM-P(N=61)
TCGA-LGG(N=439)
TCGA-UVM(N=62)
TCGA-MESO(N=58)
TCGA-READ(N=69)
TCGA-GBMLGG(N=560)
TCGA-STAD(N=379)
TCGA-PRAD(N=398)
TCGA-CHOL(N=34)
TCGA-BRCA(N=798)
B cell
T cell CD4
T cell CD8
Neutrophil
Macrophage
DC

APOBEC3A

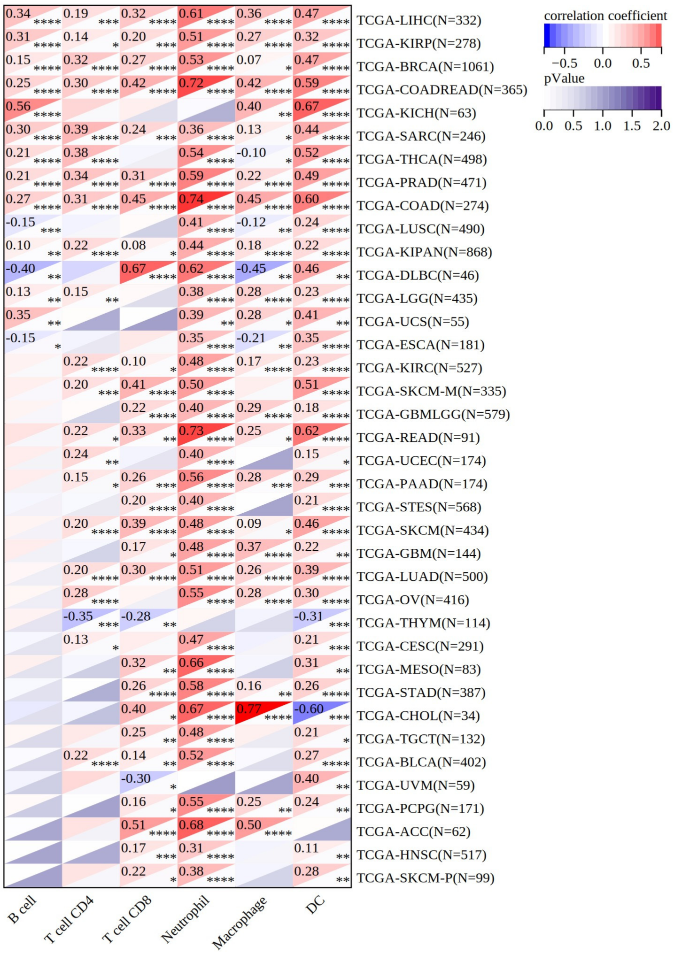
correlation coefficient
−0.5 0.0 0.5
pValue
0.0 0.5 1.0 1.5 2.0
TCGA-LIHC(N=332)
TCGA-KIRP(N=278)
TCGA-BRCA(N=1061)
TCGA-COADREAD(N=365)
TCGA-KICH(N=63)
TCGA-SARC(N=246)
TCGA-THCA(N=498)
TCGA-PRAD(N=471)
TCGA-COAD(N=274)
TCGA-LUSC(N=490)
TCGA-KIPAN(N=868)
TCGA-DLBC(N=46)
TCGA-LGG(N=435)
TCGA-UCS(N=55)
TCGA-ESCA(N=181)
TCGA-KIRC(N=527)
TCGA-SKCM-M(N=335)
TCGA-GBMLGG(N=579)
TCGA-READ(N=91)
TCGA-UCEC(N=174)
TCGA-PAAD(N=174)
TCGA-STES(N=568)
TCGA-SKCM(N=434)
TCGA-GBM(N=144)
TCGA-LUAD(N=500)
TCGA-OV(N=416)
TCGA-THYM(N=114)
TCGA-CESC(N=291)
TCGA-MESO(N=83)
TCGA-STAD(N=387)
TCGA-CHOL(N=34)
TCGA-TGCT(N=132)
TCGA-BLCA(N=402)
TCGA-UVM(N=59)
TCGA-PCPG(N=171)
TCGA-ACC(N=62)
TCGA-HNSC(N=517)
TCGA-SKCM-P(N=99)
B cell
T cell CD4
T cell CD8
Neutrophil
Macrophage
DC

APOBEC3B

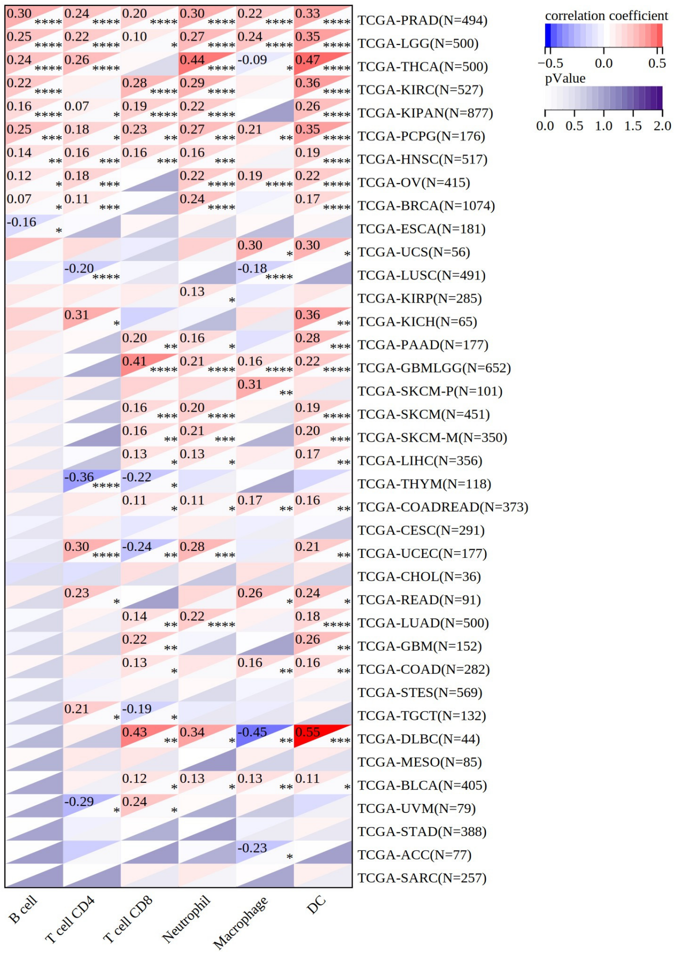
correlation coefficient
−0.5
0.0
0.5
pValue
0.0
0.5
1.0
1.5
2.0
TCGA-PRAD(N=494)
TCGA-LGG(N=500)
TCGA-THCA(N=500)
TCGA-KIRC(N=527)
TCGA-KIPAN(N=877)
TCGA-PCPG(N=176)
TCGA-HNSC(N=517)
TCGA-OV(N=415)
TCGA-BRCA(N=1074)
TCGA-ESCA(N=181)
TCGA-UCS(N=56)
TCGA-LUSC(N=491)
TCGA-KIRP(N=285)
TCGA-KICH(N=65)
TCGA-PAAD(N=177)
TCGA-GBMLGG(N=652)
TCGA-SKCM-P(N=101)
TCGA-SKCM(N=451)
TCGA-SKCM-M(N=350)
TCGA-LIHC(N=356)
TCGA-THYM(N=118)
TCGA-COADREAD(N=373)
TCGA-CESC(N=291)
TCGA-UCEC(N=177)
TCGA-CHOL(N=36)
TCGA-READ(N=91)
TCGA-LUAD(N=500)
TCGA-GBM(N=152)
TCGA-COAD(N=282)
TCGA-STES(N=569)
TCGA-TGCT(N=132)
TCGA-DLBC(N=44)
TCGA-MESO(N=85)
TCGA-BLCA(N=405)
TCGA-UVM(N=79)
TCGA-STAD(N=388)
TCGA-ACC(N=77)
TCGA-SARC(N=257)
B cell
T cell CD4
T cell CD8
Neutrophil
Macrophage
DC

APOBEC3C

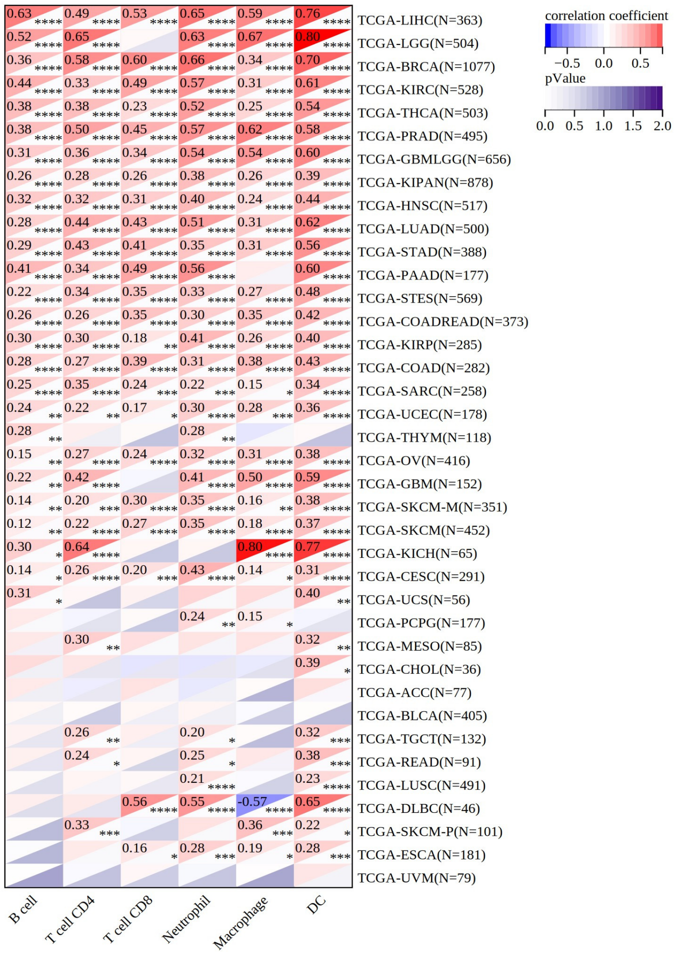
correlation coefficient
−0.5 0.0 0.5
pValue
0.0 0.5 1.0 1.5 2.0
TCGA-LIHC(N=363)
TCGA-LGG(N=504)
TCGA-BRCA(N=1077)
TCGA-KIRC(N=528)
TCGA-THCA(N=503)
TCGA-PRAD(N=495)
TCGA-GBMLGG(N=656)
TCGA-KIPAN(N=878)
TCGA-HNSC(N=517)
TCGA-LUAD(N=500)
TCGA-STAD(N=388)
TCGA-PAAD(N=177)
TCGA-STES(N=569)
TCGA-COADREAD(N=373)
TCGA-KIRP(N=285)
TCGA-COAD(N=282)
TCGA-SARC(N=258)
TCGA-UCEC(N=178)
TCGA-THYM(N=118)
TCGA-OV(N=416)
TCGA-GBM(N=152)
TCGA-SKCM-M(N=351)
TCGA-SKCM(N=452)
TCGA-KICH(N=65)
TCGA-CESC(N=291)
TCGA-UCS(N=56)
TCGA-PCPG(N=177)
TCGA-MESO(N=85)
TCGA-CHOL(N=36)
TCGA-ACC(N=77)
TCGA-BLCA(N=405)
TCGA-TGCT(N=132)
TCGA-READ(N=91)
TCGA-LUSC(N=491)
TCGA-DLBC(N=46)
TCGA-SKCM-P(N=101)
TCGA-ESCA(N=181)
TCGA-UVM(N=79)
B cell
T cell CD4
T cell CD8
Neutrophil
Macrophage
DC

APOBEC3D

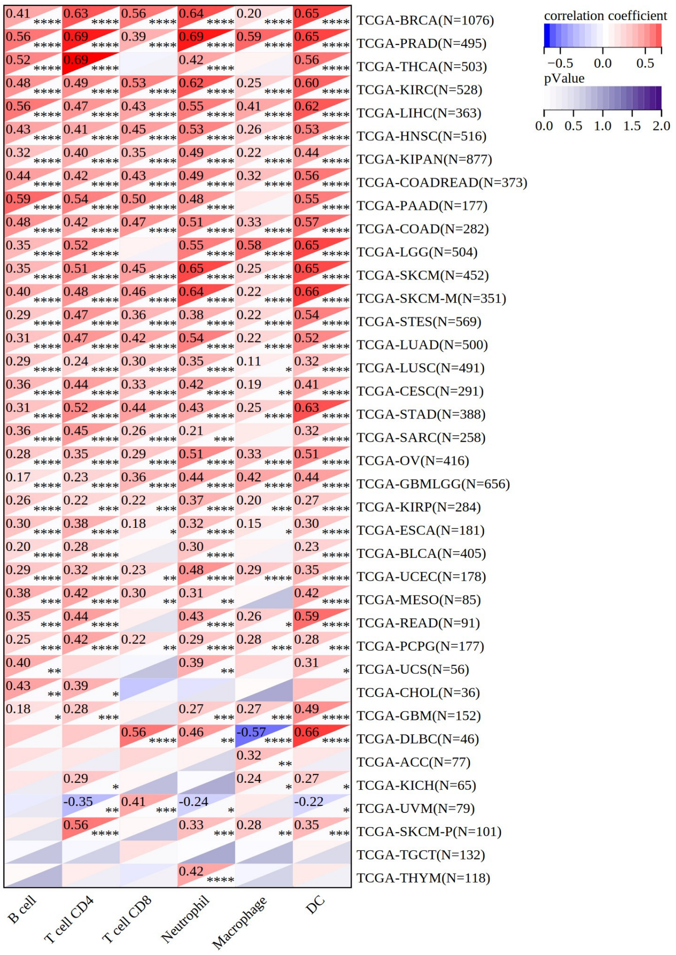
correlation coefficient
−0.5 0.0 0.5
pValue
0.0 0.5 1.0 1.5 2.0
TCGA-BRCA(N=1076)
TCGA-PRAD(N=495)
TCGA-THCA(N=503)
TCGA-KIRC(N=528)
TCGA-LIHC(N=363)
TCGA-HNSC(N=516)
TCGA-KIPAN(N=877)
TCGA-COADREAD(N=373)
TCGA-PAAD(N=177)
TCGA-COAD(N=282)
TCGA-LGG(N=504)
TCGA-SKCM(N=452)
TCGA-SKCM-M(N=351)
TCGA-STES(N=569)
TCGA-LUAD(N=500)
TCGA-LUSC(N=491)
TCGA-CESC(N=291)
TCGA-STAD(N=388)
TCGA-SARC(N=258)
TCGA-OV(N=416)
TCGA-GBMLGG(N=656)
TCGA-KIRP(N=284)
TCGA-ESCA(N=181)
TCGA-BLCA(N=405)
TCGA-UCEC(N=178)
TCGA-MESO(N=85)
TCGA-READ(N=91)
TCGA-PCPG(N=177)
TCGA-UCS(N=56)
TCGA-CHOL(N=36)
TCGA-GBM(N=152)
TCGA-DLBC(N=46)
TCGA-ACC(N=77)
TCGA-KICH(N=65)
TCGA-UVM(N=79)
TCGA-SKCM-P(N=101)
TCGA-TGCT(N=132)
TCGA-THYM(N=118)
B cell
T cell CD4
T cell CD8
Neutrophil
Macrophage
DC

APOBEC3F

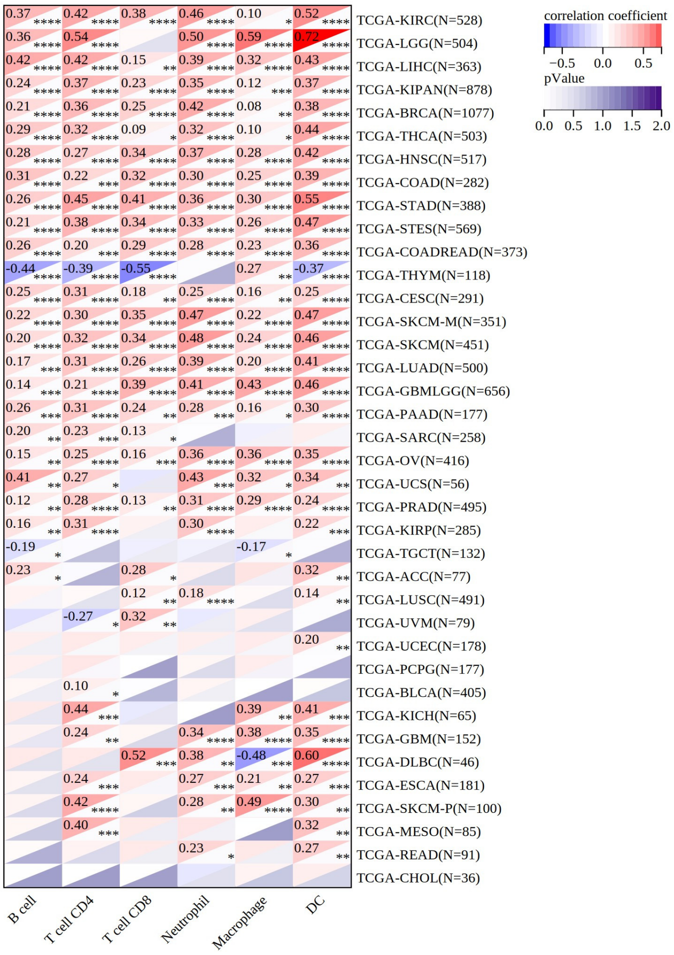
correlation coefficient
−0.5 0.0 0.5
pValue
0.0 0.5 1.0 1.5 2.0
TCGA-KIRC(N=528)
TCGA-LGG(N=504)
TCGA-LIHC(N=363)
TCGA-KIPAN(N=878)
TCGA-BRCA(N=1077)
TCGA-THCA(N=503)
TCGA-HNSC(N=517)
TCGA-COAD(N=282)
TCGA-STAD(N=388)
TCGA-STES(N=569)
TCGA-COADREAD(N=373)
TCGA-THYM(N=118)
TCGA-CESC(N=291)
TCGA-SKCM-M(N=351)
TCGA-SKCM(N=451)
TCGA-LUAD(N=500)
TCGA-GBMLGG(N=656)
TCGA-PAAD(N=177)
TCGA-SARC(N=258)
TCGA-OV(N=416)
TCGA-UCS(N=56)
TCGA-PRAD(N=495)
TCGA-KIRP(N=285)
TCGA-TGCT(N=132)
TCGA-ACC(N=77)
TCGA-LUSC(N=491)
TCGA-UVM(N=79)
TCGA-UCEC(N=178)
TCGA-PCPG(N=177)
TCGA-BLCA(N=405)
TCGA-KICH(N=65)
TCGA-GBM(N=152)
TCGA-DLBC(N=46)
TCGA-ESCA(N=181)
TCGA-SKCM-P(N=100)
TCGA-MESO(N=85)
TCGA-READ(N=91)
TCGA-CHOL(N=36)
B cell
T cell CD4
T cell CD8
Neutrophil
Macrophage
DC

APOBEC3G

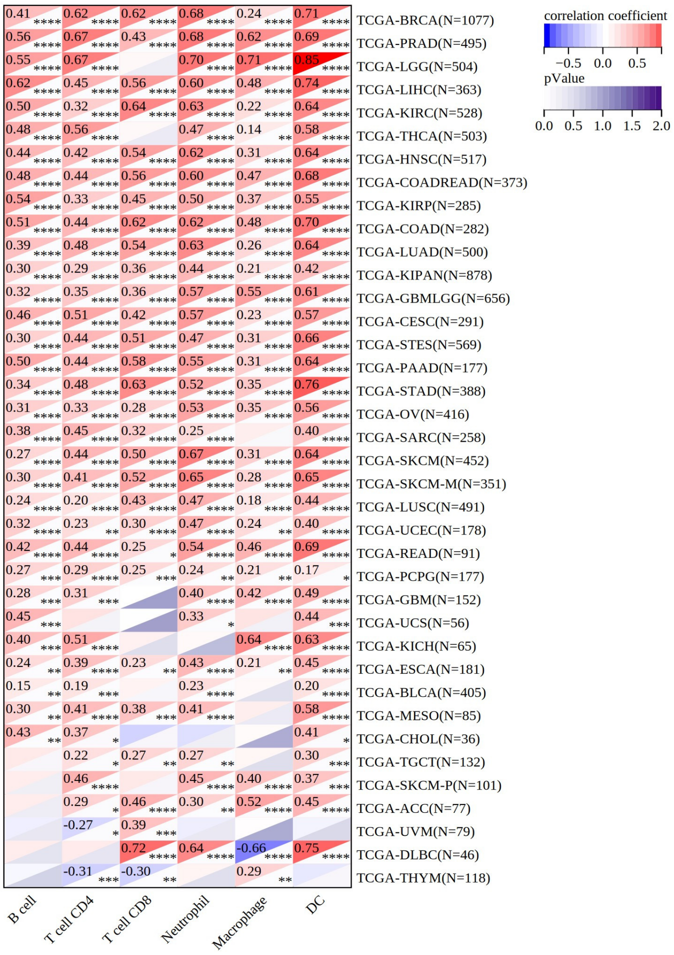
correlation coefficient
pValue
B cell
T cell CD4
T cell CD8
Neutrophil
Macrophage
DC
TCGA-BRCA(N=1077)
TCGA-PRAD(N=495)
TCGA-LGG(N=504)
TCGA-LIHC(N=363)
TCGA-KIRC(N=528)
TCGA-THCA(N=503)
TCGA-HNSC(N=517)
TCGA-COADREAD(N=373)
TCGA-KIRP(N=285)
TCGA-COAD(N=282)
TCGA-LUAD(N=500)
TCGA-KIPAN(N=878)
TCGA-GBMLGG(N=656)
TCGA-CESC(N=291)
TCGA-STES(N=569)
TCGA-PAAD(N=177)
TCGA-STAD(N=388)
TCGA-OV(N=416)
TCGA-SARC(N=258)
TCGA-SKCM(N=452)
TCGA-SKCM-M(N=351)
TCGA-LUSC(N=491)
TCGA-UCEC(N=178)
TCGA-READ(N=91)
TCGA-PCPG(N=177)
TCGA-GBM(N=152)
TCGA-UCS(N=56)
TCGA-KICH(N=65)
TCGA-ESCA(N=181)
TCGA-BLCA(N=405)
TCGA-MESO(N=85)
TCGA-CHOL(N=36)
TCGA-TGCT(N=132)
TCGA-SKCM-P(N=101)
TCGA-ACC(N=77)
TCGA-UVM(N=79)
TCGA-DLBC(N=46)
TCGA-THYM(N=118)

APOBEC3H

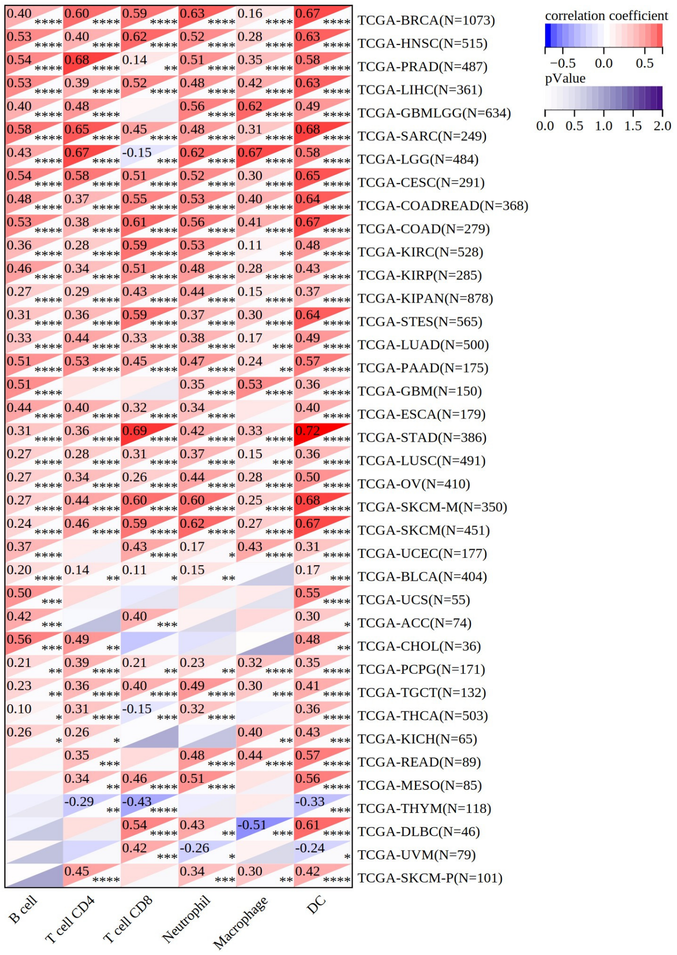

APOBEC4

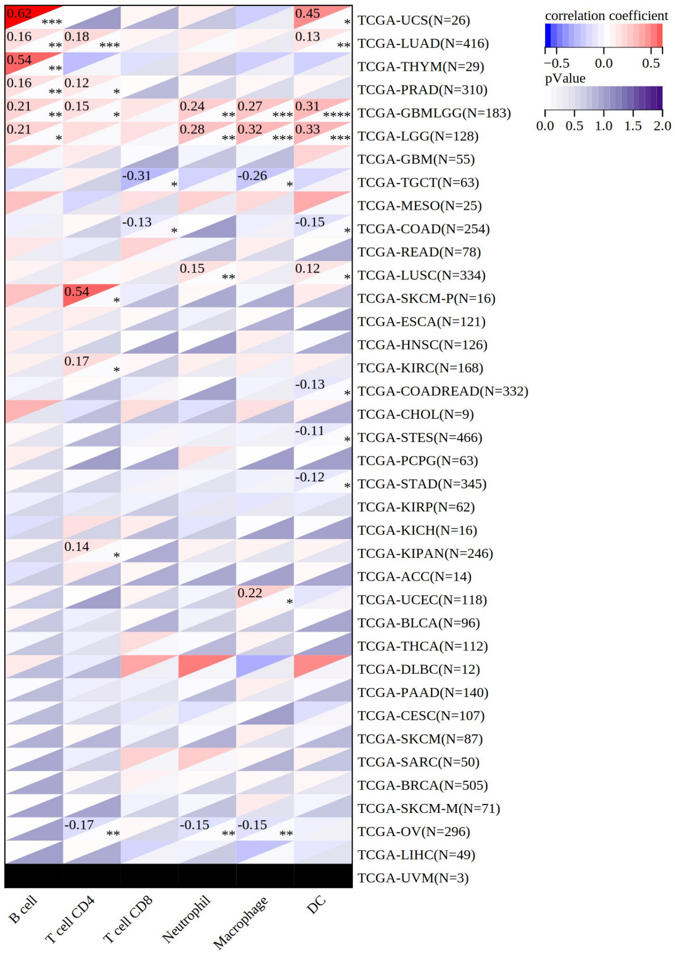
correlation coefficient
−0.5
0.0
0.5
pValue
0.0
0.5
1.0
1.5
2.0
TCGA-UCS(N=26)
TCGA-LUAD(N=416)
TCGA-THYM(N=29)
TCGA-PRAD(N=310)
TCGA-GBMLGG(N=183)
TCGA-LGG(N=128)
TCGA-GBM(N=55)
TCGA-TGCT(N=63)
TCGA-MESO(N=25)
TCGA-COAD(N=254)
TCGA-READ(N=78)
TCGA-LUSC(N=334)
TCGA-SKCM-P(N=16)
TCGA-ESCA(N=121)
TCGA-HNSC(N=126)
TCGA-KIRC(N=168)
TCGA-COADREAD(N=332)
TCGA-CHOL(N=9)
TCGA-STES(N=466)
TCGA-PCPG(N=63)
TCGA-STAD(N=345)
TCGA-KIRP(N=62)
TCGA-KICH(N=16)
TCGA-KIPAN(N=246)
TCGA-ACC(N=14)
TCGA-UCEC(N=118)
TCGA-BLCA(N=96)
TCGA-THCA(N=112)
TCGA-DLBC(N=12)
TCGA-PAAD(N=140)
TCGA-CESC(N=107)
TCGA-SKCM(N=87)
TCGA-SARC(N=50)
TCGA-BRCA(N=505)
TCGA-SKCM-M(N=71)
TCGA-OV(N=296)
TCGA-LIHC(N=49)
TCGA-UVM(N=3)
B cell
T cell CD4
T cell CD8
Neutrophil
Macrophage
DC

Table S7. Pearson versus Spearman TIMER summary statistics, per gene.

Gene	Pearson n sig	Pearson mean r	Pearson % pos	Spearman n sig	Spearman mean ρ	Spearman % pos
**APOBEC1**	28	−0.16	17.9	36	−0.17	19.4
**APOBEC2**	49	+0.08	67.3	70	+0.09	77.1
**APOBEC3A**	164	+0.30	92.7	170	+0.32	94.7
**APOBEC3B**	105	+0.17	89.5	115	+0.18	91.3
**APOBEC3C**	167	+0.37	99.4	170	+0.38	97.6
**APOBEC3D**	186	+0.39	97.8	190	+0.38	97.4
**APOBEC3F**	165	+0.28	95.2	165	+0.28	94.5
**APOBEC3G**	199	+0.43	98.0	199	+0.43	97.5
**APOBEC3H**	195	+0.40	95.9	194	+0.38	95.4
**APOBEC4**	33	+0.13	69.7	39	+0.01	56.4

APOBEC4 was the main exception, with its mean Pearson correlation (+0.13) dropping to ρ = +0.01 with Spearman, whereas APOBEC1 remained net-negative under both methods.

Table S8. Per-gene summary of TIMER correlations across the six immune lineages.

Gene	Samples (N)	Types Tested	Sig. Types	Mean r	% Positive
APOBEC1	2,526	34	11	−0.16	18%
APOBEC2	7,689	38	22	0.08	67%
APOBEC3A	9,142	38	38	0.30	93%
APOBEC3B	9,379	38	32	0.17	90%
APOBEC3C	9,405	38	35	0.37	99%
APOBEC3D	9,402	38	37	0.39	98%
APOBEC3F	9,404	38	36	0.28	95%
APOBEC3G	9,405	38	38	0.43	98%
APOBEC3H	9,328	38	38	0.40	96%
APOBEC4	4,217	38	17	0.13	70%

Figure S7. CIBERSORT 22-subset correlations for the ten APOBEC genes. Significance, nominal $p < 0.05$ (see Table S14).

APOBEC1

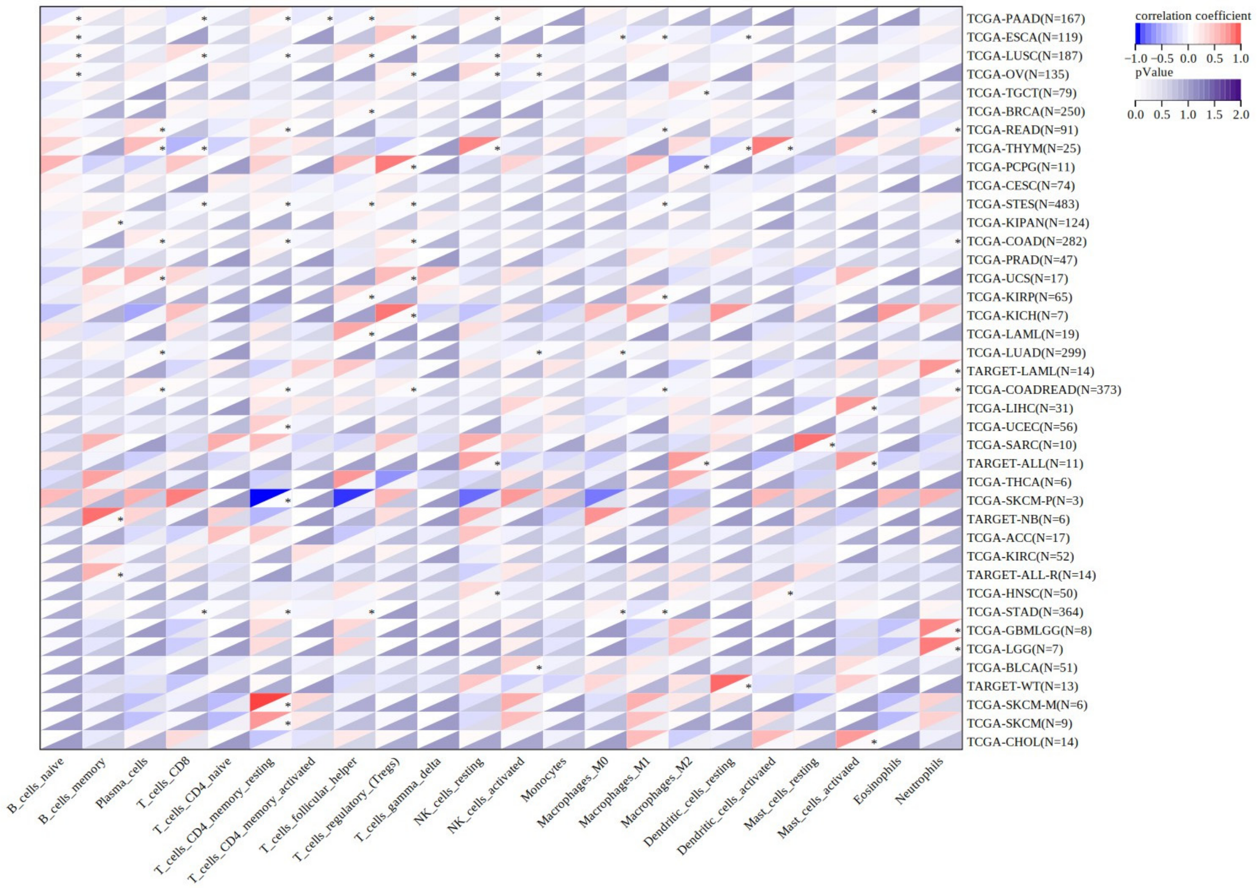
correlation coefficient
−1.0 −0.5 0.0 0.5 1.0
pValue
0.0 0.5 1.0 1.5 2.0
TCGA-PAAD(N=167)
TCGA-ESCA(N=119)
TCGA-LUSC(N=187)
TCGA-OV(N=135)
TCGA-TGCT(N=79)
TCGA-BRCA(N=250)
TCGA-READ(N=91)
TCGA-THYM(N=25)
TCGA-PCPG(N=11)
TCGA-CESC(N=74)
TCGA-STES(N=483)
TCGA-KIPAN(N=124)
TCGA-COAD(N=282)
TCGA-PRAD(N=47)
TCGA-UCS(N=17)
TCGA-KIRP(N=65)
TCGA-KICH(N=7)
TCGA-LAML(N=19)
TCGA-LUAD(N=299)
TARGET-LAML(N=14)
TCGA-COADREAD(N=373)
TCGA-LIHC(N=31)
TCGA-UCEC(N=56)
TCGA-SARC(N=10)
TARGET-ALL(N=11)
TCGA-THCA(N=6)
TCGA-SKCM-P(N=3)
TARGET-NB(N=6)
TCGA-ACC(N=17)
TCGA-KIRC(N=52)
TARGET-ALL-R(N=14)
TCGA-HNSC(N=50)
TCGA-STAD(N=364)
TCGA-GBMLGG(N=8)
TCGA-LGG(N=7)
TCGA-BLCA(N=51)
TARGET-WT(N=13)
TCGA-SKCM-M(N=6)
TCGA-SKCM(N=9)
TCGA-CHOL(N=14)
B_cells_naive
B_cells_memory
Plasma_cells
T_cells_CD8
T_cells_CD4_naive
T_cells_CD4_memory_resting
T_cells_CD4_memory_activated
T_cells_follicular_helper
T_cells_regulatory_(Tregs)
T_cells_gamma_delta
NK_cells_resting
NK_cells_activated
Monocytes
Macrophages_M0
Macrophages_M1
Macrophages_M2
Dendritic_cells_resting
Dendritic_cells_activated
Mast_cells_resting
Mast_cells_activated
Eosinophils
Neutrophils

APOBEC2

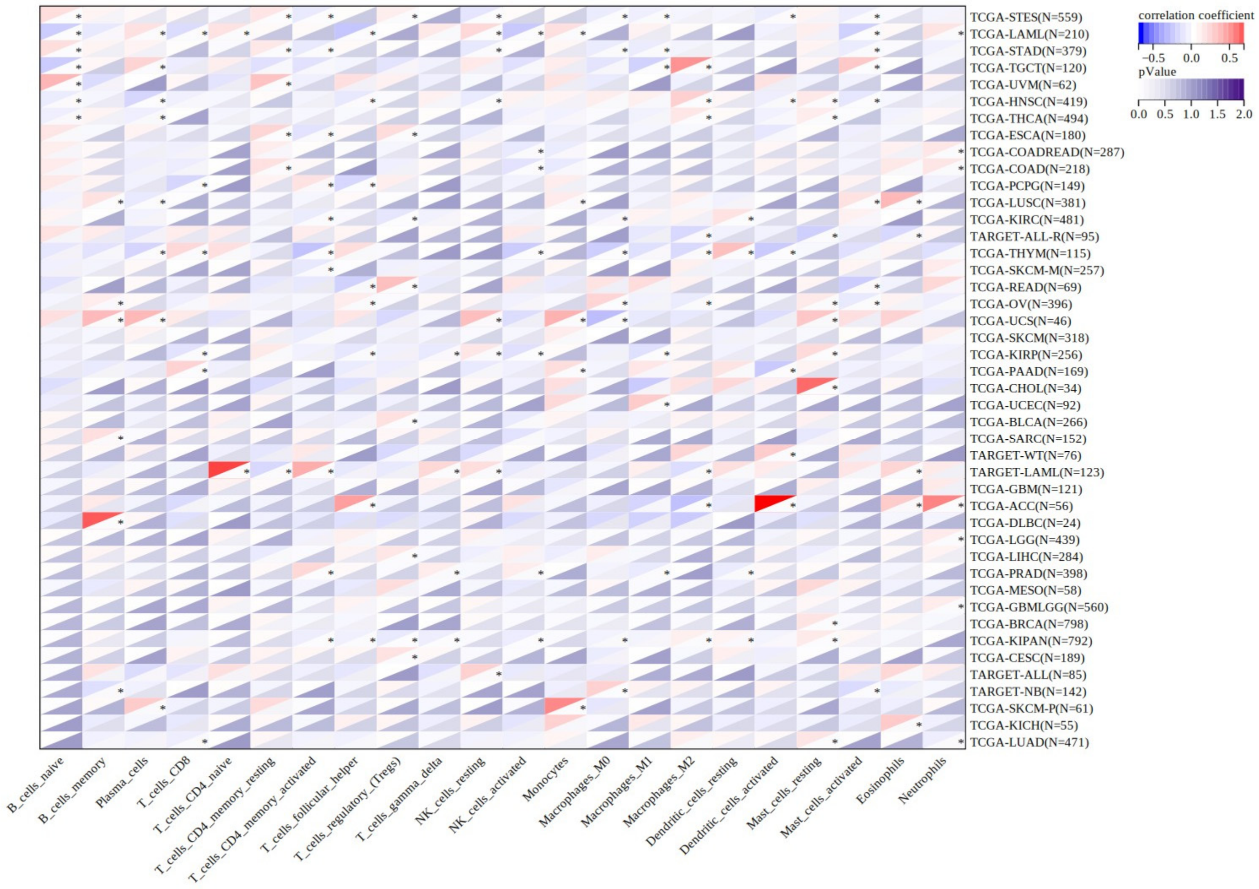
correlation coefficient
−0.5 0.0 0.5
pValue
0.0 0.5 1.0 1.5 2.0
TCGA-STES(N=559)
TCGA-LAML(N=210)
TCGA-STAD(N=379)
TCGA-TGCT(N=120)
TCGA-UVM(N=62)
TCGA-HNSC(N=419)
TCGA-THCA(N=494)
TCGA-ESCA(N=180)
TCGA-COADREAD(N=287)
TCGA-COAD(N=218)
TCGA-PCPG(N=149)
TCGA-LUSC(N=381)
TCGA-KIRC(N=481)
TARGET-ALL-R(N=95)
TCGA-THYM(N=115)
TCGA-SKCM-M(N=257)
TCGA-READ(N=69)
TCGA-OV(N=396)
TCGA-UCS(N=46)
TCGA-SKCM(N=318)
TCGA-KIRP(N=256)
TCGA-PAAD(N=169)
TCGA-CHOL(N=34)
TCGA-UCEC(N=92)
TCGA-BLCA(N=266)
TCGA-SARC(N=152)
TARGET-WT(N=76)
TARGET-LAML(N=123)
TCGA-GBM(N=121)
TCGA-ACC(N=56)
TCGA-DLBC(N=24)
TCGA-LGG(N=439)
TCGA-LIHC(N=284)
TCGA-PRAD(N=398)
TCGA-MESO(N=58)
TCGA-GBMLGG(N=560)
TCGA-BRCA(N=798)
TCGA-KIPAN(N=792)
TCGA-CESC(N=189)
TARGET-ALL(N=85)
TARGET-NB(N=142)
TCGA-SKCM-P(N=61)
TCGA-KICH(N=55)
TCGA-LUAD(N=471)
B_cells_naive
B_cells_memory
Plasma_cells
T_cells_CD8
T_cells_CD4_naive
T_cells_CD4_memory_resting
T_cells_CD4_memory_activated
T_cells_follicular_helper
T_cells_regulatory_(Tregs)
T_cells_gamma_delta
NK_cells_resting
NK_cells_activated
Monocytes
Macrophages_M0
Macrophages_M1
Macrophages_M2
Dendritic_cells_resting
Dendritic_cells_activated
Mast_cells_resting
Mast_cells_activated
Eosinophils
Neutrophils

APOBEC3A

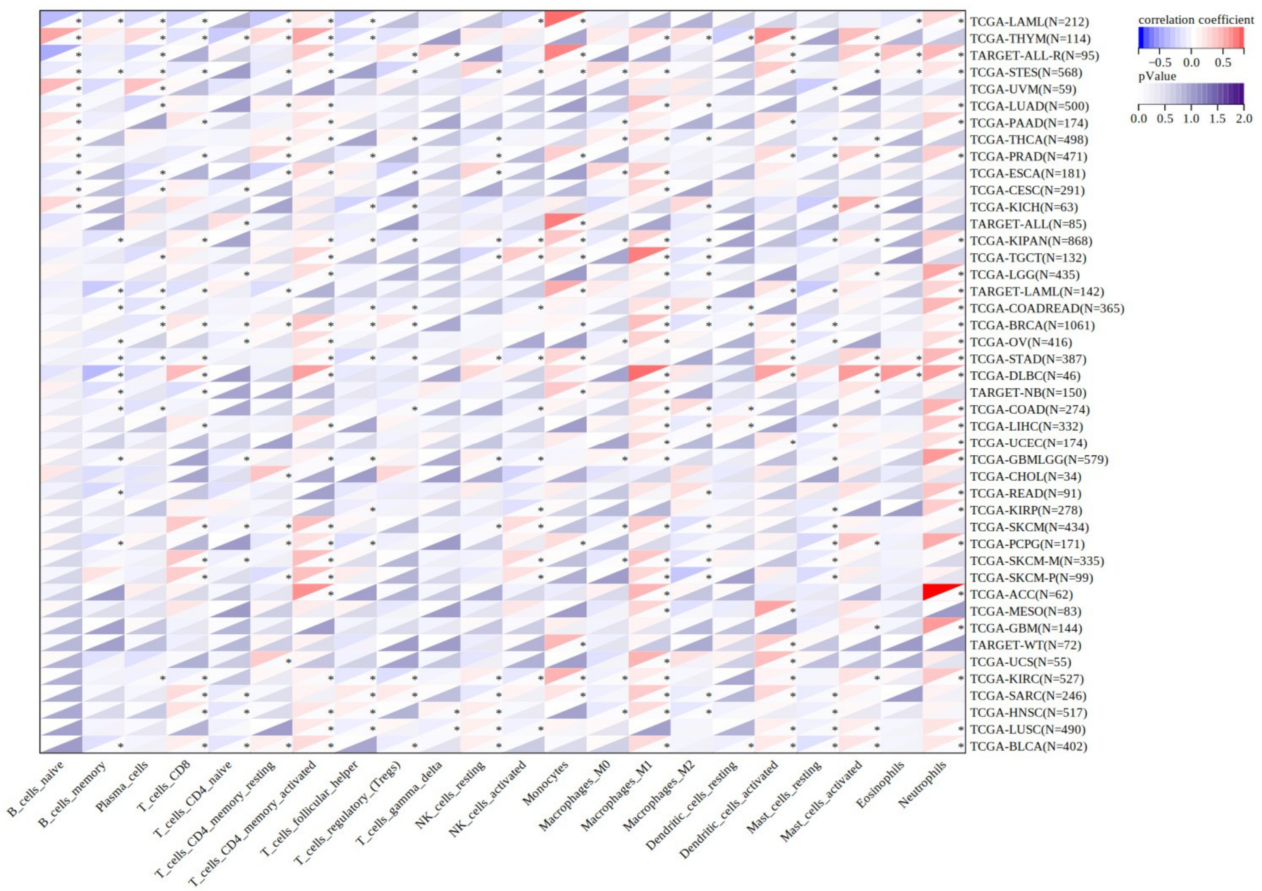

APOBEC3B

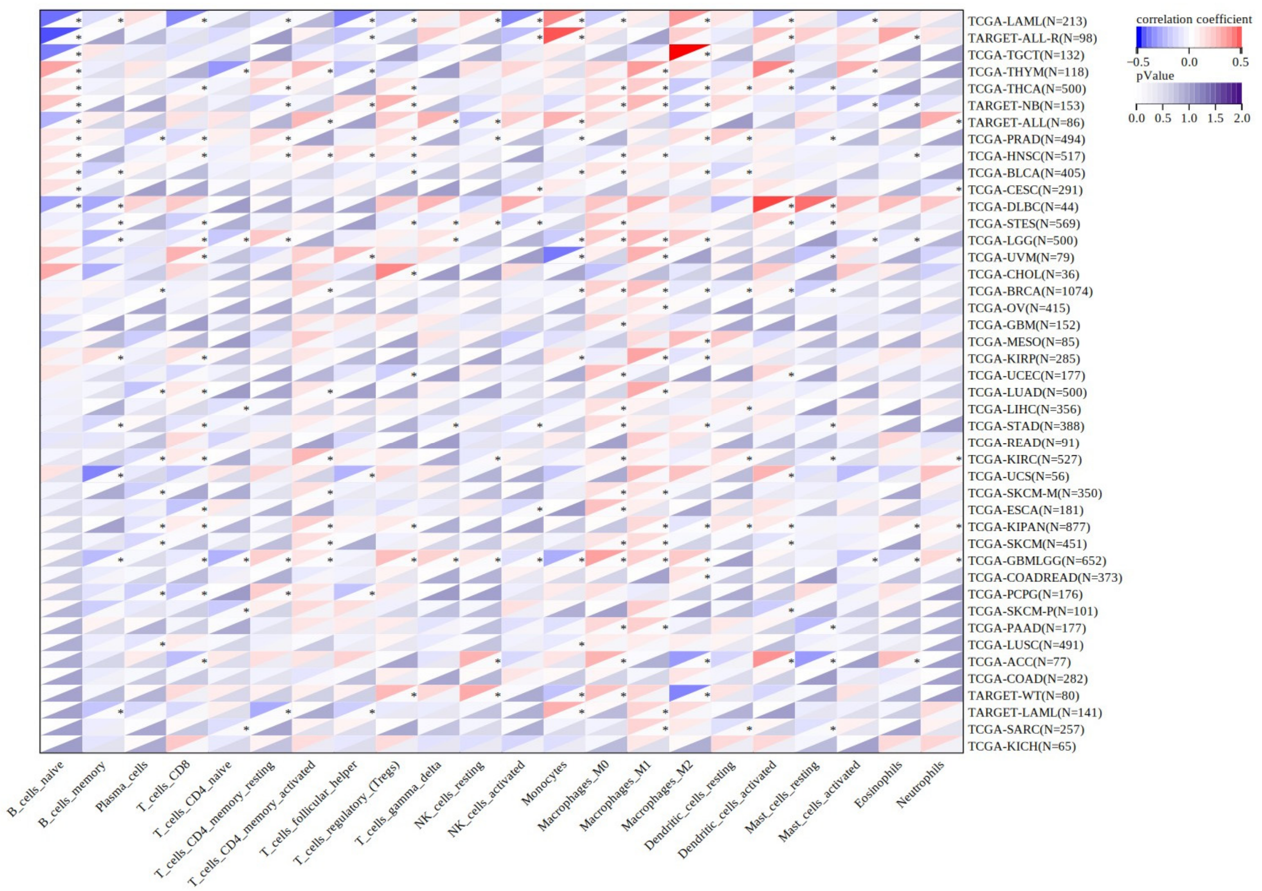

APOBEC3C

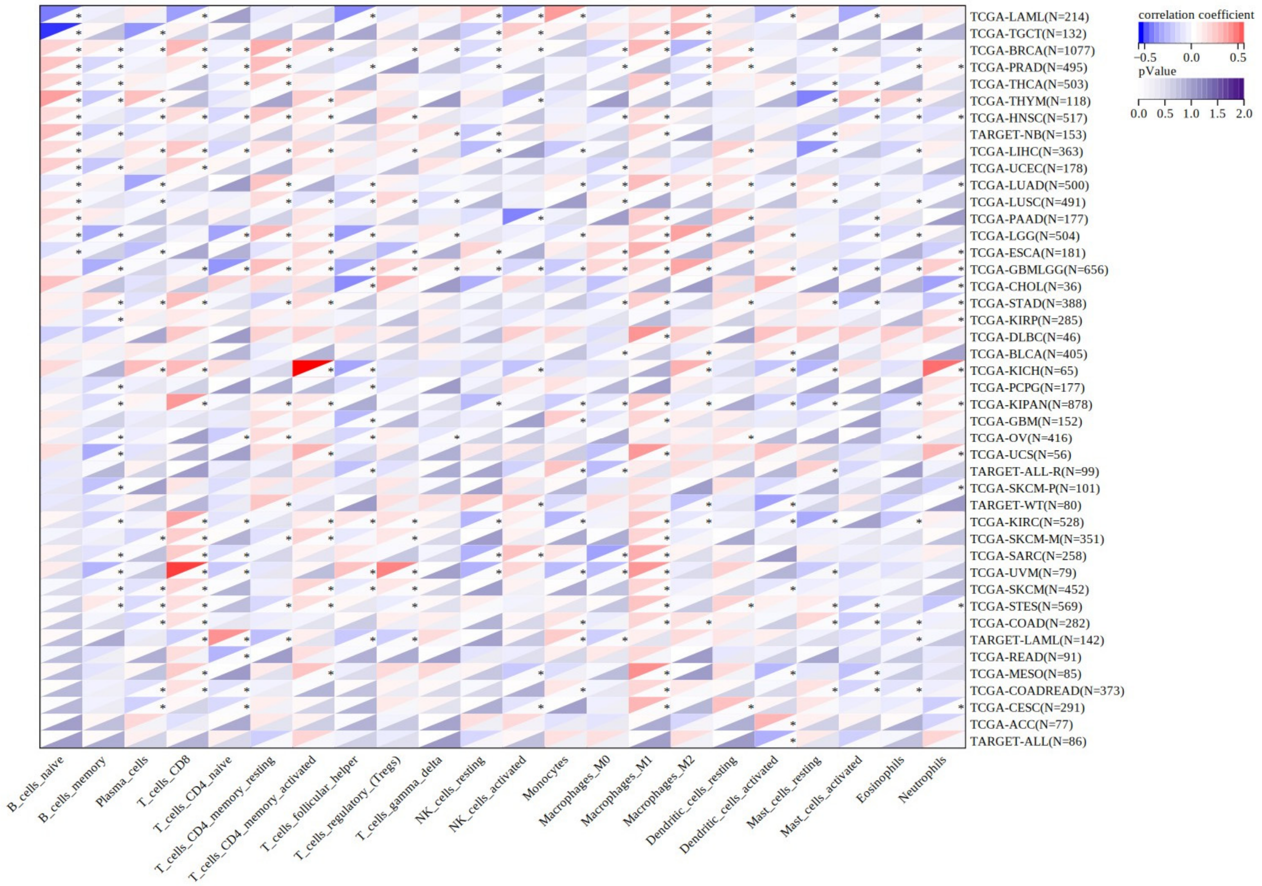

APOBEC3D

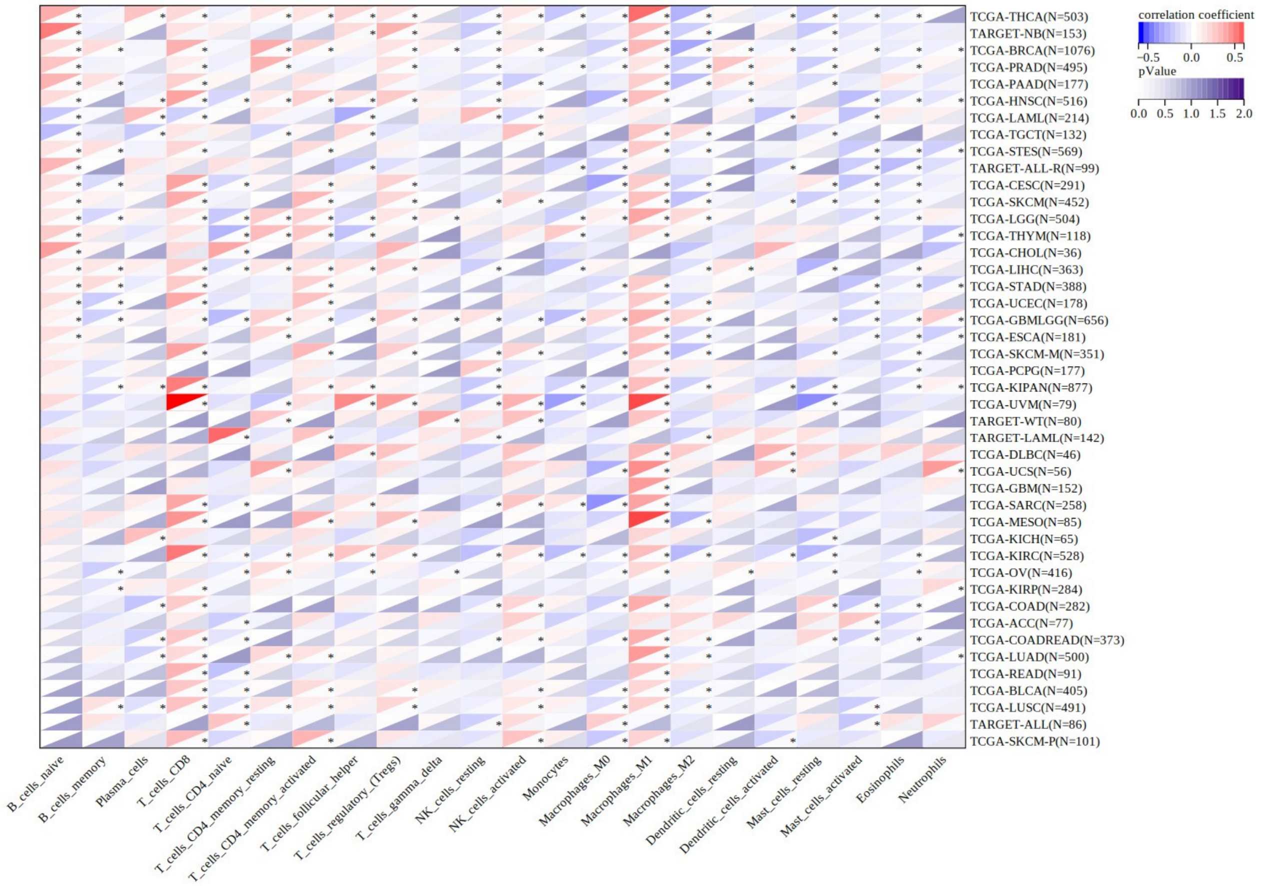

APOBEC3F

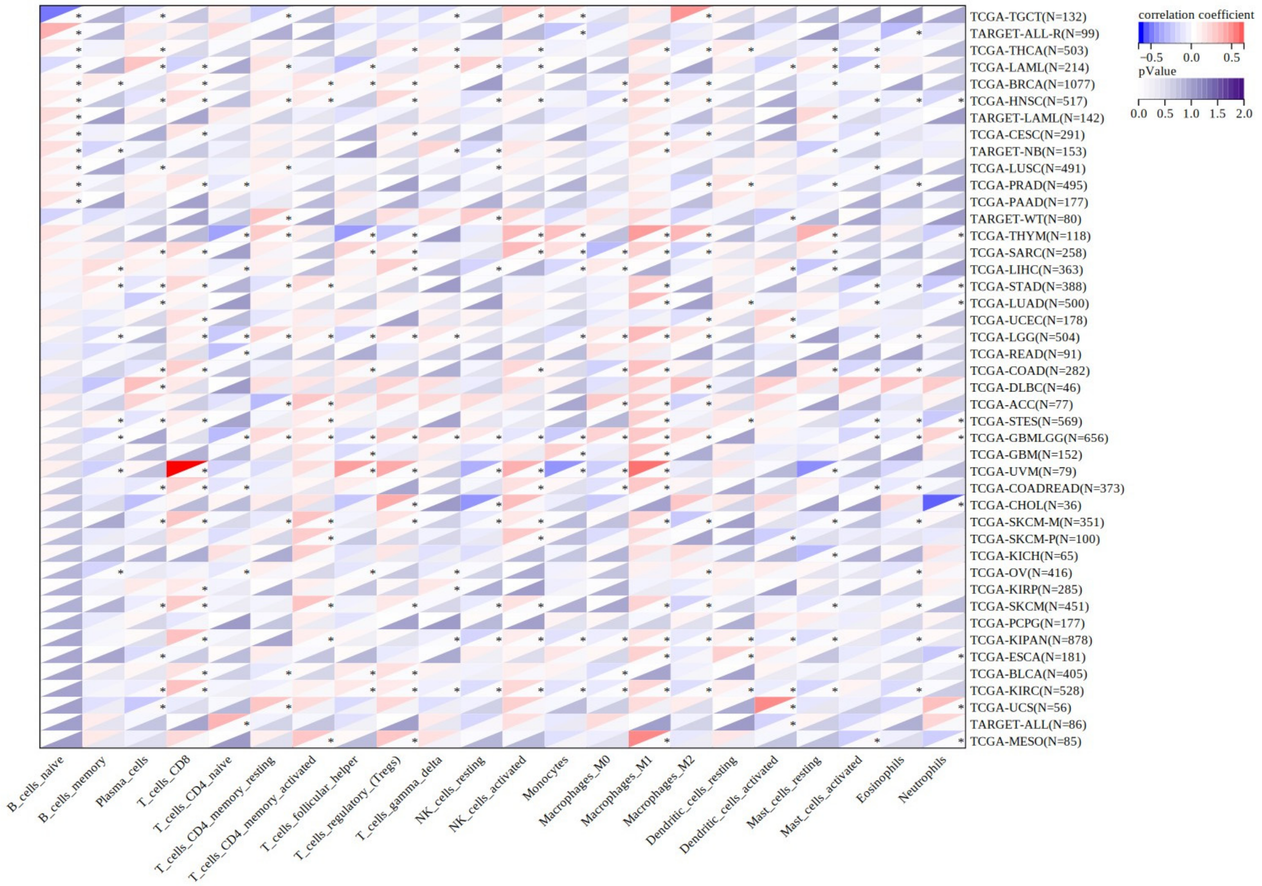

APOBEC3G

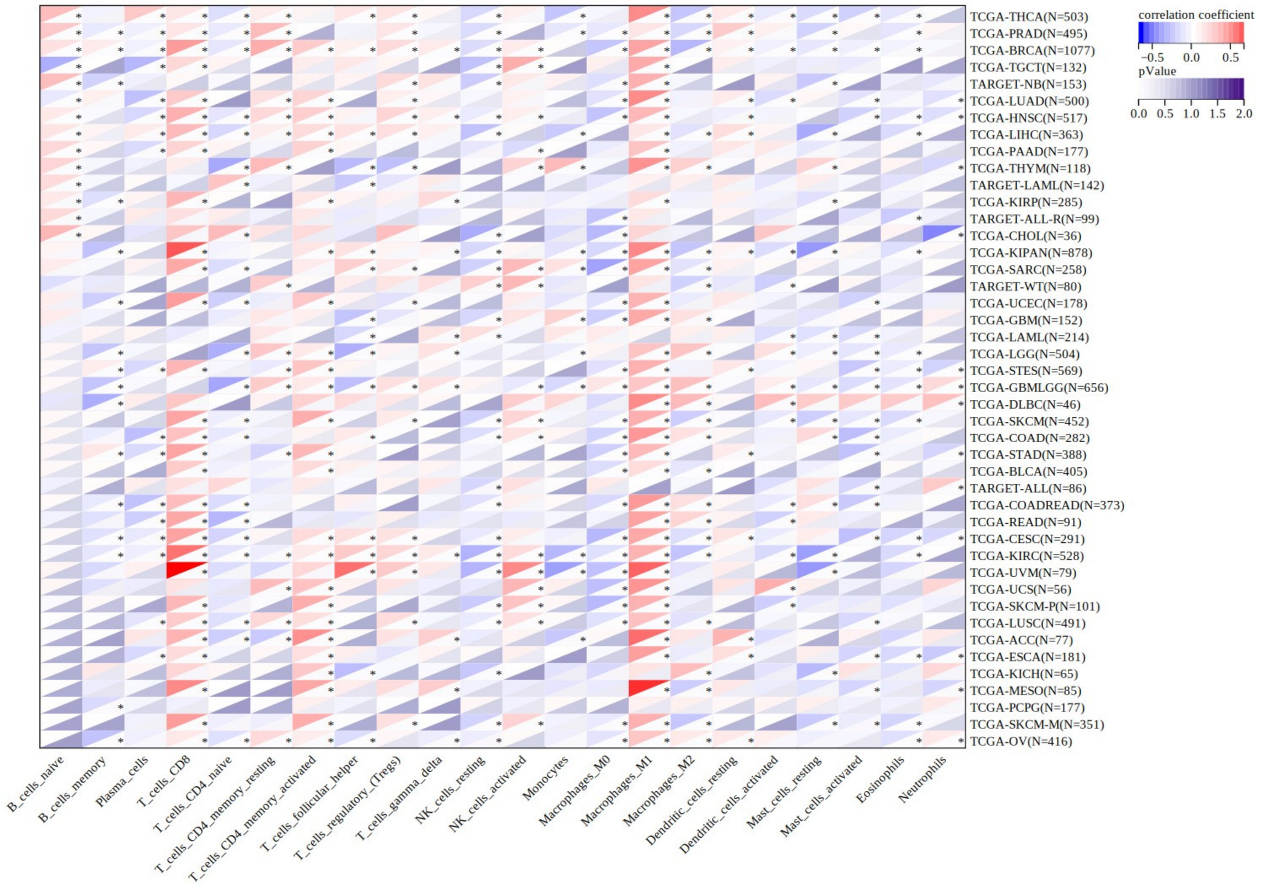

APOBEC3H

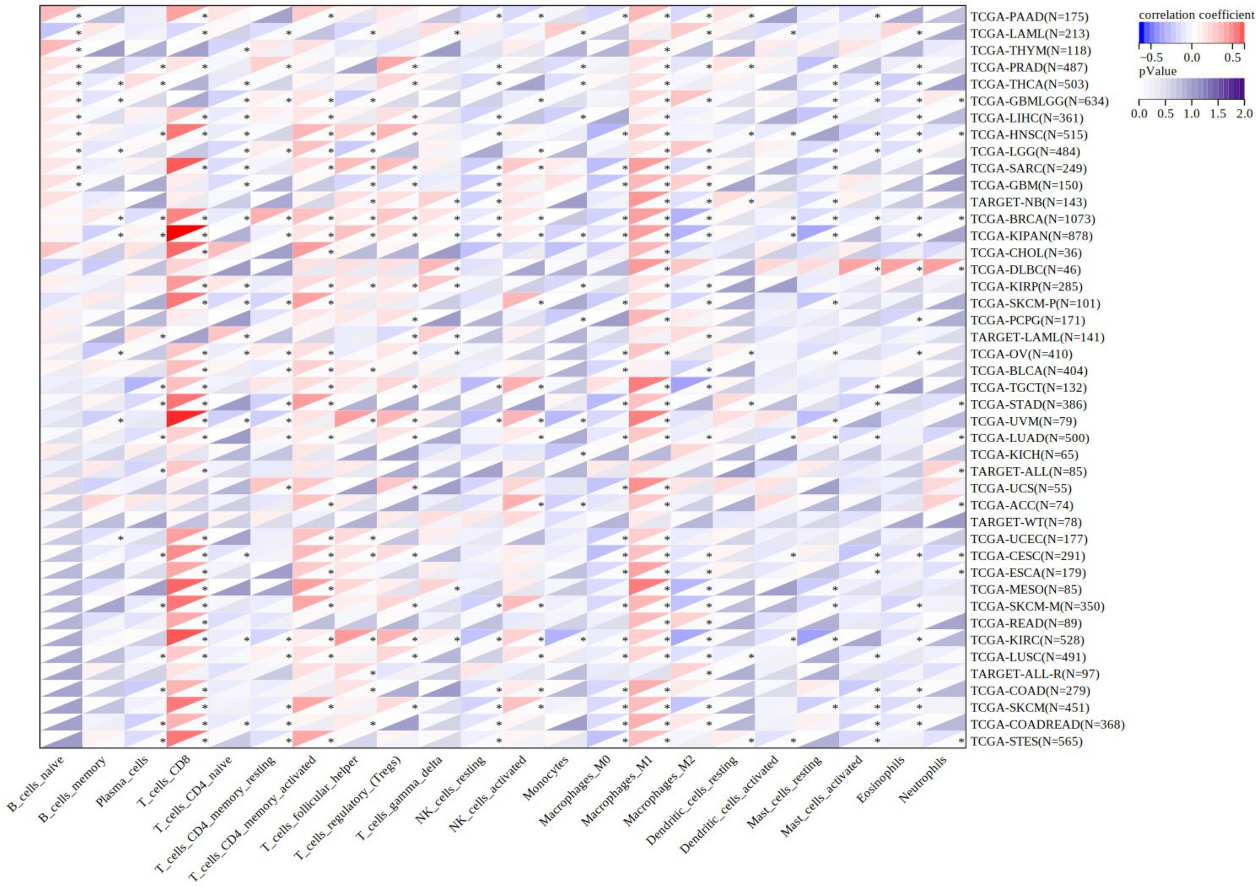

APOBEC4

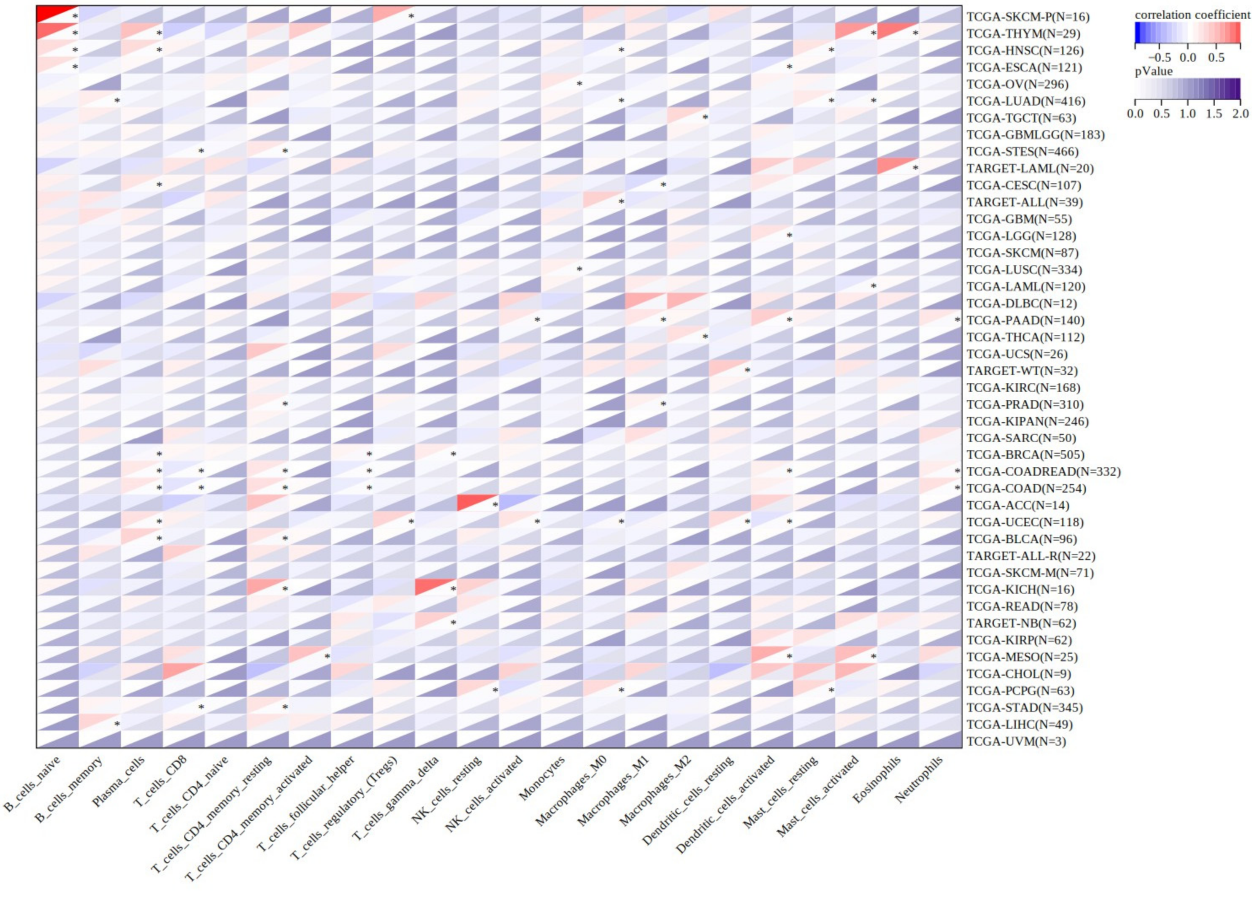
correlation coefficient
−0.5 0.0 0.5
pValue
0.0 0.5 1.0 1.5 2.0
TCGA-SKCM-P(N=16)
TCGA-THYM(N=29)
TCGA-HNSC(N=126)
TCGA-ESCA(N=121)
TCGA-OV(N=296)
TCGA-LUAD(N=416)
TCGA-TGCT(N=63)
TCGA-GBMLGG(N=183)
TCGA-STES(N=466)
TARGET-LAML(N=20)
TCGA-CESC(N=107)
TARGET-ALL(N=39)
TCGA-GBM(N=55)
TCGA-LGG(N=128)
TCGA-SKCM(N=87)
TCGA-LUSC(N=334)
TCGA-LAML(N=120)
TCGA-DLBC(N=12)
TCGA-PAAD(N=140)
TCGA-THCA(N=112)
TCGA-UCS(N=26)
TARGET-WT(N=32)
TCGA-KIRC(N=168)
TCGA-PRAD(N=310)
TCGA-KIPAN(N=246)
TCGA-SARC(N=50)
TCGA-BRCA(N=505)
TCGA-COADREAD(N=332)
TCGA-COAD(N=254)
TCGA-ACC(N=14)
TCGA-UCEC(N=118)
TCGA-BLCA(N=96)
TARGET-ALL-R(N=22)
TCGA-SKCM-M(N=71)
TCGA-KICH(N=16)
TCGA-READ(N=78)
TARGET-NB(N=62)
TCGA-KIRP(N=62)
TCGA-MESO(N=25)
TCGA-CHOL(N=9)
TCGA-PCPG(N=63)
TCGA-STAD(N=345)
TCGA-LIHC(N=49)
TCGA-UVM(N=3)
B_cells_naive
B_cells_memory
Plasma_cells
T_cells_CD8
T_cells_CD4_naive
T_cells_CD4_memory_resting
T_cells_CD4_memory_activated
T_cells_follicular_helper
T_cells_regulatory_(Tregs)
T_cells_gamma_delta
NK_cells_resting
NK_cells_activated
Monocytes
Macrophages_M0
Macrophages_M1
Macrophages_M2
Dendritic_cells_resting
Dendritic_cells_activated
Mast_cells_resting
Mast_cells_activated
Eosinophils
Neutrophils

Table S9. CIBERSORT immune-subset correlations for the six immune-associated enzymes.

Immune subset	Mean r	% positive	n significant
Macrophage M1	+0.23	100	183
$CD8^{+}$ T	+0.16	90	145
$CD4^{+}$ T memory activated	+0.14	99	127
Regulatory T (Treg)	+0.06	82	95
NK activated	+0.05	70	89
B naive	+0.04	80	84
DC resting	+0.04	82	50
γδ T	+0.02	78	45
Neutrophils	+0.02	54	85
$CD4^{+}$ T memory resting	+0.02	66	96
DC activated	+0.00	44	75
T follicular helper	−0.01	47	94
Monocytes	−0.01	44	73
Macrophage M2	−0.03	34	122
Plasma cells	−0.04	20	91
Mast activated	−0.05	19	96
B memory	−0.06	21	80
Mast resting	−0.06	18	105
$CD4^{+}$ T naive	−0.07	11	91
Eosinophils	−0.07	8	87
NK resting	−0.08	21	102
Macrophage M0	−0.09	14	110

Figure S8. Immune-checkpoint co-expression for nine APOBEC family members (APOBEC4 excluded). Significance, nominal $p < 0.05$ (see Table S14).

APOBEC1

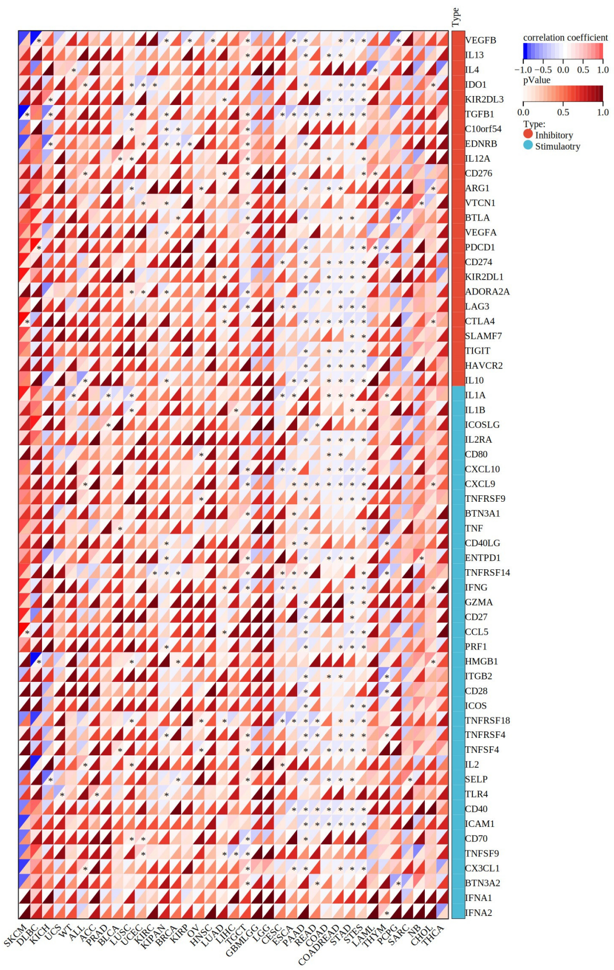

Type
VEGFB
IL13
IL4
IDO1
KIR2DL3
TGFB1
C10orf54
EDNRB
IL12A
CD276
ARG1
VTCN1
BTLA
VEGFA
PDCD1
CD274
KIR2DL1
ADORA2A
LAG3
CTLA4
SLAMF7
TIGIT
HAVCR2
IL10
IL1A
IL1B
ICOSLG
IL2RA
CD80
CXCL10
CXCL9
TNFRSF9
BTN3A1
TNF
CD40LG
ENTPD1
TNFRSF14
IFNG
GZMA
CD27
CCL5
PRF1
HMGB1
ITGB2
CD28
ICOS
TNFRSF18
TNFRSF4
TNFSF4
IL2
SELP
TLR4
CD40
ICAM1
CD70
TNFSF9
CX3CL1
BTN3A2
IFNA1
IFNA2
SKCM
DLBC
KICH
UCS
WT
ALL
ACC
PRAD
BLCA
LUSC
UCEC
KIRC
KIPAN
BRCA
KIRP
OV
HNSC
LUAD
LIHC
TGCT
GBMLGG
LGG
CESC
ESCA
PAAD
READ
COAD
COADREAD
STAD
STES
LAML
THYM
PCPG
SARC
NB
CHOL
THCA
correlation coefficient
−1.0 −0.5 0.0 0.5 1.0
pValue
0.0 0.5 1.0
Type:
Inhibitory
Stimulaotry

APOBEC2

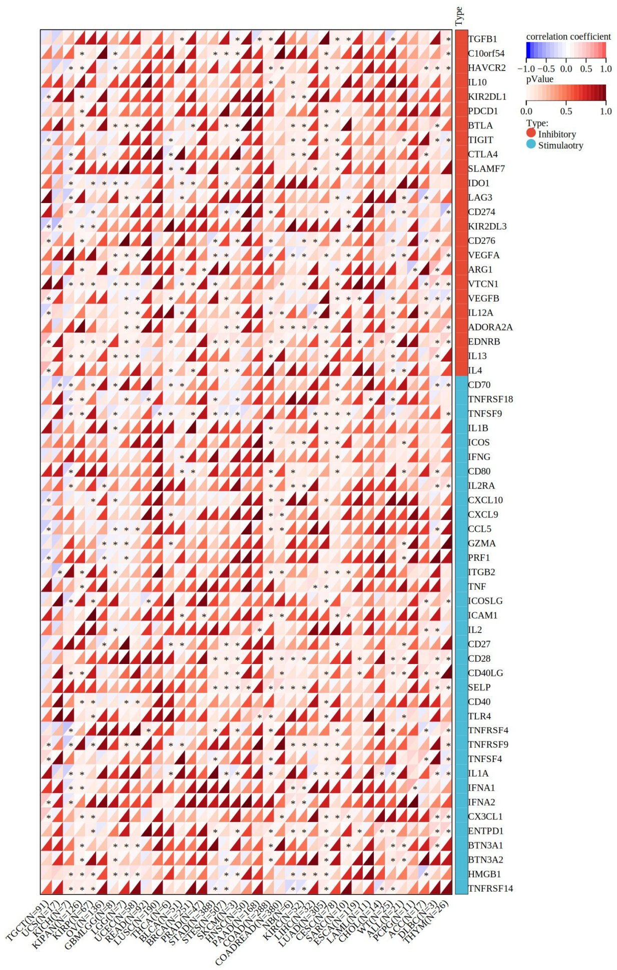
Type
TGFB1
C10orf54
HAVCR2
IL10
KIR2DL1
PDCD1
BTLA
TIGIT
CTLA4
SLAMF7
IDO1
LAG3
CD274
KIR2DL3
CD276
VEGFA
ARG1
VTCN1
VEGFB
IL12A
ADORA2A
EDNRB
IL13
IL4
CD70
TNFRSF18
TNFSF9
IL1B
ICOS
IFNG
CD80
IL2RA
CXCL10
CXCL9
CCL5
GZMA
PRF1
ITGB2
TNF
ICOSLG
ICAM1
IL2
CD27
CD28
CD40LG
SELP
CD40
TLR4
TNFRSF4
TNFRSF9
TNFSF4
IL1A
IFNA1
IFNA2
CX3CL1
ENTPD1
BTN3A1
BTN3A2
HMGB1
TNFRSF14
TGCT(N=91)
UCS(N=17)
KICH(N=7)
KIPAN(N=126)
KIRP(N=67)
OV(N=136)
GBMLGG(N=8)
LGG(N=7)
UCEC(N=58)
READ(N=92)
LUSC(N=190)
THCA(N=6)
BLCA(N=51)
BRCA(N=251)
PRAD(N=47)
STAD(N=388)
STES(N=507)
SKCM(N=3)
HNSC(N=50)
PAAD(N=168)
COAD(N=288)
COADREAD(N=380)
NB(N=6)
KIRC(N=52)
LIHC(N=32)
LUAD(N=305)
CESC(N=78)
SARC(N=10)
ESCA(N=119)
LAML(N=11)
CHOL(N=14)
WT(N=25)
ALL(N=21)
PCPG(N=11)
ACC(N=17)
DLBC(N=3)
THYM(N=26)
correlation coefficient
−1.0 −0.5 0.0 0.5 1.0
pValue
0.0 0.5 1.0
Type:
Inhibitory
Stimulaotry

APOBEC3A

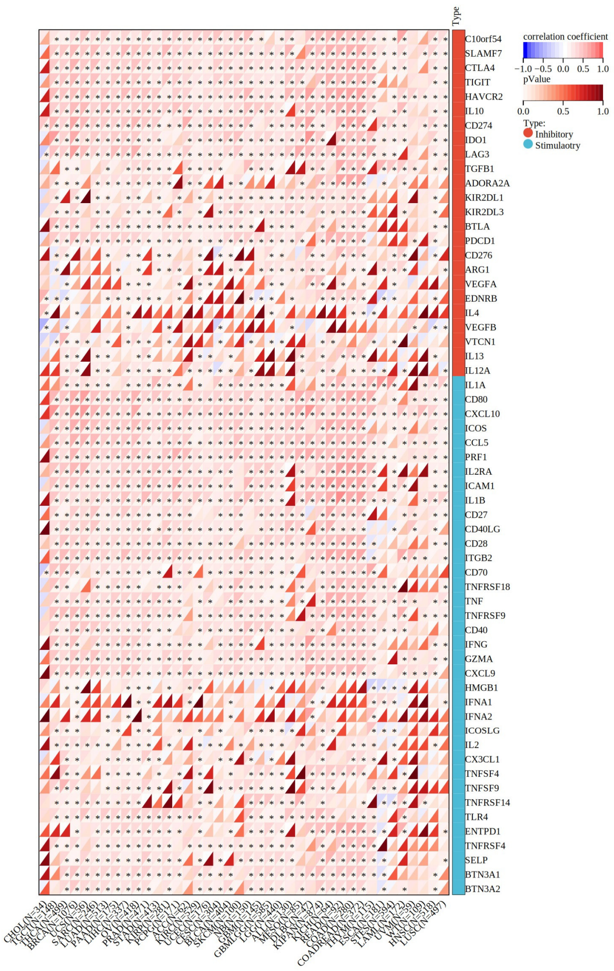
Type
C10orf54
SLAMF7
CTLA4
TIGIT
HAVCR2
IL10
CD274
IDO1
LAG3
TGFB1
ADORA2A
KIR2DL1
KIR2DL3
BTLA
PDCD1
CD276
ARG1
VEGFA
EDNRB
IL4
VEGFB
VTCN1
IL13
IL12A
IL1A
CD80
CXCL10
ICOS
CCL5
PRF1
IL2RA
ICAM1
IL1B
CD27
CD40LG
CD28
ITGB2
CD70
TNFRSF18
TNF
TNFRSF9
CD40
IFNG
GZMA
CXCL9
HMGB1
IFNA1
IFNA2
ICOSLG
IL2
CX3CL1
TNFSF4
TNFSF9
TNFRSF14
TLR4
ENTPD1
TNFRSF4
SELP
BTN3A1
BTN3A2
correlation coefficient
−1.0 −0.5 0.0 0.5 1.0
pValue
0.0 0.5 1.0
Type:
Inhibitory
Stimulaotry

APOBEC3B

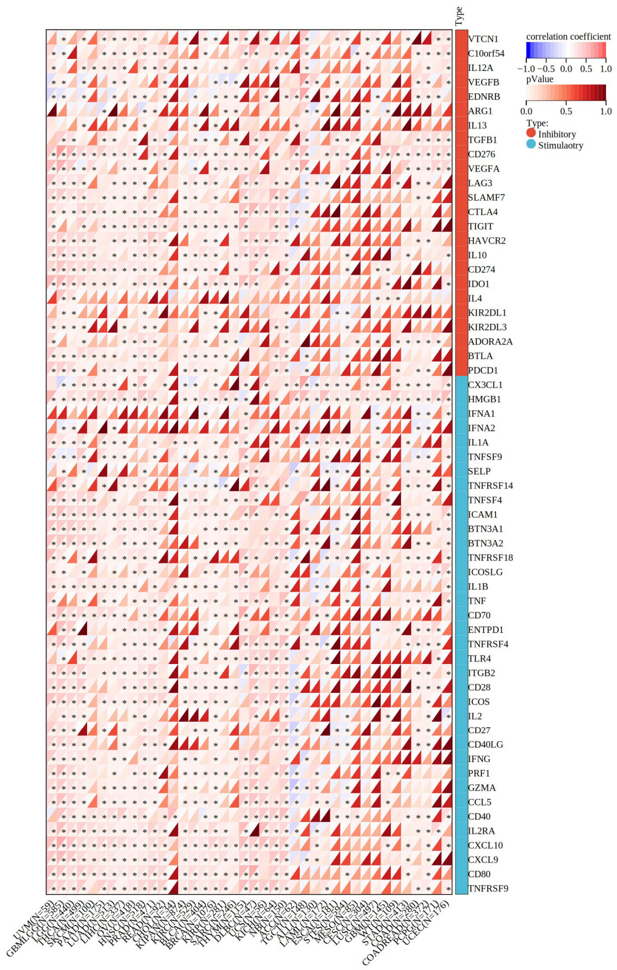
Type
VTCN1
C10orf54
IL12A
VEGFB
EDNRB
ARG1
IL13
TGFB1
CD276
VEGFA
LAG3
SLAMF7
CTLA4
TIGIT
HAVCR2
IL10
CD274
IDO1
IL4
KIR2DL1
KIR2DL3
ADORA2A
BTLA
PDCD1
CX3CL1
HMGB1
IFNA1
IFNA2
IL1A
TNFSF9
SELP
TNFRSF14
TNFSF4
ICAM1
BTN3A1
BTN3A2
TNFRSF18
ICOSLG
IL1B
TNF
CD70
ENTPD1
TNFRSF4
TLR4
ITGB2
CD28
ICOS
IL2
CD27
CD40LG
IFNG
PRF1
GZMA
CCL5
CD40
IL2RA
CXCL10
CXCL9
CD80
TNFRSF9
correlation coefficient
−1.0 −0.5 0.0 0.5 1.0
pValue
0.0 0.5 1.0
Type:
Inhibitory
Stimulaotry

APOBEC3C

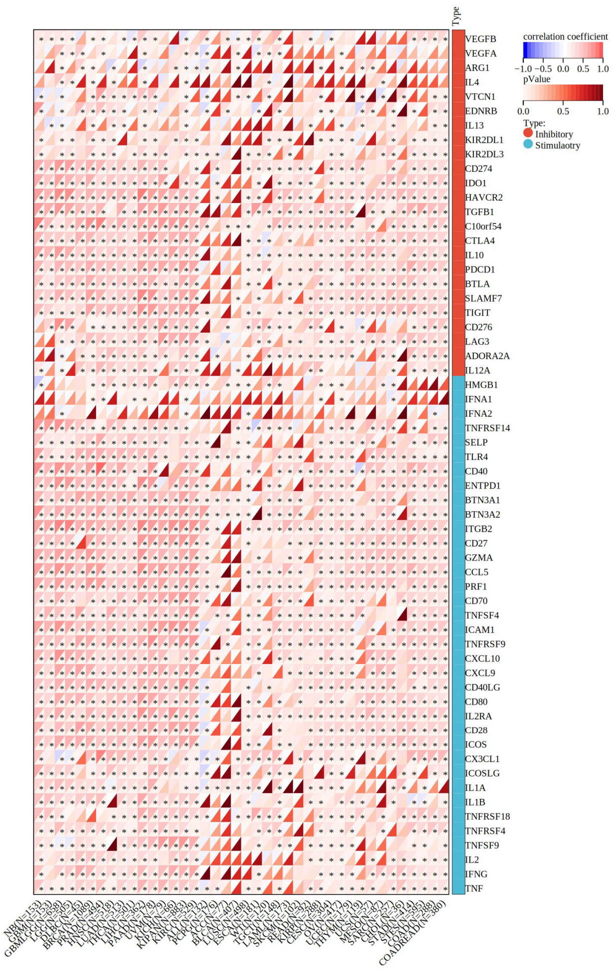

Type
VEGFB
VEGFA
ARG1
IL4
VTCN1
EDNRB
IL13
KIR2DL1
KIR2DL3
CD274
IDO1
HAVCR2
TGFB1
C10orf54
CTLA4
IL10
PDCD1
BTLA
SLAMF7
TIGIT
CD276
LAG3
ADORA2A
IL12A
HMGB1
IFNA1
IFNA2
TNFRSF14
SELP
TLR4
CD40
ENTPD1
BTN3A1
BTN3A2
ITGB2
CD27
GZMA
CCL5
PRF1
CD70
TNFSF4
ICAM1
TNFRSF9
CXCL10
CXCL9
CD40LG
CD80
IL2RA
CD28
ICOS
CX3CL1
ICOSLG
IL1A
IL1B
TNFRSF18
TNFRSF4
TNFSF9
IL2
IFNG
TNF
correlation coefficient
−1.0 −0.5 0.0 0.5 1.0
pValue
0.0 0.5 1.0
Type:
Inhibitory
Stimulaotry
NB(N=153)
GBM(N=153)
GBMLGG(N=658)
LGG(N=505)
DLBC(N=45)
BRCA(N=1089)
PRAD(N=494)
HNSC(N=518)
LUAD(N=513)
THCA(N=501)
LIHC(N=362)
PAAD(N=178)
UVM(N=79)
KICH(N=66)
KIPAN(N=883)
KIRC(N=529)
ALL(N=132)
PCPG(N=176)
ACC(N=77)
BLCA(N=407)
LUSC(N=498)
ESCA(N=181)
WT(N=120)
TGCT(N=148)
LAML(N=173)
SKCM(N=102)
READ(N=92)
KIRP(N=288)
CESC(N=304)
OV(N=417)
UCEC(N=179)
THYM(N=119)
UCS(N=57)
MESO(N=87)
SARC(N=257)
CHOL(N=36)
STAD(N=414)
STES(N=595)
COAD(N=288)
COADREAD(N=380)

APOBEC3D

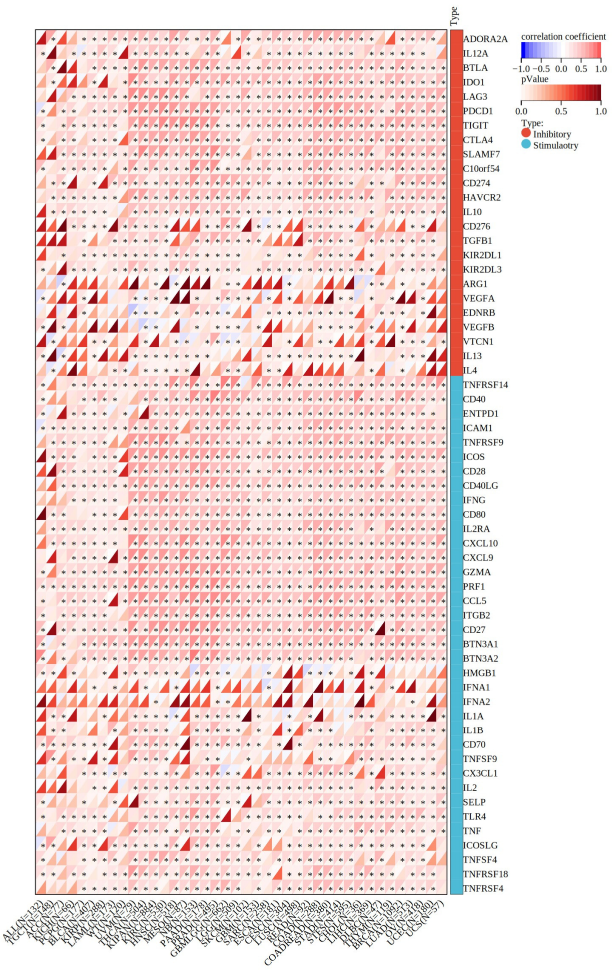
Type
ADORA2A
IL12A
BTLA
IDO1
LAG3
PDCD1
TIGIT
CTLA4
SLAMF7
C10orf54
CD274
HAVCR2
IL10
CD276
TGFB1
KIR2DL1
KIR2DL3
ARG1
VEGFA
EDNRB
VEGFB
VTCN1
IL13
IL4
TNFRSF14
CD40
ENTPD1
ICAM1
TNFRSF9
ICOS
CD28
CD40LG
IFNG
CD80
IL2RA
CXCL10
CXCL9
GZMA
PRF1
CCL5
ITGB2
CD27
BTN3A1
BTN3A2
HMGB1
IFNA1
IFNA2
IL1A
IL1B
CD70
TNFSF9
CX3CL1
IL2
SELP
TLR4
TNF
ICOSLG
TNFSF4
TNFRSF18
TNFRSF4
correlation coefficient
−1.0 −0.5 0.0 0.5 1.0
pValue
0.0 0.5 1.0
Type:
Inhibitory
Stimulaotry

APOBEC3F

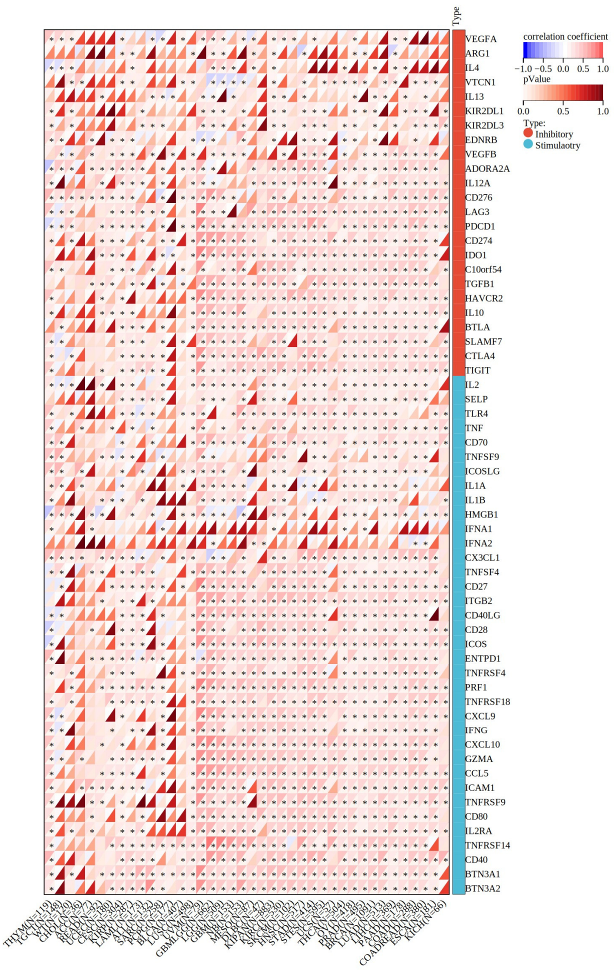
Type
correlation coefficient
−1.0 −0.5 0.0 0.5 1.0
pValue
0.0 0.5 1.0
Type:
Inhibitory
Stimulaotry
VEGFA
ARG1
IL4
VTCN1
IL13
KIR2DL1
KIR2DL3
EDNRB
VEGFB
ADORA2A
IL12A
CD276
LAG3
PDCD1
CD274
IDO1
C10orf54
TGFB1
HAVCR2
IL10
BTLA
SLAMF7
CTLA4
TIGIT
IL2
SELP
TLR4
TNF
CD70
TNFSF9
ICOSLG
IL1A
IL1B
HMGB1
IFNA1
IFNA2
CX3CL1
TNFSF4
CD27
ITGB2
CD40LG
CD28
ICOS
ENTPD1
TNFRSF4
PRF1
TNFRSF18
CXCL9
IFNG
CXCL10
GZMA
CCL5
ICAM1
TNFRSF9
CD80
IL2RA
TNFRSF14
CD40
BTN3A1
BTN3A2

APOBEC3G

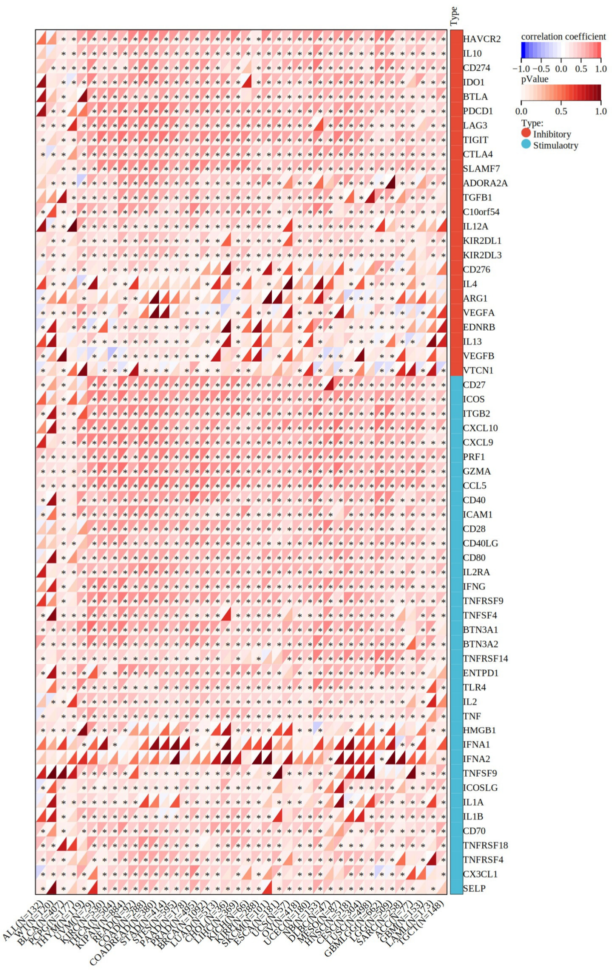
Type
correlation coefficient
−1.0 −0.5 0.0 0.5 1.0
pValue
0.0 0.5 1.0
Type:
Inhibitory
Stimulaotry
HAVCR2
IL10
CD274
IDO1
BTLA
PDCD1
LAG3
TIGIT
CTLA4
SLAMF7
ADORA2A
TGFB1
C10orf54
IL12A
KIR2DL1
KIR2DL3
CD276
IL4
ARG1
VEGFA
EDNRB
IL13
VEGFB
VTCN1
CD27
ICOS
ITGB2
CXCL10
CXCL9
PRF1
GZMA
CCL5
CD40
ICAM1
CD28
CD40LG
CD80
IL2RA
IFNG
TNFRSF9
TNFSF4
BTN3A1
BTN3A2
TNFRSF14
ENTPD1
TLR4
IL2
TNF
HMGB1
IFNA1
IFNA2
TNFSF9
ICOSLG
IL1A
IL1B
CD70
TNFRSF18
TNFRSF4
CX3CL1
SELP

APOBEC3H

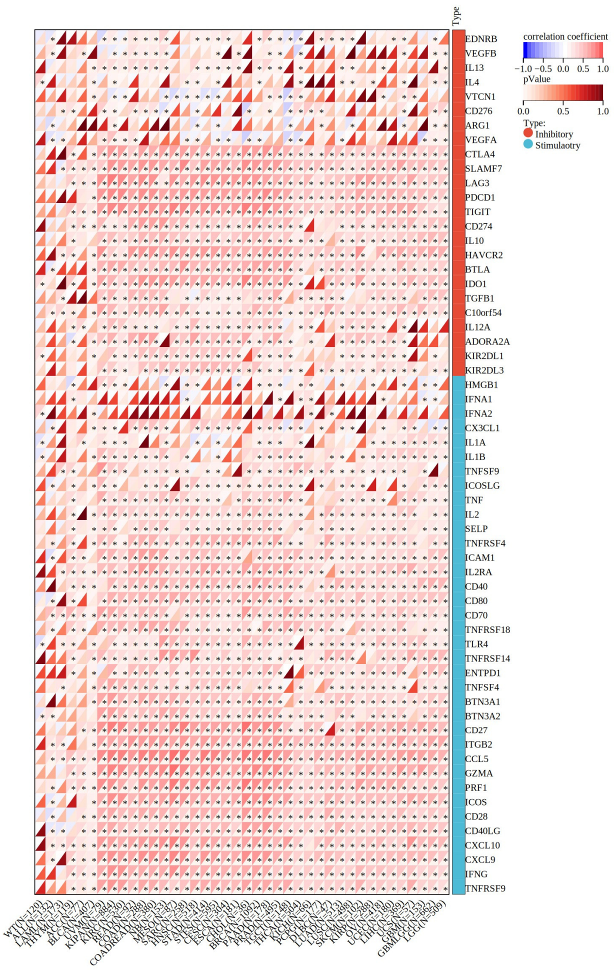
Type
EDNRB
VEGFB
IL13
IL4
VTCN1
CD276
ARG1
VEGFA
CTLA4
SLAMF7
LAG3
PDCD1
TIGIT
CD274
IL10
HAVCR2
BTLA
IDO1
TGFB1
C10orf54
IL12A
ADORA2A
KIR2DL1
KIR2DL3
HMGB1
IFNA1
IFNA2
CX3CL1
IL1A
IL1B
TNFSF9
ICOSLG
TNF
IL2
SELP
TNFRSF4
ICAM1
IL2RA
CD40
CD80
CD70
TNFRSF18
TLR4
TNFRSF14
ENTPD1
TNFSF4
BTN3A1
BTN3A2
CD27
ITGB2
CCL5
GZMA
PRF1
ICOS
CD28
CD40LG
CXCL10
CXCL9
IFNG
TNFRSF9
correlation coefficient
−1.0 −0.5 0.0 0.5 1.0
pValue
0.0 0.5 1.0
Type:
Inhibitory
Stimulaotry
WT(N=120)
ALL(N=132)
LAML(N=173)
THYM(N=119)
ACC(N=77)
BLCA(N=407)
UVM(N=79)
KIPAN(N=884)
KIRC(N=530)
READ(N=92)
COAD(N=288)
COADREAD(N=380)
NB(N=153)
MESO(N=87)
SARC(N=258)
HNSC(N=518)
STAD(N=414)
STES(N=595)
CESC(N=304)
ESCA(N=181)
CHOL(N=36)
BRCA(N=1092)
PAAD(N=178)
PRAD(N=495)
TGCT(N=148)
THCA(N=504)
KICH(N=66)
PCPG(N=177)
DLBC(N=47)
LUAD(N=513)
LUSC(N=498)
SKCM(N=102)
KIRP(N=288)
OV(N=418)
UCEC(N=180)
LIHC(N=369)
UCS(N=57)
GBM(N=153)
GBMLGG(N=662)
LGG(N=509)

Figure S9. The 60 immune-checkpoint genes ranked by mean correlation with the six immune-associated APOBEC3 enzymes. Significance, nominal $p < 0.05$ (see Table S14).

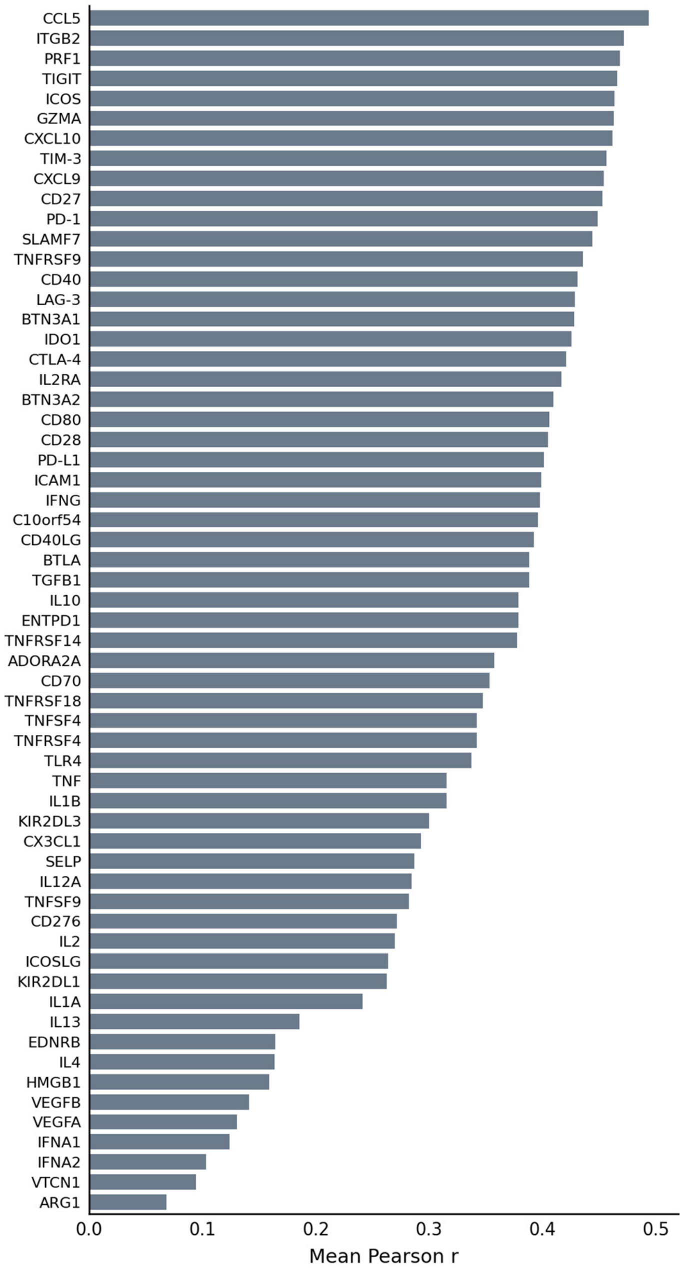

Table S10. APOBEC3–checkpoint co-expression ranked by checkpoint gene.

Checkpoint	Class	Mean r	% positive	n sig
CCL5	Stimulatory	+0.494	100	223
ITGB2	Stimulatory	+0.472	100	214
PRF1	Stimulatory	+0.468	100	226

TIGIT	Inhibitory	+0.466	100	216
ICOS	Stimulatory	+0.464	99	208
GZMA	Stimulatory	+0.463	100	218
CXCL10	Stimulatory	+0.462	100	214
HAVCR2	Inhibitory	+0.457	100	213
CXCL9	Stimulatory	+0.454	100	207
CD27	Stimulatory	+0.453	100	204
PDCD1	Inhibitory	+0.449	99	212
SLAMF7	Inhibitory	+0.444	99	209
TNFRSF9	Stimulatory	+0.436	100	209
CD40	Stimulatory	+0.431	100	202
LAG3	Inhibitory	+0.429	99	213
BTN3A1	Stimulatory	+0.428	100	213
IDO1	Inhibitory	+0.426	99	201
CTLA4	Inhibitory	+0.421	99	211
IL2RA	Stimulatory	+0.417	100	211
BTN3A2	Stimulatory	+0.410	99	205
CD80	Stimulatory	+0.406	99	208
CD28	Stimulatory	+0.405	98	204
CD274	Inhibitory	+0.401	100	204
ICAM1	Stimulatory	+0.399	98	215
IFNG	Stimulatory	+0.398	99	198
C10orf54	Inhibitory	+0.396	100	214
CD40LG	Stimulatory	+0.393	97	207
BTLA	Inhibitory	+0.388	99	197
TGFB1	Inhibitory	+0.388	98	178
IL10	Inhibitory	+0.379	100	206
ENTPD1	Stimulatory	+0.379	98	191
TNFRSF14	Stimulatory	+0.378	98	190
ADORA2A	Inhibitory	+0.357	96	178
CD70	Stimulatory	+0.353	99	191
TNFRSF18	Stimulatory	+0.348	99	192
TNFSF4	Stimulatory	+0.342	99	182
TNFRSF4	Stimulatory	+0.342	100	186
TLR4	Stimulatory	+0.338	99	197
TNF	Stimulatory	+0.315	98	195
IL1B	Stimulatory	+0.315	94	166
KIR2DL3	Inhibitory	+0.300	100	177
CX3CL1	Stimulatory	+0.293	89	167
SELP	Stimulatory	+0.287	99	177
IL12A	Stimulatory	+0.285	94	162
TNFSF9	Stimulatory	+0.282	97	144
CD276	Inhibitory	+0.271	92	145
IL2	Stimulatory	+0.270	97	178
ICOSLG	Stimulatory	+0.264	96	163
KIR2DL1	Inhibitory	+0.263	100	159
IL1A	Stimulatory	+0.241	91	150
IL13	Inhibitory	+0.185	92	109
EDNRB	Inhibitory	+0.164	79	133
IL4	Inhibitory	+0.164	93	70
HMGB1	Stimulatory	+0.159	77	121
VEGFB	Inhibitory	+0.141	80	108
VEGFA	Inhibitory	+0.130	73	106
IFNA1	Stimulatory	+0.124	81	47
IFNA2	Stimulatory	+0.103	63	27
VTCN1	Inhibitory	+0.094	69	115

ARG1	Inhibitory	+0.068	69	80

APOBEC expression and tumor stemness

Table S11. APOBEC–mRNAsi stemness correlations per cancer type: unadjusted Pearson r, purity- and ploidy-adjusted partial r, and adjusted p.

Gene	Cancer	N	r (unadjusted)	r (adjusted)	p (adjusted)
APOBEC1	ACC	65	0.186	0.149	0.244
APOBEC1	BLCA	363	0.056	0.073	0.164
APOBEC1	BRCA	917	0.083	0.073	0.027
APOBEC1	CESC	254	−0.039	−0.076	0.229
APOBEC1	CHOL	34	−0.127	0.035	0.851
APOBEC1	COAD	363	0.197	0.135	0.010
APOBEC1	DLBC	38	−0.299	−0.375	0.024
APOBEC1	ESCA	155	0.258	0.248	0.002
APOBEC1	HNSC	460	0.127	0.086	0.065
APOBEC1	KICH	55	0.040	0.096	0.494
APOBEC1	KIRC	297	0.106	0.115	0.048
APOBEC1	KIRP	244	0.192	0.193	0.003
APOBEC1	LGG	468	−0.024	−0.031	0.503
APOBEC1	LIHC	319	−0.085	−0.040	0.477
APOBEC1	LUAD	451	0.009	0.050	0.289
APOBEC1	LUSC	430	0.111	0.043	0.380
APOBEC1	MESO	71	0.100	0.141	0.247
APOBEC1	OV	179	0.246	0.235	0.002
APOBEC1	PAAD	132	0.175	0.198	0.024
APOBEC1	PCPG	151	0.007	0.010	0.906
APOBEC1	PRAD	426	0.075	0.065	0.183
APOBEC1	READ	122	0.136	0.084	0.364
APOBEC1	SARC	212	−0.133	−0.120	0.083
APOBEC1	SKCM	412	0.064	0.061	0.216
APOBEC1	STAD	321	0.072	0.038	0.497
APOBEC1	TGCT	133	−0.530	−0.472	<0.001
APOBEC1	THCA	416	0.022	0.029	0.558
APOBEC1	THYM	96	0.098	0.163	0.116

APOBEC1	UCEC	463	0.016	0.024	0.600
APOBEC1	UCS	55	−0.209	−0.216	0.121
APOBEC2	ACC	65	0.241	0.187	0.142
APOBEC2	BLCA	363	−0.052	−0.097	0.066
APOBEC2	BRCA	917	−0.051	−0.036	0.271
APOBEC2	CESC	254	−0.053	−0.060	0.346
APOBEC2	CHOL	34	−0.363	−0.369	0.037
APOBEC2	COAD	363	−0.157	−0.120	0.022
APOBEC2	DLBC	38	0.039	0.025	0.885
APOBEC2	ESCA	155	−0.064	0.022	0.783
APOBEC2	GBM	147	0.072	0.000	0.996
APOBEC2	HNSC	460	−0.422	−0.372	<0.001
APOBEC2	KICH	55	0.166	0.141	0.313
APOBEC2	KIRC	297	−0.223	−0.247	<0.001
APOBEC2	KIRP	244	−0.281	−0.299	<0.001
APOBEC2	LAML	43	0.082	0.086	0.591
APOBEC2	LGG	468	0.024	−0.014	0.758
APOBEC2	LIHC	319	0.154	0.121	0.031
APOBEC2	LUAD	451	−0.207	−0.228	<0.001
APOBEC2	LUSC	430	−0.174	−0.209	<0.001
APOBEC2	MESO	71	−0.058	−0.029	0.811
APOBEC2	OV	179	−0.019	−0.159	0.034
APOBEC2	PAAD	132	−0.104	−0.085	0.336
APOBEC2	PCPG	151	−0.300	−0.193	0.018
APOBEC2	PRAD	426	−0.257	−0.216	<0.001
APOBEC2	READ	122	−0.060	−0.057	0.536
APOBEC2	SARC	212	−0.033	−0.028	0.682
APOBEC2	SKCM	412	0.007	−0.015	0.763
APOBEC2	STAD	354	−0.208	−0.179	<0.001
APOBEC2	TGCT	133	−0.581	−0.534	<0.001
APOBEC2	THCA	416	−0.031	−0.033	0.502
APOBEC2	THYM	96	−0.001	−0.111	0.288

APOBEC2	UCEC	463	−0.013	−0.010	0.827
APOBEC2	UCS	55	−0.294	−0.301	0.029
APOBEC2	UVM	76	−0.083	−0.050	0.674
APOBEC3A	ACC	65	0.459	0.398	0.001
APOBEC3A	BLCA	363	−0.016	0.116	0.028
APOBEC3A	BRCA	917	0.322	0.430	<0.001
APOBEC3A	CESC	254	0.071	0.178	0.005
APOBEC3A	CHOL	34	−0.063	−0.073	0.690
APOBEC3A	COAD	363	−0.137	0.020	0.709
APOBEC3A	DLBC	38	−0.105	−0.090	0.600
APOBEC3A	ESCA	155	−0.239	−0.146	0.072
APOBEC3A	GBM	147	−0.317	−0.206	0.013
APOBEC3A	HNSC	460	0.083	0.189	<0.001
APOBEC3A	KICH	55	−0.209	−0.117	0.405
APOBEC3A	KIRC	297	−0.050	0.001	0.991
APOBEC3A	KIRP	244	0.074	0.103	0.110
APOBEC3A	LAML	43	−0.478	−0.514	<0.001
APOBEC3A	LGG	468	−0.266	−0.251	<0.001
APOBEC3A	LIHC	319	0.144	0.275	<0.001
APOBEC3A	LUAD	451	0.040	0.192	<0.001
APOBEC3A	LUSC	430	−0.076	0.085	0.080
APOBEC3A	MESO	71	0.135	0.254	0.035
APOBEC3A	OV	179	0.059	0.269	<0.001
APOBEC3A	PAAD	132	−0.028	0.003	0.969
APOBEC3A	PCPG	151	−0.368	−0.232	0.004
APOBEC3A	PRAD	426	−0.150	−0.111	0.022
APOBEC3A	READ	122	−0.087	0.045	0.625
APOBEC3A	SARC	212	0.161	0.188	0.006
APOBEC3A	SKCM	412	0.113	0.234	<0.001
APOBEC3A	STAD	354	−0.095	0.026	0.623
APOBEC3A	TGCT	133	0.350	0.268	0.002
APOBEC3A	THCA	416	−0.217	−0.176	<0.001

APOBEC3A	THYM	96	0.087	0.128	0.219
APOBEC3A	UCEC	463	0.178	0.196	<0.001
APOBEC3A	UCS	55	−0.138	−0.146	0.296
APOBEC3A	UVM	76	0.068	0.188	0.108
APOBEC3B	ACC	65	0.520	0.457	<0.001
APOBEC3B	BLCA	363	0.081	0.121	0.021
APOBEC3B	BRCA	917	0.385	0.404	<0.001
APOBEC3B	CESC	254	0.203	0.178	0.005
APOBEC3B	CHOL	34	0.015	0.105	0.568
APOBEC3B	COAD	363	0.030	0.037	0.489
APOBEC3B	DLBC	38	−0.262	−0.204	0.233
APOBEC3B	ESCA	155	0.015	0.007	0.929
APOBEC3B	GBM	147	−0.023	−0.099	0.236
APOBEC3B	HNSC	460	0.166	0.161	<0.001
APOBEC3B	KICH	55	−0.032	−0.135	0.336
APOBEC3B	KIRC	297	0.377	0.406	<0.001
APOBEC3B	KIRP	244	0.198	0.203	0.001
APOBEC3B	LAML	43	0.278	0.292	0.064
APOBEC3B	LGG	468	−0.172	−0.236	<0.001
APOBEC3B	LIHC	319	0.168	0.181	0.001
APOBEC3B	LUAD	451	0.243	0.301	<0.001
APOBEC3B	LUSC	430	0.200	0.162	<0.001
APOBEC3B	MESO	71	0.393	0.328	0.006
APOBEC3B	OV	179	0.162	0.182	0.015
APOBEC3B	PAAD	132	0.334	0.362	<0.001
APOBEC3B	PCPG	151	−0.179	−0.196	0.016
APOBEC3B	PRAD	426	0.201	0.212	<0.001
APOBEC3B	READ	122	−0.059	−0.028	0.759
APOBEC3B	SARC	212	0.268	0.255	<0.001
APOBEC3B	SKCM	412	0.199	0.229	<0.001
APOBEC3B	STAD	354	0.132	0.088	0.098
APOBEC3B	TGCT	133	−0.470	−0.440	<0.001

APOBEC3B	THCA	416	−0.055	−0.025	0.608
APOBEC3B	THYM	96	0.218	0.236	0.022
APOBEC3B	UCEC	463	0.278	0.249	<0.001
APOBEC3B	UCS	55	−0.077	−0.072	0.607
APOBEC3B	UVM	76	0.445	0.492	<0.001
APOBEC3C	ACC	65	0.082	0.194	0.128
APOBEC3C	BLCA	363	−0.097	−0.103	0.050
APOBEC3C	BRCA	917	−0.210	0.012	0.716
APOBEC3C	CESC	254	−0.159	−0.065	0.307
APOBEC3C	CHOL	34	−0.404	−0.327	0.067
APOBEC3C	COAD	363	−0.333	−0.236	<0.001
APOBEC3C	DLBC	38	−0.361	−0.318	0.059
APOBEC3C	ESCA	155	−0.313	−0.205	0.011
APOBEC3C	GBM	147	−0.622	−0.426	<0.001
APOBEC3C	HNSC	460	−0.073	−0.032	0.492
APOBEC3C	KICH	55	−0.497	−0.352	0.010
APOBEC3C	KIRC	297	−0.295	−0.207	<0.001
APOBEC3C	KIRP	244	−0.281	−0.281	<0.001
APOBEC3C	LAML	43	−0.015	−0.004	0.980
APOBEC3C	LGG	468	−0.581	−0.553	<0.001
APOBEC3C	LIHC	319	−0.418	−0.278	<0.001
APOBEC3C	LUAD	451	−0.280	−0.152	0.001
APOBEC3C	LUSC	430	−0.177	−0.084	0.082
APOBEC3C	MESO	71	0.141	0.145	0.233
APOBEC3C	OV	179	−0.142	−0.049	0.517
APOBEC3C	PAAD	132	−0.305	−0.289	<0.001
APOBEC3C	PCPG	151	−0.054	−0.027	0.745
APOBEC3C	PRAD	426	−0.674	−0.555	<0.001
APOBEC3C	READ	122	−0.271	−0.162	0.077
APOBEC3C	SARC	212	−0.071	−0.058	0.400
APOBEC3C	SKCM	412	−0.171	−0.124	0.012
APOBEC3C	STAD	354	−0.380	−0.205	<0.001

APOBEC3C	TGCT	133	−0.075	−0.114	0.195
APOBEC3C	THCA	416	−0.052	0.002	0.966
APOBEC3C	THYM	96	0.287	0.190	0.067
APOBEC3C	UCEC	463	−0.231	−0.142	0.002
APOBEC3C	UCS	55	−0.168	−0.178	0.201
APOBEC3C	UVM	76	−0.147	−0.069	0.556
APOBEC3D	ACC	65	−0.164	−0.045	0.727
APOBEC3D	BLCA	363	−0.072	−0.000	0.995
APOBEC3D	BRCA	917	−0.148	0.057	0.083
APOBEC3D	CESC	254	−0.158	−0.056	0.380
APOBEC3D	CHOL	34	−0.176	−0.021	0.907
APOBEC3D	COAD	363	−0.282	−0.139	0.008
APOBEC3D	DLBC	38	−0.444	−0.400	0.016
APOBEC3D	ESCA	155	−0.196	−0.057	0.483
APOBEC3D	GBM	147	−0.522	−0.415	<0.001
APOBEC3D	HNSC	460	0.048	0.139	0.003
APOBEC3D	KICH	55	−0.255	−0.206	0.138
APOBEC3D	KIRC	297	−0.127	−0.013	0.829
APOBEC3D	KIRP	244	−0.183	−0.177	0.006
APOBEC3D	LAML	43	−0.231	−0.219	0.169
APOBEC3D	LGG	468	−0.585	−0.552	<0.001
APOBEC3D	LIHC	319	−0.168	0.005	0.935
APOBEC3D	LUAD	451	−0.119	0.050	0.287
APOBEC3D	LUSC	430	−0.137	−0.018	0.713
APOBEC3D	MESO	71	0.153	0.242	0.045
APOBEC3D	OV	179	−0.022	0.101	0.179
APOBEC3D	PAAD	132	0.042	0.046	0.605
APOBEC3D	PCPG	151	−0.302	−0.140	0.087
APOBEC3D	PRAD	426	−0.556	−0.421	<0.001
APOBEC3D	READ	122	−0.259	−0.151	0.100
APOBEC3D	SARC	212	−0.041	−0.013	0.847
APOBEC3D	SKCM	412	−0.210	−0.127	0.010

APOBEC3D	STAD	354	−0.238	−0.051	0.341
APOBEC3D	TGCT	133	0.359	0.341	<0.001
APOBEC3D	THCA	416	−0.246	−0.195	<0.001
APOBEC3D	THYM	96	0.206	0.129	0.216
APOBEC3D	UCEC	463	−0.086	0.035	0.460
APOBEC3D	UCS	55	−0.139	−0.161	0.250
APOBEC3D	UVM	76	0.102	0.210	0.072
APOBEC3F	ACC	65	−0.259	−0.228	0.073
APOBEC3F	BLCA	363	0.042	0.021	0.689
APOBEC3F	BRCA	917	−0.051	0.081	0.014
APOBEC3F	CESC	254	−0.110	−0.068	0.281
APOBEC3F	CHOL	34	−0.155	−0.162	0.375
APOBEC3F	COAD	363	−0.265	−0.134	0.011
APOBEC3F	DLBC	38	−0.405	−0.382	0.021
APOBEC3F	ESCA	155	−0.229	−0.190	0.018
APOBEC3F	GBM	147	−0.502	−0.364	<0.001
APOBEC3F	HNSC	460	−0.051	−0.019	0.688
APOBEC3F	KICH	55	−0.253	−0.250	0.071
APOBEC3F	KIRC	297	−0.199	−0.136	0.020
APOBEC3F	KIRP	244	0.021	0.026	0.690
APOBEC3F	LAML	43	−0.036	−0.045	0.781
APOBEC3F	LGG	468	−0.663	−0.621	<0.001
APOBEC3F	LIHC	319	−0.115	−0.042	0.452
APOBEC3F	LUAD	451	−0.145	−0.021	0.663
APOBEC3F	LUSC	430	−0.104	−0.075	0.120
APOBEC3F	MESO	71	0.233	0.266	0.027
APOBEC3F	OV	179	0.071	0.048	0.527
APOBEC3F	PAAD	132	−0.022	−0.011	0.903
APOBEC3F	PCPG	151	−0.123	0.004	0.963
APOBEC3F	PRAD	426	−0.322	−0.224	<0.001
APOBEC3F	READ	122	−0.105	−0.005	0.961
APOBEC3F	SARC	212	−0.095	−0.079	0.254

APOBEC3F	SKCM	412	−0.289	−0.227	<0.001
APOBEC3F	STAD	354	−0.230	−0.095	0.075
APOBEC3F	TGCT	133	0.003	0.022	0.799
APOBEC3F	THCA	416	−0.064	−0.005	0.925
APOBEC3F	THYM	96	−0.039	−0.045	0.667
APOBEC3F	UCEC	463	−0.153	−0.123	0.008
APOBEC3F	UCS	55	0.086	0.093	0.507
APOBEC3F	UVM	76	−0.020	0.084	0.476
APOBEC3G	ACC	65	−0.322	−0.161	0.208
APOBEC3G	BLCA	363	−0.081	−0.033	0.532
APOBEC3G	BRCA	917	−0.126	0.111	<0.001
APOBEC3G	CESC	254	−0.188	−0.087	0.169
APOBEC3G	CHOL	34	−0.229	−0.114	0.533
APOBEC3G	COAD	363	−0.309	−0.108	0.040
APOBEC3G	DLBC	38	−0.273	−0.221	0.196
APOBEC3G	ESCA	155	−0.381	−0.247	0.002
APOBEC3G	GBM	147	−0.394	−0.201	0.016
APOBEC3G	HNSC	460	−0.076	0.023	0.616
APOBEC3G	KICH	55	−0.441	−0.334	0.015
APOBEC3G	KIRC	297	−0.088	0.028	0.630
APOBEC3G	KIRP	244	−0.140	−0.140	0.029
APOBEC3G	LAML	43	−0.051	−0.053	0.742
APOBEC3G	LGG	468	−0.670	−0.624	<0.001
APOBEC3G	LIHC	319	−0.170	0.025	0.651
APOBEC3G	LUAD	451	−0.116	0.091	0.055
APOBEC3G	LUSC	430	−0.211	−0.068	0.158
APOBEC3G	MESO	71	0.240	0.345	0.004
APOBEC3G	OV	179	0.046	0.157	0.037
APOBEC3G	PAAD	132	−0.236	−0.208	0.017
APOBEC3G	PCPG	151	−0.158	−0.044	0.592
APOBEC3G	PRAD	426	−0.597	−0.469	<0.001
APOBEC3G	READ	122	−0.367	−0.225	0.014

APOBEC3G	SARC	212	0.002	0.040	0.566
APOBEC3G	SKCM	412	−0.300	−0.233	<0.001
APOBEC3G	STAD	354	−0.258	−0.007	0.897
APOBEC3G	TGCT	133	0.267	0.181	0.039
APOBEC3G	THCA	416	−0.248	−0.197	<0.001
APOBEC3G	THYM	96	−0.195	−0.266	0.010
APOBEC3G	UCEC	463	−0.066	0.061	0.188
APOBEC3G	UCS	55	0.012	0.000	0.999
APOBEC3G	UVM	76	0.008	0.142	0.228
APOBEC3H	ACC	65	−0.187	−0.027	0.835
APOBEC3H	BLCA	363	−0.095	−0.038	0.471
APOBEC3H	BRCA	917	−0.014	0.229	<0.001
APOBEC3H	CESC	254	−0.098	0.040	0.532
APOBEC3H	CHOL	34	0.058	0.416	0.018
APOBEC3H	COAD	363	−0.232	−0.032	0.543
APOBEC3H	DLBC	38	−0.350	−0.292	0.084
APOBEC3H	ESCA	155	−0.320	−0.127	0.118
APOBEC3H	GBM	147	−0.338	−0.047	0.576
APOBEC3H	HNSC	460	0.049	0.147	0.002
APOBEC3H	KICH	55	−0.180	−0.072	0.609
APOBEC3H	KIRC	297	−0.106	0.007	0.903
APOBEC3H	KIRP	244	−0.096	−0.091	0.158
APOBEC3H	LAML	43	0.071	0.088	0.583
APOBEC3H	LGG	468	−0.547	−0.522	<0.001
APOBEC3H	LIHC	319	−0.202	−0.027	0.635
APOBEC3H	LUAD	451	−0.194	−0.046	0.328
APOBEC3H	LUSC	430	−0.150	−0.018	0.715
APOBEC3H	MESO	71	0.108	0.286	0.017
APOBEC3H	OV	179	0.024	0.249	<0.001
APOBEC3H	PAAD	132	−0.228	−0.212	0.016
APOBEC3H	PCPG	151	−0.464	−0.196	0.016
APOBEC3H	PRAD	426	−0.266	−0.181	<0.001

APOBEC3H	READ	122	−0.291	−0.179	0.050
APOBEC3H	SARC	212	0.015	0.069	0.322
APOBEC3H	SKCM	412	−0.154	−0.038	0.448
APOBEC3H	STAD	354	−0.155	0.075	0.162
APOBEC3H	TGCT	133	0.485	0.422	<0.001
APOBEC3H	THCA	416	−0.203	−0.176	<0.001
APOBEC3H	THYM	96	0.212	0.142	0.172
APOBEC3H	UCEC	463	−0.117	0.032	0.499
APOBEC3H	UCS	55	−0.001	0.002	0.986
APOBEC3H	UVM	76	−0.054	0.056	0.635
APOBEC4	ACC	65	−0.140	−0.167	0.192
APOBEC4	BLCA	363	0.063	0.025	0.641
APOBEC4	BRCA	917	−0.137	−0.144	<0.001
APOBEC4	CESC	254	−0.126	−0.167	0.008
APOBEC4	CHOL	34	−0.266	−0.193	0.291
APOBEC4	COAD	363	0.113	0.050	0.341
APOBEC4	DLBC	38	−0.265	−0.247	0.147
APOBEC4	ESCA	155	0.094	0.103	0.204
APOBEC4	GBM	147	−0.033	−0.088	0.294
APOBEC4	HNSC	460	0.024	0.034	0.469
APOBEC4	KICH	55	0.312	0.274	0.047
APOBEC4	KIRC	297	0.009	0.026	0.653
APOBEC4	KIRP	244	−0.063	−0.063	0.326
APOBEC4	LAML	43	0.294	0.280	0.076
APOBEC4	LGG	468	−0.176	−0.168	<0.001
APOBEC4	LIHC	319	0.054	0.080	0.155
APOBEC4	LUAD	451	−0.357	−0.318	<0.001
APOBEC4	LUSC	430	−0.247	−0.158	0.001
APOBEC4	MESO	71	−0.293	−0.303	0.011
APOBEC4	OV	179	0.065	−0.050	0.506
APOBEC4	PAAD	132	0.005	−0.011	0.898
APOBEC4	PCPG	151	0.088	0.052	0.532

APOBEC4	PRAD	426	−0.012	−0.002	0.961
APOBEC4	READ	122	0.006	−0.030	0.742
APOBEC4	SARC	212	0.076	0.070	0.314
APOBEC4	SKCM	412	−0.125	−0.127	0.010
APOBEC4	STAD	354	0.030	−0.003	0.955
APOBEC4	TGCT	133	−0.376	−0.345	<0.001
APOBEC4	THCA	416	−0.116	−0.122	0.013
APOBEC4	THYM	96	0.059	0.080	0.445
APOBEC4	UCEC	463	−0.382	−0.332	<0.001
APOBEC4	UCS	55	−0.110	−0.154	0.271
APOBEC4	UVM	76	0.072	0.041	0.728

Mutation status versus expression

Figure S10. APOBEC gene expression by somatic mutation status (mutant versus wild-type).

APOBEC1

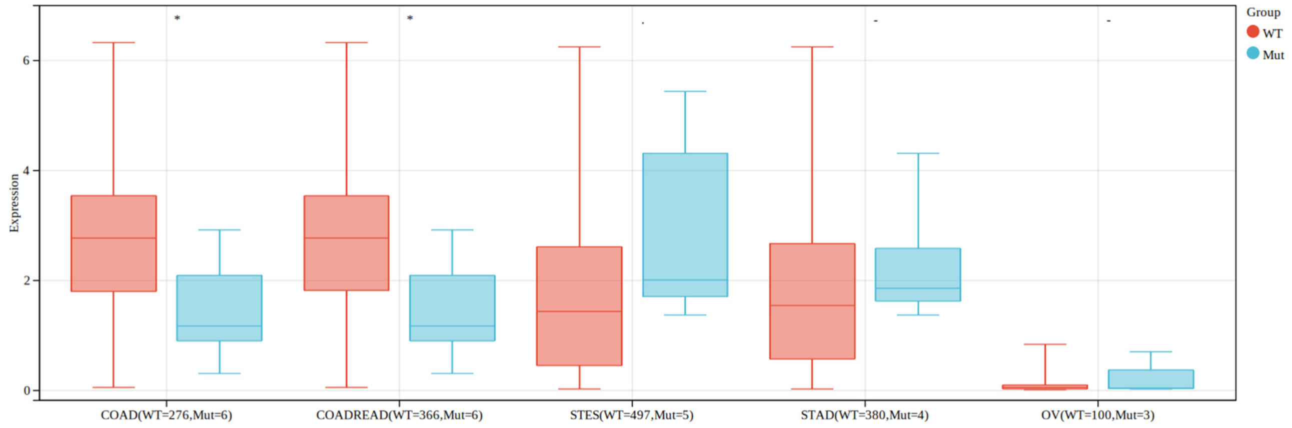

APOBEC2

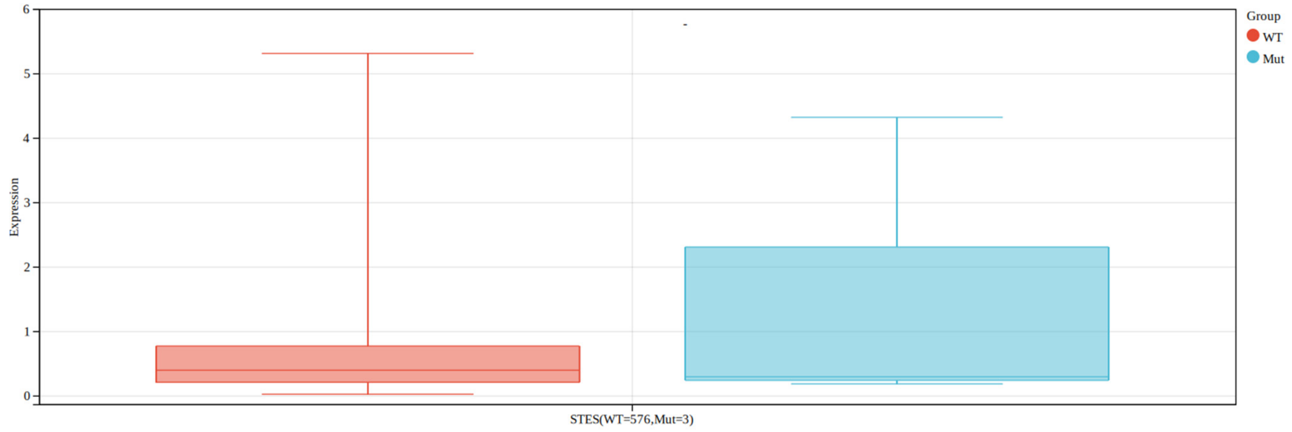

APOBEC3A

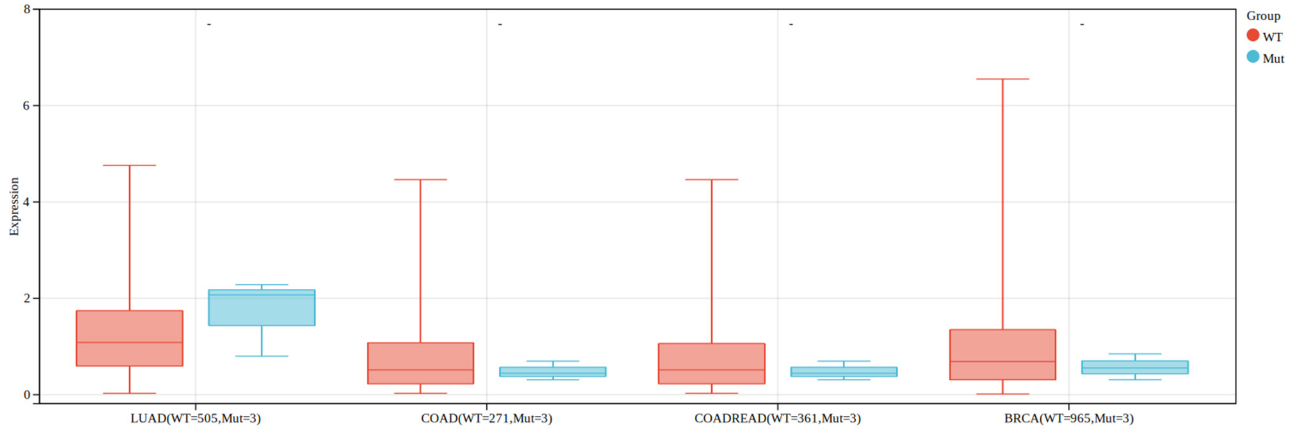

APOBEC3B

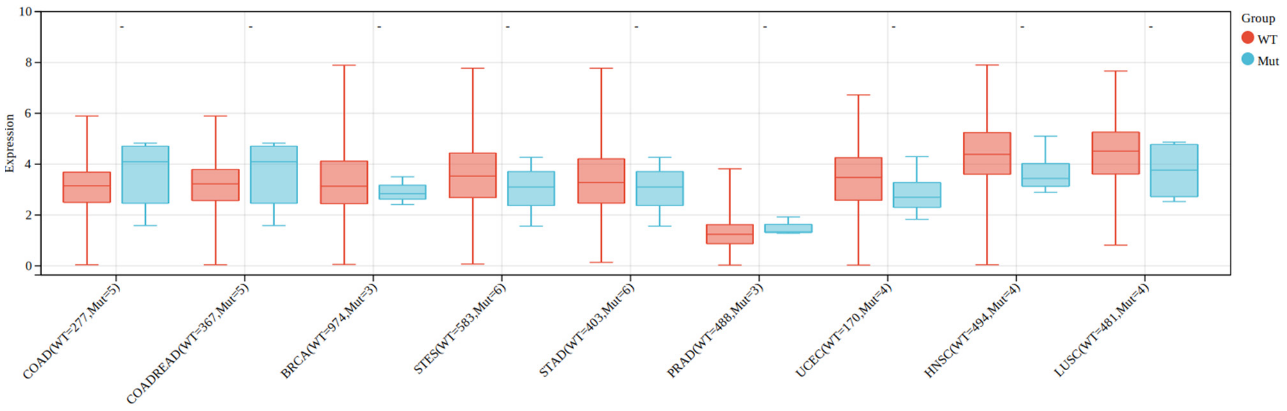

APOBEC3C

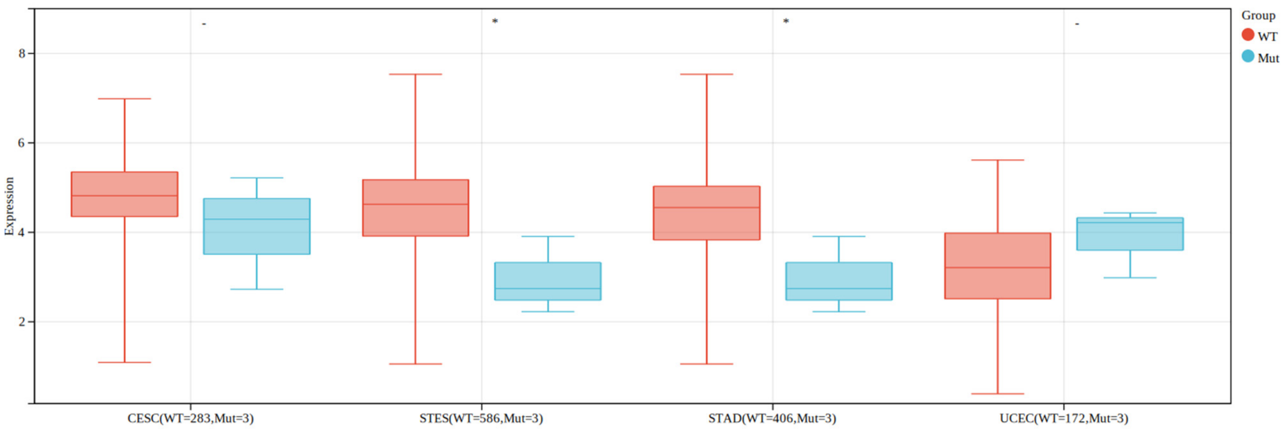

APOBEC3D

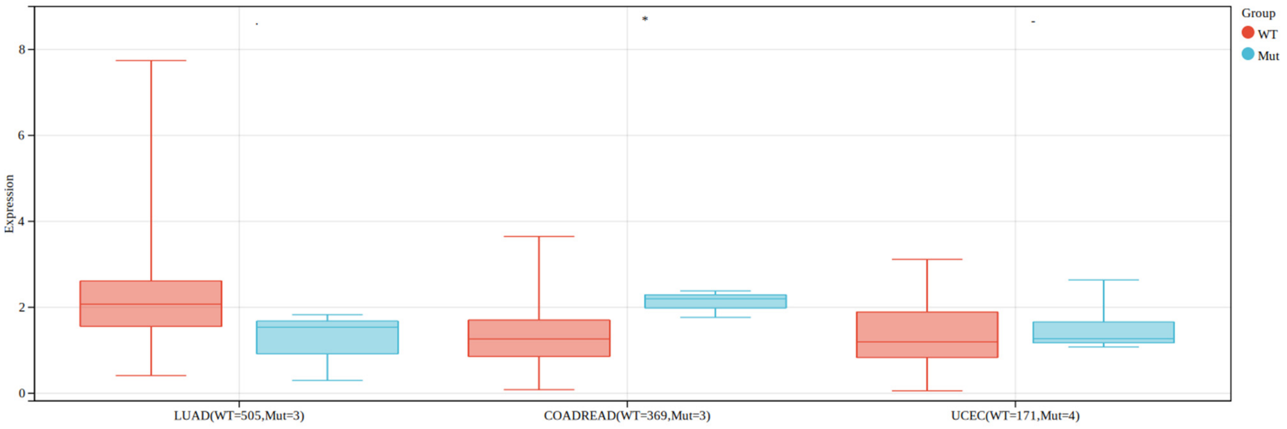

APOBEC3F

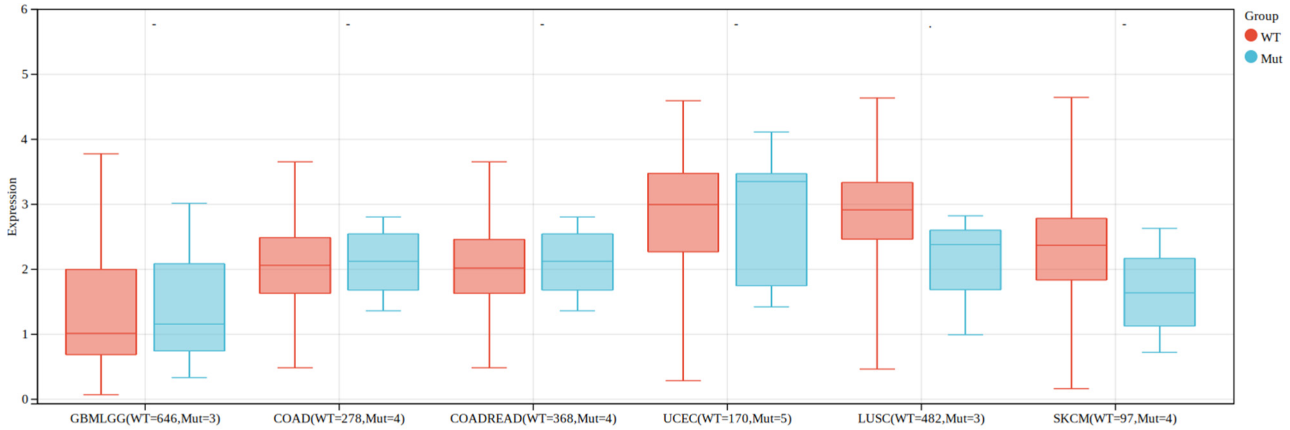

APOBEC3G

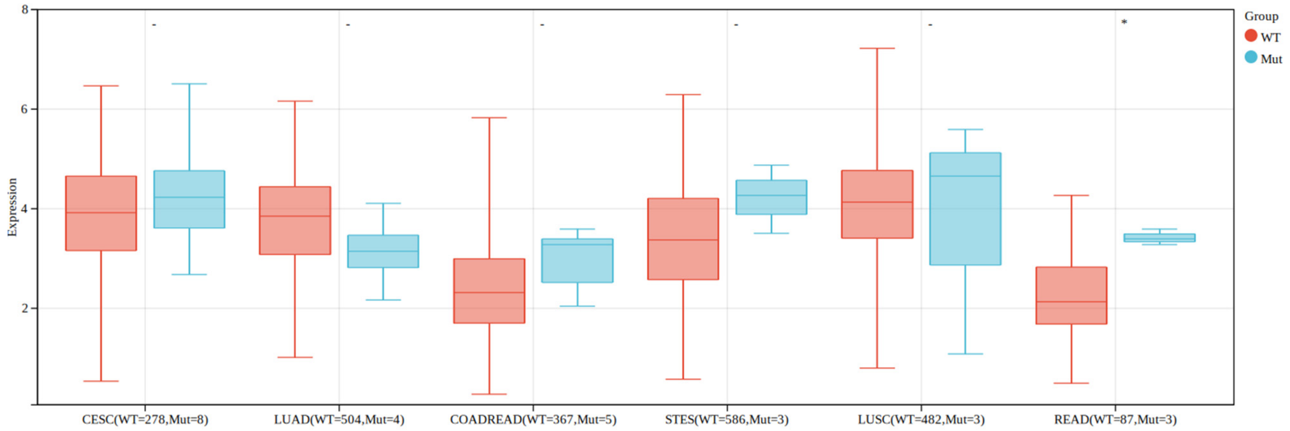

APOBEC3H

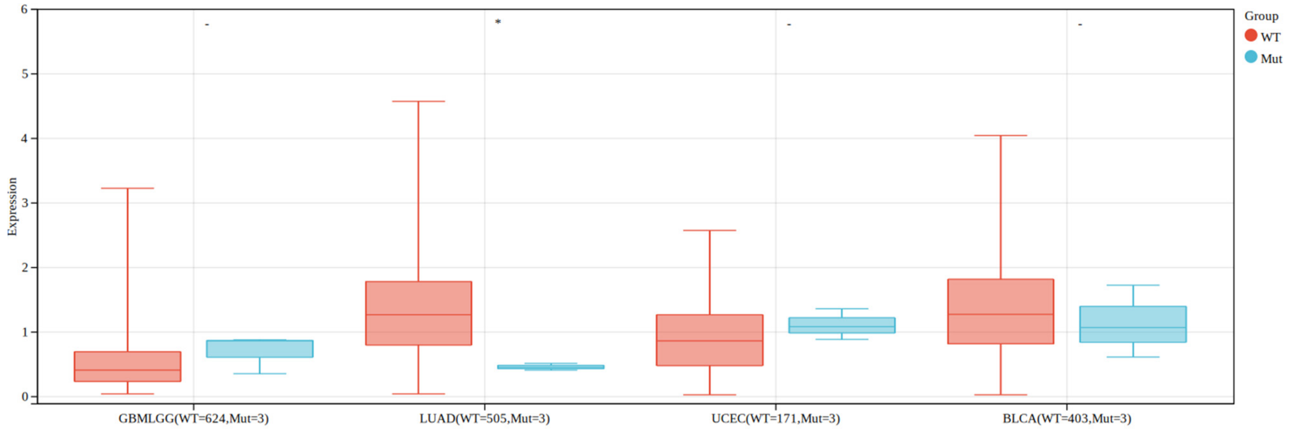

APOBEC4

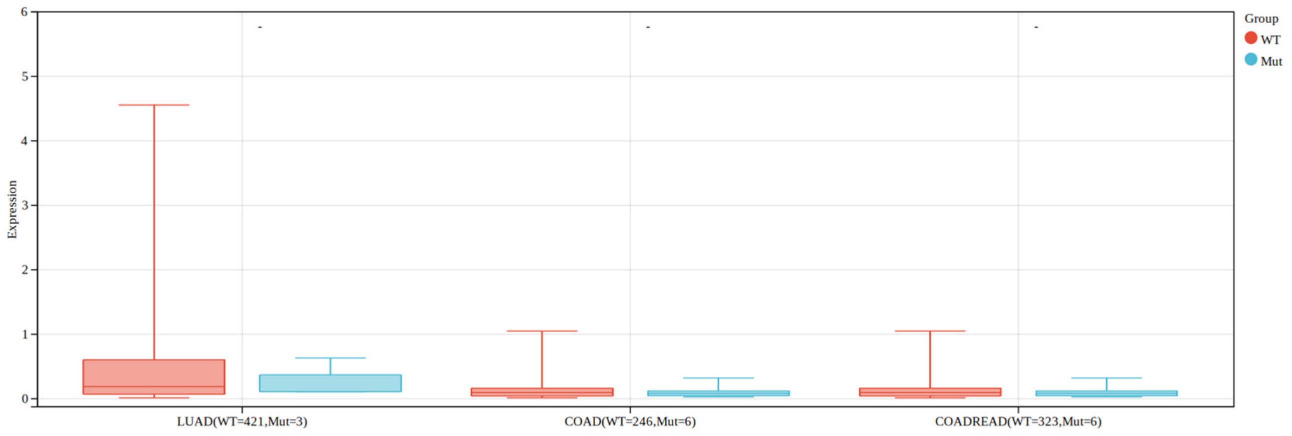

Table S12. The seven nominally significant mutation-status-versus-expression associations of 45; none survives correction.

Gene	Cancer Type	WT Mean ± SD	Mut Mean ± SD	p	Direction
APOBEC1	COAD	2.72 ± 1.22	1.46 ± 0.98	0.02	Mut < WT
APOBEC1	COADREAD	2.72 ± 1.19	1.46 ± 0.98	0.01	Mut < WT
APOBEC3C	STES	4.51 ± 1.02	2.96 ± 0.86	0.02	Mut < WT
APOBEC3C	STAD	4.41 ± 0.98	2.96 ± 0.86	0.02	Mut < WT
APOBEC3D	COADREAD	1.33 ± 0.64	2.12 ± 0.32	0.03	Mut > WT
APOBEC3G	READ	2.25 ± 0.78	3.42 ± 0.16	0.02	Mut > WT
APOBEC3H	LUAD	1.34 ± 0.71	0.46 ± 0.05	0.01	Mut < WT

Figure S11. Overlap of APOBEC enrichment and fitted SBS2/SBS13 exposure across 9,653 tumors.

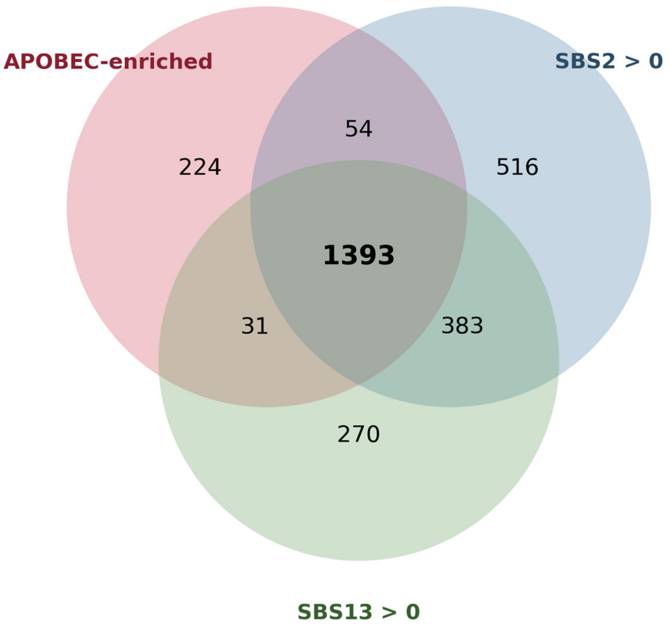

Figure S12. Cancer-specific APOBEC burden–mortality hazard ratios for the five major APOBEC-associated cancer types (BLCA, CESC, HNSC, LUAD, LUSC). Points are adjusted hazard ratios per doubling of APOBEC-attributed mutation burden (Cox model, age- and sex-adjusted) with 95% confidence intervals; the dashed line marks HR = 1. The direction of association differs across cancer types.

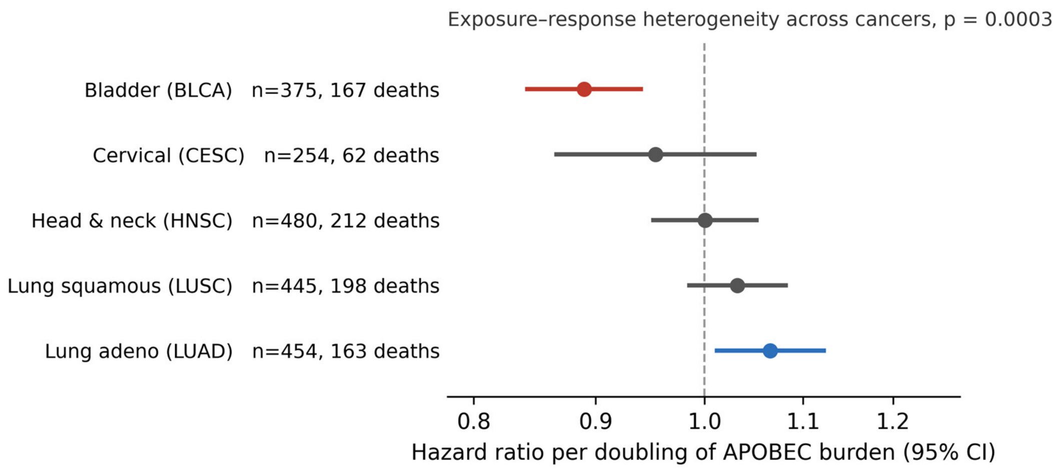

Table S13. Robustness of the APOBEC burden–overall survival association. Hazard ratios are from Cox models stratified by cancer type, evaluated at 10, 100, and 250 APOBEC-attributed mutations relative to zero; the nonlinearity p compares the restricted cubic spline (3 knots) against a log-linear term.

Model	N	Deaths	Nonlinearity p	HR@10	HR@100 (95% CI)	HR@250 (95% CI)
A. Age + sex (Figure 2)	9,244	2,856	<0.001	1.19	0.96 (0.84–1.10)	0.78 (0.63–0.95)
B. + total mutation burden	9,244	2,856	<0.001	1.18	1.01 (0.87–1.16)	0.85 (0.69–1.05)
C. + TMB + AJCC stage (staged subset)	5,848	1,774	<0.001	1.22	1.04 (0.89–1.22)	0.87 (0.68–1.10)
D. Positive burden only (>0)	2,532	864	<0.001	1.77	1.57 (0.87–2.82)	1.21 (0.69–2.12)

Leave-one-cancer-out (Model A, HR at 250 mutations).

Cohort	N	Deaths	Nonlinearity p	HR@250 (95% CI)
Full (all cancers)	9,244	2,856	<0.001	0.78 (0.63–0.95)
Drop BLCA	8,869	2,689	0.006	0.92 (0.72–1.17)
Drop CESC	8,990	2,794	<0.001	0.77 (0.62–0.96)
Drop HNSC	8,764	2,644	<0.001	0.77 (0.61–0.96)
Drop LUAD	8,790	2,693	<0.001	0.71 (0.57–0.89)
Drop LUSC	8,799	2,658	<0.001	0.73 (0.59–0.91)
Drop all 5 major	7,236	2,054	0.244	0.82 (0.46–1.47)

Sample size and deaths by burden interval.

APOBEC-attributed mutations	0	1–9	10–49	50–249	≥250
Tumors	6,712	679	1,017	672	164
Deaths	1,992	177	389	247	51

Proportional-hazards test (Schoenfeld, Model A): all $p > 0.38$ (age 0.38, burden 0.42, sex 0.86, spline 0.58); no violation. Exposure-response heterogeneity across the five major cancers: interaction likelihood-ratio $\chi^2 = 23.3$, df = 5, $p = 0.0003$.

Figure S13. Cancer-specific restricted-cubic-spline curves relating APOBEC burden to the overall-survival hazard ratio for the five major APOBEC-associated cancer types (BLCA, CESC, HNSC, LUAD, LUSC), each fitted separately and adjusted for age and sex, with 95% confidence intervals. Knots were placed at the pooled 10th, 50th, and 90th percentiles of non-zero burden (5, 23, and 172 mutations). The reduction in hazard at high burden is seen mainly in bladder urothelial carcinoma (BLCA), whereas lung adenocarcinoma (LUAD) shows increasing hazard with burden.

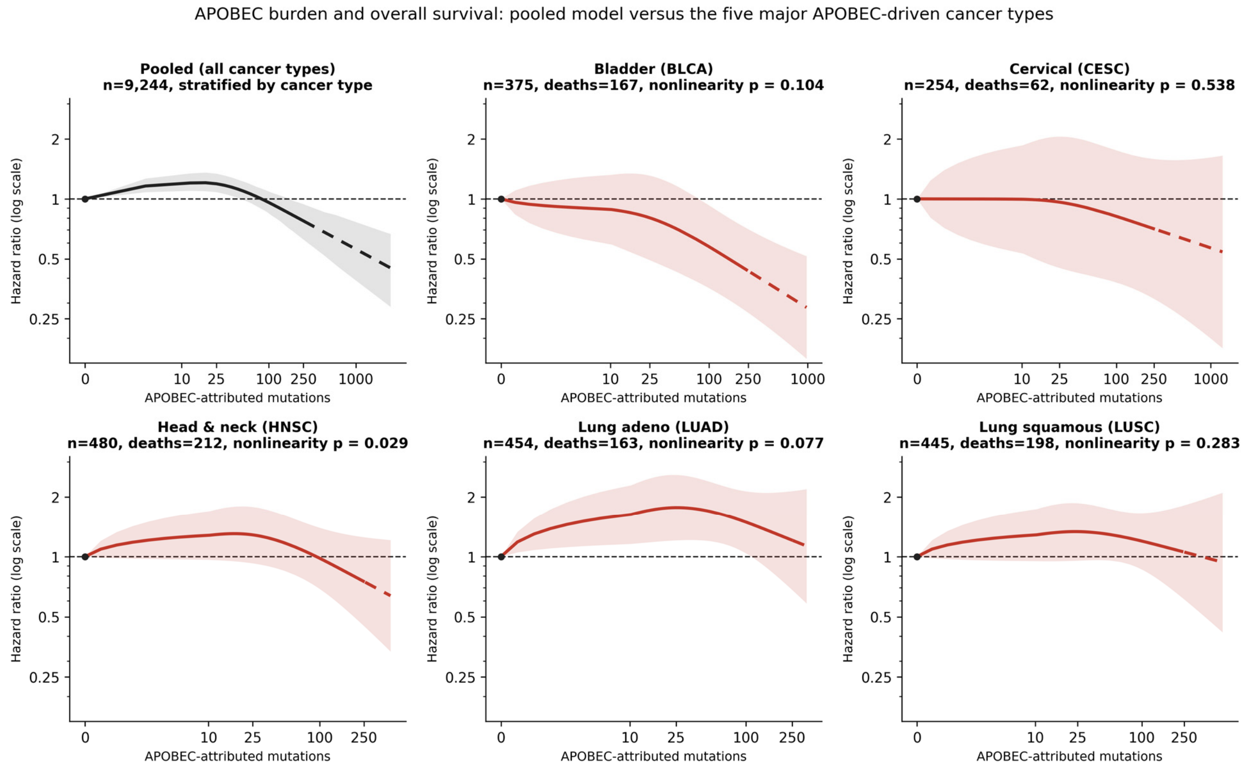

Table S14. Immune correlations significant before and after Benjamini–Hochberg (BH) correction.

Analysis	Tests	p < 0.05	BH q < 0.05	Positive after correction (%)
ESTIMATE	1,305	799	765	92.8
TIMER	2,250	1,291	1,220	93.9
CIBERSORT	9,406	2,655	1,729	54.3
Immune checkpoints	21,391	12,742	12,009	94.0

Table S15. Immune correlations before and after adjustment for tumor purity (TCGA tumors; BH q < 0.05).

**Analysis**	**Significant before adjustment**	**Significant after adjustment, n (%)**	**Same direction (%)**
ESTIMATE	588	469 (79.8)	98.1
TIMER	914	702 (76.8)	98.9
CIBERSORT	1,675	1,293 (77.2)	99.8
Immune checkpoints	9,505	7,866 (82.8)	99.4

Table S16. Tumor-versus-normal expression comparisons. (a) Tumor and normal samples per cancer type. (b) Number of cancer types with significant upregulation or downregulation (BH q < 0.05) against all normal samples and against TCGA tumor-adjacent normal tissue only (19 cancer types with ≥10 such samples).

**Cancer type**	**Tumor**	**TCGA tumor-adjacent normal**	**GTEx normal**
ACC	77	0	128
ALL†	194	0	337
BLCA	407	19	9
BRCA	1,099	113	179
CESC	306	3	10
CHOL	36	9	0
COAD	290	41	308
COADREAD	383	51	308
ESCA	182	0	653
GBM	166	5	1,152
GBMLGG	689	0	1,152
HNSC	520	44	0
KICH	66	25	0
KIPAN	884	129	28
KIRC†	530	72	0
KIRP†	288	32	0
LAML†	369	0	337
LGG	523	0	1,152
LIHC	371	50	110
LUAD†	515	59	288
LUSC	498	50	288
OV	427	0	88
PAAD	179	4	167

PCPG	179	3	0
PRAD	496	52	100
READ	93	10	0
SKCM†	468	1	556
STAD	414	36	174
STES†	596	49	174
TGCT	148	0	165
THCA	512	59	279
UCEC	181	23	0
UCS	57	0	78
WT†	126	6	0

† Approximate numbers.

Gene	All normal samples, up / down	TCGA tumor-adjacent normal only, up / down
APOBEC1	7 / 1	9 / 0
APOBEC2	1 / 16	4 / 15
APOBEC3A	7 / 10	7 / 8
APOBEC3B	18 / 1	15 / 4
APOBEC3C	11 / 7	7 / 7
APOBEC3D	10 / 7	10 / 8
APOBEC3F	15 / 2	10 / 3
APOBEC3G	13 / 6	7 / 7
APOBEC3H	11 / 3	11 / 2
APOBEC4	6 / 5	3 / 4

Table S17. Overall survival by APOBEC burden expressed as an absolute count or as the fraction of substitutions attributed to SBS2 and SBS13. Cox models stratified by cancer type and adjusted for age and sex; hazard ratios (95% CI) relative to no APOBEC-attributed mutations.

APOBEC burden	p for nonlinearity	HR, low	HR, moderate	HR, high
Absolute count (10 / 100 / 250 mutations)	2.3×10^{-6}	1.19 (1.08–1.31)	0.96 (0.84–1.10)	0.78 (0.63–0.95)
Fraction of substitutions (5% / 20% / 50%)	1.7×10^{-5}	1.03 (1.01–1.06)	1.14 (1.04–1.25)	1.04 (0.90–1.20)

Table S18. Spearman correlation (ρ) between APOBEC3A or APOBEC3B expression and APOBEC burden within cancer types; q, BH-corrected across cancer types.

**Cancer type**	**n**	**APOBEC3A ρ**	**q**	**APOBEC3B ρ**	**q**
ACC	68	+0.22	0.138	+0.20	0.213
BLCA	378	+0.20	<0.001	+0.31	<0.001
BRCA	972	+0.28	<0.001	+0.17	<0.001
CESC	266	+0.37	<0.001	+0.15	0.040
COAD	399	−0.02	0.877	0.00	0.987
ESCA	177	+0.28	<0.001	+0.25	0.003
GBM	154	−0.01	0.906	+0.02	0.838
HNSC	477	+0.24	<0.001	+0.14	0.007
KIRC	345	+0.08	0.237	+0.12	0.070
KIRP	260	+0.04	0.741	+0.15	0.034
LGG	487	−0.01	0.906	+0.03	0.645
LIHC	333	+0.08	0.237	+0.08	0.229
LUAD	472	+0.22	<0.001	+0.24	<0.001
LUSC	454	+0.26	<0.001	+0.14	0.008
MESO	73	−0.04	0.887	−0.11	0.513
OV	191	+0.12	0.179	+0.10	0.303
PAAD	161	−0.02	0.906	+0.12	0.238
PCPG	168	+0.06	0.611	+0.02	0.860
PRAD	452	+0.09	0.105	−0.02	0.838
SARC	220	+0.01	0.906	+0.06	0.513
SKCM	421	−0.03	0.775	+0.02	0.838
STAD	378	+0.11	0.075	+0.23	<0.001
TGCT	134	−0.13	0.221	+0.03	0.838
THCA	459	+0.14	0.008	+0.12	0.034
UCEC	498	+0.27	<0.001	+0.29	<0.001
UCS	56	+0.28	0.082	+0.12	0.534
